# Rearrangement of Arylsulfamates and Sulfates to *Para*-Sulfonyl Anilines and Phenols

**DOI:** 10.3390/molecules29071445

**Published:** 2024-03-23

**Authors:** Yifei Zhou, Alan M. Jones

**Affiliations:** School of Pharmacy, University of Birmingham, Edgbaston, Birmingham B15 2TT, UK

**Keywords:** sulfation, sulfonation, sulfamation, rearrangement, TBSAB, Tyrer

## Abstract

The *C*(sp^2^)-aryl sulfonate functional group is found in bioactive molecules, but their synthesis can involve extreme temperatures (>190 °C or flash vacuum pyrolysis) and strongly acidic reaction conditions. Inspired by the 1917 Tyrer industrial process for a sulfa dye that involved an aniline *N*(sp^2^)-SO_3_ intermediate *en route* to a *C*(sp^2^)-SO_3_ rearranged product, we investigated tributylsulfoammonium betaine (TBSAB) as a milder *N*-sulfamation to *C*-sulfonate relay reagent. Initial investigations of a stepwise route involving TBSAB on selected anilines at room temperature enabled the isolation of *N*(sp^2^)-sulfamate. Subsequent thermal rearrangement demonstrated the intermediary of a sulfamate *en route* to the sulfonate; however, it was low-yielding. Investigation of the *N*-sulfamate to *C*--sulfonate mechanism through control experiments with variation at the heteroatom positions and kinetic isotope experiments (KIE^H/D^) confirmed the formation of a key *N*(sp^2^)-SO_3_ intermediate and further confirmed an *inter*molecular mechanism. Furthermore, compounds without an accessible nitrogen (or oxygen) lone pair did not undergo sulfamation- (or sulfation) -to-sulfonation under these conditions. A one-pot sulfamation and thermal sulfonation reaction was ultimately developed and explored on a range of aniline and heterocyclic scaffolds with high conversions, including *N*(sp^2^)-sulfamates (*O*(sp^2^)-sulfates) and *C*(sp^2^)-sulfonates, in up to 99 and 80% (and 88% for a phenolic example) isolated yield, respectively. Encouragingly, the ability to modulate the *ortho-para* selectivity of the products obtained was observed under thermal control. A sulfonated analog of the intravenous anesthetic propofol was isolated (88% yield), demonstrating a proof-of-concept modification of a licensed drug alongside a range of nitrogen- and sulfur-containing heterocyclic fragments used in drug discovery.

## 1. Introduction

**Figure 1:**
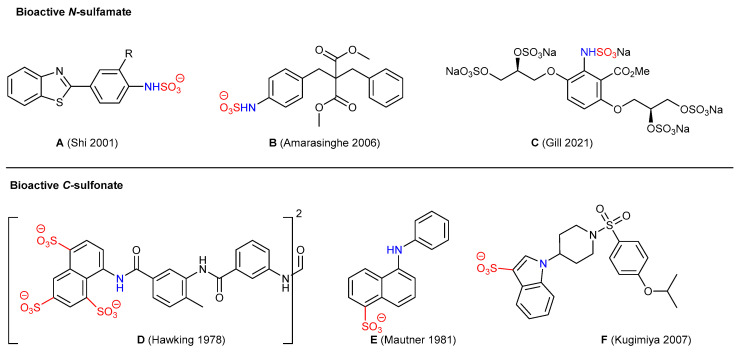
Structures of exemplar bioactive sulfamate and sulfonate containing molecules [1,2,3,6,7,8].

Sulfamated (*N*(sp^2^)-SO_3_) and sulfonated (*C*(sp^2^)-SO_3_) arylated motifs are found in a variety of valuable commodities, including sulfa dyes, sulfa drugs, and bioactive molecules (Figure 1). 

Examples of bioactive *N*(sp^2^)-sulfamates include (A) a sulfamate salt prodrug derivative of the potent and selective 2-(4-aminophenyl)benzothiazole anticancer agent [1]; (B) a malonate templated sulfamic acid phosphotyrosine mimetic as a selective and potent inhibitor of HPTPβ (a protein tyrosine phosphatase) [2]; (C) a glycomimetic that has protective effects against lipid-induced endothelial dysfunction, restorative effects on diabetic endothelial colony forming cells, and preventative effects on downstream vascular calcification [3,4,5]. Examples of bioactive *C*(sp^2^)-sulfonates include (D) suramin, a medication for treating river blindness and African sleeping sickness [6]; (E) an inhibitor against the coenzyme A binding site of choline acetyltransferase [7]; and (F) an indole derivative possessing PGD2 receptor antagonist activity [8].

**Scheme 1:**
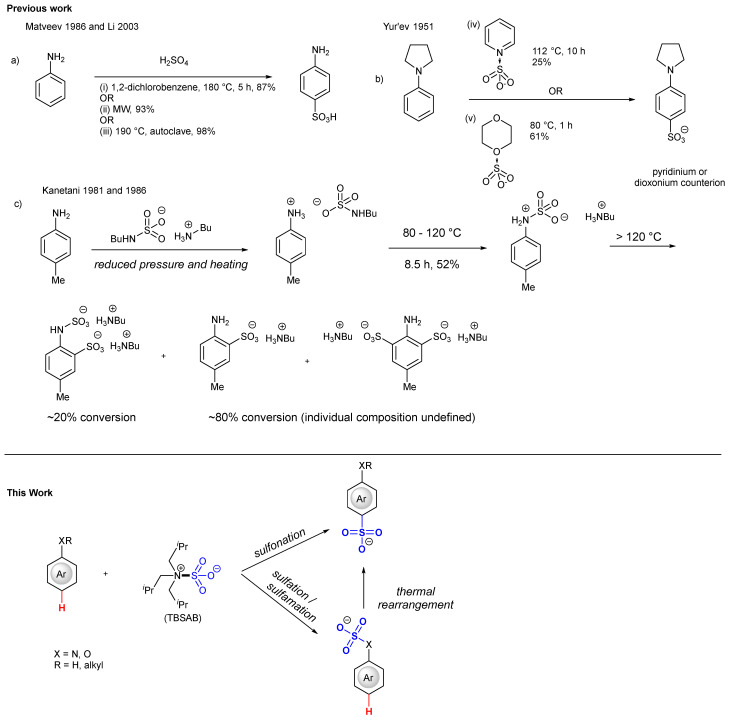
*Previous Approaches* (**a**–**c**) towards the synthesis of *p*-aminobenzene sulfonic acid compounds using an *N*- to *C*-SO_3_ transfer and this study: TBSAB is used to introduce the SO_3_ group into aromatic systems. The tetrabutylammonium cation is omitted for clarity [16,17,18,19,20].

In turn, methods to prepare these *N*(sp^2^)-aryl sulfamate precursors under mild, non-corrosive conditions are limited [9,10,11,12,13,14,15], and *C*(sp^2^)-sulfonated compounds are only achievable under more forcing conditions (Scheme 1) [16,17,18,19,20]. 

Benedetti [9] has reported one example of an *N*-sulfamation reaction on an unsubstituted, *N*-substituted, and *N*,*N*-disubstituted aniline with TBSAB in 50–90% isolated yields. Alshehri [10] has reported a single example of unsubstituted aniline sulfamation with the Me_3_N-SO_3_ complex in 57% isolated yield. Blackburn [11] has reported three examples of *N*-aryl sulfamates employing Py-SO_3_ or Me_3_N-SO_3_ complexes in 94–95% isolated yield. Kanetani [12] has reported a single example of the insertion of sulfur trioxide into the N-Si bond of anilinotrimethylsilane to yield phenylamidosulfate as a mixture of salts in 51% isolated yield. Within the patent literature [13], there is a direct method to insert sulfur trioxide gas with four aniline examples (i.e., aniline, *para*-methyl, *para*-chloro, and *ortho*-methyl aniline). Most recently, Phipps has used the direct action of chlorosulfonic acid on a range of anilines, with 9 examples (42–94% isolated yield) [14] and 33 examples (11–99% yield), respectively [15].

Solely on an unfunctionalized aniline, Mateev [16] and Li [17] have reported that the direct action of sulfuric acid at high temperatures delivers the *para*-sulfonylaniline. Yur’ev [18] has reported the action of the Py-SO_3_ complex on 1-phenylpyrrolidine in a 25% yield or the unstable dioxane-SO_3_ complex in a 61% yield. Kanetani [19,20] studied a *para*-blocked aniline leading to a complex mixture of sulfamated and variously sulfonated products, under flash vacuum pyrolysis conditions without isolation. Thus, there is much scope for improvement of methods to 1. *N*-sulfamate aryl molecules and 2. rearrange to the *C*-sulfonate.

Furthermore, the mechanism by which sulfur trioxide (SO_3_) is transferred in a S_E_Ar reaction from a sulfamate to afford this type of aryl *C*(sp^2^)-sulfonate has been of perennial interest and reinvestigated by several groups and is accepted as an *inter*molecular rearrangement [21,22,23,24,25,26,27,28,29,30,31,32,33]. 

Inspired by the Tyrer process for *C*(sp^2^)-sulfonated aryl systems via an *N*(sp^2^)-arylsulfamate [34,35,36,37,38,39], we considered whether the mild sulfating reagent tributylsulfoammonium betaine (TBSAB) [40,41] would give rise to different reactivity profiles via the in situ *N*-tributyl ammonium counterion effect [14,15,42] and a milder preparation of *C*-sulfonated molecules.

## 2. Results and Discussion

**Scheme 2:**
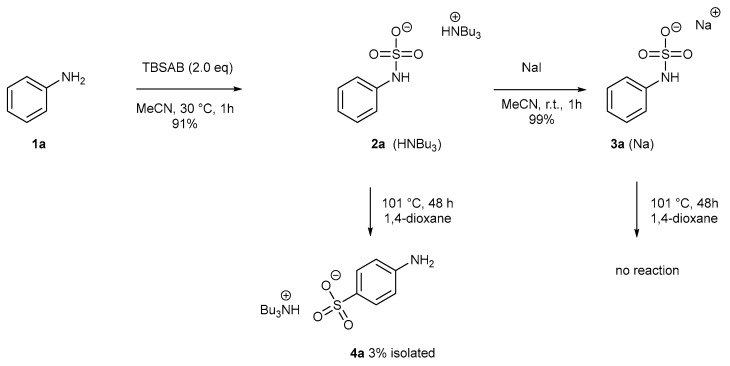
Initial attempts to prepare aniline sulfamates as their tributylammonium (**2a**) and sodium (**3a**) salts and resulting thermal rearrangement outcomes.

Our initial investigations focused on preparing the key *N*(sp^2^)-aniline sulfamate as both the tributylammonium (**2a**) and sodium (**3a**) salts to explore counterion effects on *ortho/para* selectivity (Scheme 2). TBSAB was prepared according to the procedure of Gill et al. [41].

Following a reported example of aniline sulfamation using TBSAB [9], we were able to prepare **2a** in 91% yield as its tributylammonium salt (Scheme 2). Treatment of **2a** with sodium iodide afforded the corresponding sodium salt, **3a**, in quantitative yield. Refluxing **2a** and **3a** in 1,4-dioxane, a detectable amount of the *para*-rearrangement product (**4a**, 3% isolated yield) as the tributylammonium salt and no rearrangement with the sodium counterion, respectively. This tentatively indicated the suitability of the tributylammonium counterion for further exploration.

**Scheme 3:**
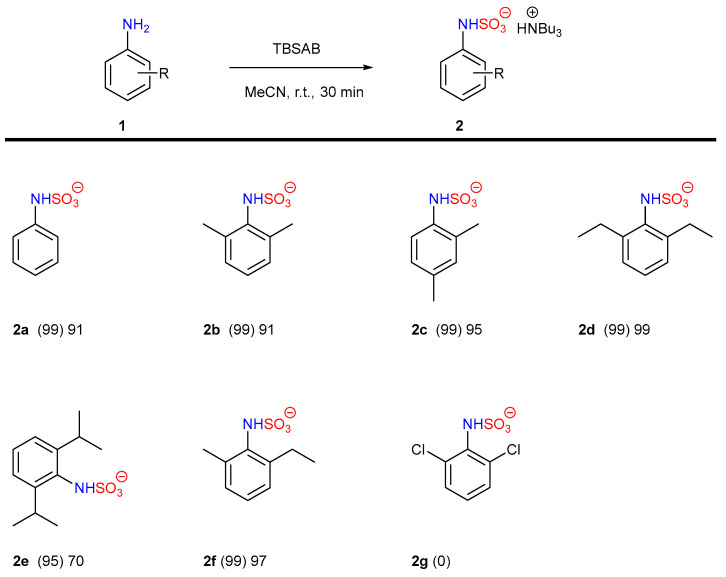
Synthesis of *N*(sp^2^)-anilino sulfamates using TBSAB. The tributylammonium cation is omitted for clarity. Key: percentage conversion as measured by ^1^H NMR spectroscopy is reported in parentheses, and isolated yield thereafter.

To probe the rearrangement ability of the aniline core, a range of *N*(sp^2^)-sulfamated anilines were synthesized using TBSAB as the sulfamating agent (Scheme 3) in 95–99% conversions and 70–99% isolated yield. The sterically encumbered 2,6-dichloroaniline example (**2g**) proved recalcitrant to sulfamation under these conditions. Examples selected varied the steric bulk *ortho* to the *ipso* aniline nitrogen from hydrogen < methyl < ethyl < isopropyl. To avoid the complexity of simultaneous *ortho* product formation, both *ortho* positions were blocked, except for **2c**. 

**Scheme 4:**
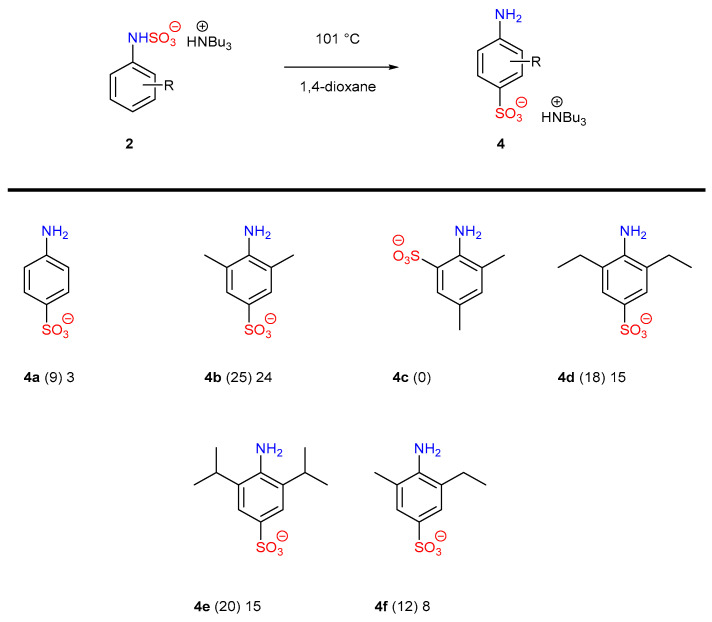
Initial attempts to access 4-aminobenzene sulfonate compounds by thermal rearrangement of **2**. The tributylammonium cation is omitted for clarity. Key: percentage conversion as measured by ^1^H NMR spectroscopy is reported in parentheses, and isolated yield thereafter.

Thermal treatment of the successful sulfamated examples (**2**) led to low to modest conversions of the sulfonated product (Scheme 4). All structures where the *para* site was accessible afforded an isolable (3–24% yield) of the *para*-sulfonated product. The *ortho*-accessible analog (**4c**) did not form under these conditions, despite similar electron-rich electronics to **4b**. Instead, under these conditions, we were able to regenerate TBSAB and the starting material aniline, demonstrating the reversibility of the formation of TBSAB for the first time.

**Scheme 5:**
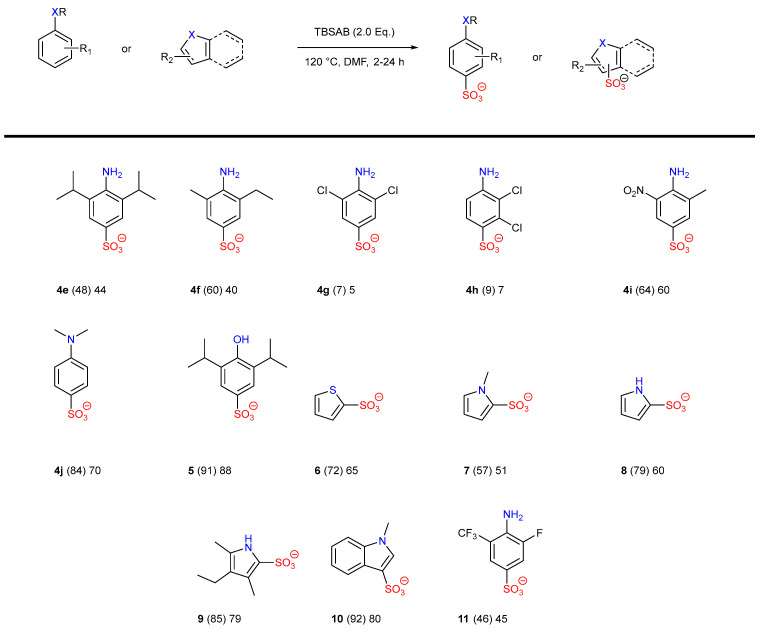
Reaction scope on aryl ring systems via a one-pot reaction. The tributylammonium cation is omitted for clarity. Key: percentage conversion as measured by ^1^H NMR spectroscopy is reported in parentheses, and isolated yield thereafter. X = O, N, S and R^1^/R^2^ = substituent variation.

Results where both *ortho* sites are blocked (**4b**, **4d**, **4e**, **4f**) agree with both the Illuminati [37] and Spillane [38,39] stepwise *inter*molecular mechanism—as an *ortho*-*para* sulfate walk is not possible. Due to the non-isolation of **2g** (Scheme 3), it was decided to react 2,6-chloroaniline directly with TBSAB and heat in a one-pot set-up. A low conversion (7%) and a 5% isolated yield of **4g** were found (see Scheme 5). The success of this challenging, sterically demanding, and electron-withdrawing example in a one-pot reaction led us to consider one-pot conditions for the direct reaction of anilines with TBSAB and in situ thermal rearrangement. Attempts to optimize the one-pot reaction on an aniline model system are shown in Table 1.

Entries 1–6 (Table 1) demonstrate that the highest conversion was observed with 2.0 equivalents of TBSAB (entry 4). Entry 6 (Table 1) shows that an inert atmosphere is preferred for the reaction. Entry 7 (Table 1) shows that no reaction occurs at a lower temperature. The use of polar protic solvents led to the unwanted breakdown of the *N*(sp^2^)-sulfamate to the aniline starting material (Chart 4, entries 8 and 9). This was confirmed via analogous treatment of an authentic sample of the sulfamate, ^1^H NMR spectroscopy, and thin-layer chromatography analysis.

Entries 10–13 (Table 1) detail the use of DMF as the solvent and varying the reaction temperature. With increasing temperature, the higher conversions were found, with an optimum at 120 °C (entry 12). Higher temperatures (>120 °C) were found to lead to more *ortho*-substituted product, for example, selectivity (*para*: *ortho*) decreased from 10:1 to 5:1. Entries 14–17 (Table 1) detail the use of DMSO as the solvent. Although entry 14 was comparable to the optimal DMF result, the complications of removing DMSO led to this being discontinued. Entries 18–21 (Table 1) detail the use of 1,2-dichlorobenzene as the solvent. Similarly, entry 18 was comparable and gave a comparable *para*: *ortho* ratio to DMF (entry 12), but difficulties removing this solvent ruled out further investigation.

Furthermore, in both the DMSO and 1,2-dichlorobenzene examples, evidence for the degradation of TBSAB was found above 160 °C (^1^H NMR spectroscopic analysis). With the optimal conditions for a one-pot *para*-selective S_E_Ar identified, substituted anilines, heterocycles, and oxygen-containing systems were screened (Scheme 5).

The one-pot method was applied to compounds **4e**, **4f**, and **4g** (Scheme 5), which resulted in improvements in conversion and isolated yield compared to the stepwise procedure (Scheme 3 and Scheme 4). Herein, **4e** increased from a linear 11% yield to 44%, **4f** increased from a linear 18% yield to 40%, and **4g** increased from no reaction to a 5% isolated yield. A regioisomer of **4g** gave a similar low yield of 7% (**4h**), demonstrating the deactivating effect of the di-chloro-aryl ring system. However, other electron-withdrawing groups are well tolerated. The nitro-containing example (**4i**) proceeded with a 64% conversion (60% isolated). 

*N*,*N*-dimethylaniline proceeded smoothly to afford the *para*-substituted sulfonate in 70% isolated yield (**4j**). Moving to other heteroatoms, the hydroxyl group of the sterically demanding i.v. anesthetic, propofol, was readily sulfonated in an 88% isolated yield (**5**). Thiophene was readily sulfonated in the 2-position (**6**) with a 65% yield. Protected (**7**) and unprotected pyrrole (**8**) were sulfonated in 51 and 60% yields, respectively. A tetrasubstituted pyrrole (**9**) was prepared with an excellent 79% yield, and *N*-methylindole (**10**) was sulfonated at the C3 position with an 80% isolated yield. Furthermore, a fluorine-containing building block was readily sulfonated in 45% isolated yield (**11**). In turn, these sulfonated (hetero)aryl systems can be further manipulated to produce sulfonyl chlorides, sulfonamides, and sulfinates as building blocks in medicinal chemistry applications.

## 3. Control Experiments

The rearrangement mechanism of an unsubstituted aniline sulfamate to the corresponding *para*-aniline sulfonate is believed to proceed via an *inter*molecular rearrangement. Radiolabeling experiments with H_2_^35^SO_4_ demonstrated that the sulfamate was desulfamated to sulfur trioxide during the rearrangement via radiolabel dilution [37,38,39]. This prior study has ruled out a stepwise *ortho*-*para intra*molecular sulfonate walk. 

Using a pragmatic approach, for example, by blocking the *ortho*-aniline positions, we have experimentally confirmed that an *intra*molecular movement of the sulfur group does not occur (e.g., **4e**, **4f**, **4g,** and **4i**) in more complex substituted examples.

**Scheme 6:**
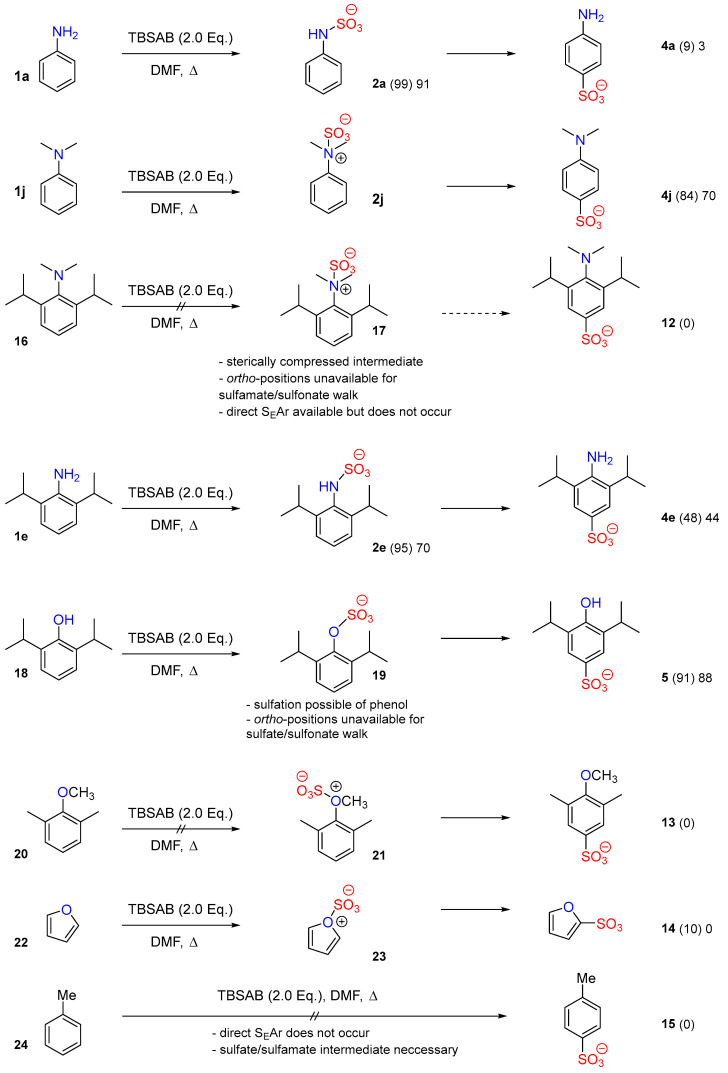
Control experiment results. Dashed arrow indicates hypothetical product.

However, a question remained as to whether an *N*-sulfamate is indeed a necessary intermediate for the overall sulfonation reaction with TBSAB to afford the *C*-sulfonate product (Scheme 6). For instance, does sulfonation occur directly with TBSAB via S_E_Ar, or is the *N*-sulfamate a critical intermediate?

In comparison to aniline (**1a**), *N*,*N*-dimethylaniline (**1j**) proceeded smoothly to afford the para-sulfonate **4j** in a 70% isolated yield (84% conversion as measured by ^1^H NMR spectroscopy). The molecularly matched pair (MMP), *N*,*N*-dimethylaniline analog (**16**) to the successful propofol (**18**) example did not show any evidence of the desired reaction by ^1^H NMR spectroscopic analysis of the crude reaction product. Molecular modeling demonstrated how sterically compressed the sulfamate would be sandwiched between di-*ortho*-isopropyl groups [43,44]. Thus, in this example, it can be concluded that sulfamation is necessary prior to sulfonation.

Replacing the phenol in the propofol example (**5**, 91% conversion (88% isolated)), with a similar but less sterically demanding methoxy example (**13**) resulted in only a trace conversion to the *para*-sulfonated **13** (as measured by time-course ^1^H NMR spectroscopy). The need for an available hydroxyl group can be further ascribed to the results of furan (**22**). A range of conditions were applied (r.t. to 85 °C) and solvents (DCM, MeCN, and 1,2-DCE), and a maximal 10% conversion was observed. Isolation of the sulfated furan (**14**) was further complicated by the presence of residual TBSAB (23% *w/w* impurity by ^1^H NMR spectroscopy).

To probe whether sulfonation of the aryl system is possible without a heteroatom, toluene was treated under the optimal aniline conditions (TBSAB, 120 °C, DMF, 24 h), and no trace of **15** was observed in the crude sample by ^1^H NMR spectroscopy, ruling out a direct S_E_Ar *C*-sulfonation mechanism with the TBSAB reagent.

**Scheme 7:**
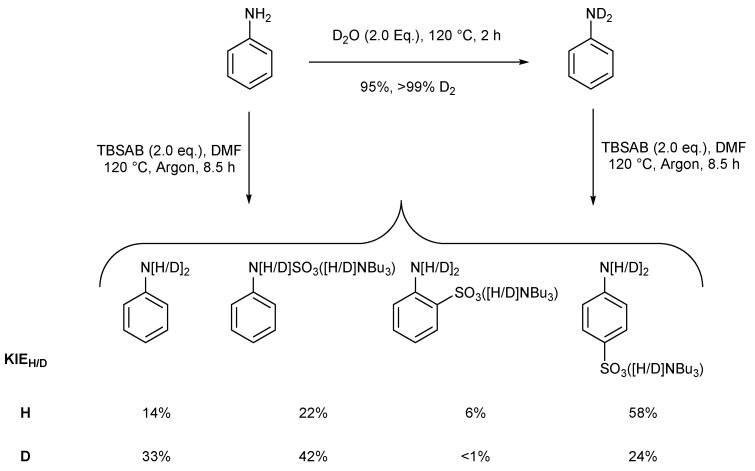
KIE^H/D^ effects on aniline sulfamation/sulfonation. Percentage conversion measured by ^1^H NMR spectroscopic analysis.

To further prove the requirement for *N*-sulfamation to occur prior to sulfonation, a kinetic isotope experiment was devised with D_2_-aniline (Scheme 7). The conversion of both rearrangement and sulfamate intermediate products noticeably decreased with the presence of deuterium, which implies the rate-determining step of this reaction is the formation of the *N*-sulfamate (Table S1 and Figure S1).

**Scheme 8:**
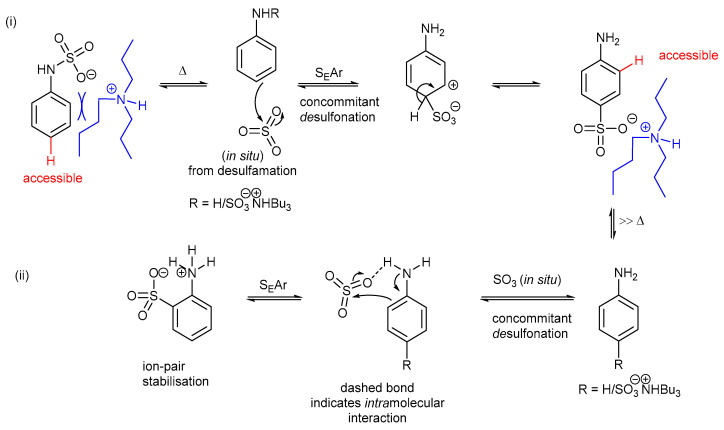
(**i**) A proposed mechanism for the intermolecular rearrangement process from *N*-*S* (sulfamate) to the *para*-*C*-*S* (sulfonate) position, and (**ii**) an observed increase in *ortho* selectivity under increased thermal conditions.

A proposed mechanism for (i) *para*-sulfonation and (ii) *ortho*-sulfonation is shown in Scheme 8. Sulfur trioxide is released from the *N*-sulfamate under thermal conditions, which then undergoes an S_E_Ar *inter*molecular reaction with the aniline to deliver the *para*-*C*-sulfonate product due to steric crowding at the *ortho* positions due to the tributylammonium cation effect. Upon prolonged high temperature, the *para*-*C*-sulfonate can reform aniline and sulfur trioxide *in situ*.Via intermolecular stabilization, an *ortho*-*C*-sulfonate product begins to form once sufficient energy input is reached into the system. With the advent of ohmic heating approaches [45] and alternative routes to *ortho*-sulfonates [46], this approach offers a mild route to *para*-sulfonates.

## 4. Conclusions

In this study, we have demonstrated that TBSAB is a mild aniline *N*-sulfamation (and phenol *O*-sulfation) reagent and a sulfamate (and sulfate) to sulfonate relay reagent. A range of aniline, phenol, and *N* and *S*-containing heterocyclic scaffolds were *C*-sulfonated in high conversions (6 examples of *N*(sp^2^)-sulfamates in up to 99% isolated yield and 16 examples of *C*(sp^2^)-sulfonate in up to 80% isolated yield) with the ability to change the *ortho-para* ratio of the products obtained under thermal control. A re-investigation of the *N*- to *C*-sulfate rearrangement mechanism through designed examples with variation at the heteroatom position and kinetic isotope experiments (KIE^H/D^) confirmed the necessity of an *N*-sulfamate (and *O*-sulfate) intermediate. The sulfonation reaction has also been exemplified on a drug molecule, demonstrating this approach as a route to incorporate this functionality at a late stage in more complex scaffolds. This manuscript was previously a ChemRxiv pre-print [47].

## Figures and Tables

**Table 1:**
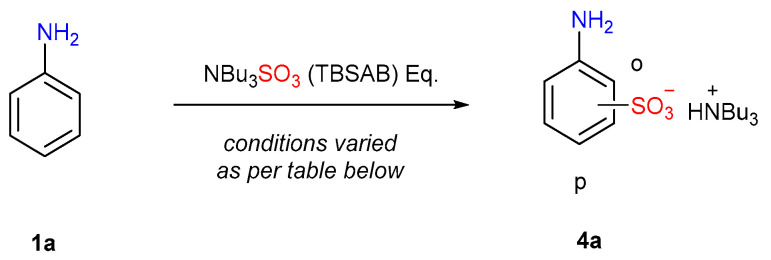
Optimization of an aniline model system. ^a^ Conversion and selectivity were determined by ^1^H NMR spectroscopy; ^b^ No reaction occurs below 80 °C; ^c^ Reaction products begin to decompose above 160 °C. Equivalents (Eq.) of TBSAB are varied. No reaction was observed below the reflux temperature of 1,4-dioxane (101 °C).

Entry	Eq.	T (°C)	Atmosphere	Solvent	*p*-4a at t = 2 h (%) ^a^	*p*-4a at t = 24 h (%) ^a^	Selectivity *p:o* ^a^
1	0.5	101	Ar	1,4-Dioxane	6	7	-
2	1.0	101	Ar	1,4-Dioxane	1	6	-
3	1.5	101	Ar	1,4-Dioxane	7	10	-
4	2	101	Ar	1,4-Dioxane	4	12	-
5	4	101	Ar	1,4-Dioxane	4	9	-
6	2	101	air	1,4-Dioxane	3	11	-
7 ^b^	2	80	Ar	1,4-Dioxane	-	-	-
8	2	100	Ar	Formic Acid	-	-	-
9	2	100	Ar	Butan-2-ol	-	-	-
10	2	80	Ar	DMF	-	-	-
11	2	100	Ar	DMF	4	13	-
12	2	120	Ar	DMF	32	58	>10:1
13	2	140	Ar	DMF	30	49	5:1
14	2	120	Ar	DMSO	25	48	8:1
15	2	140	Ar	DMSO	15	35	2:1
16	2	160	Ar	DMSO	-	-	-
17	2	180	Ar	DMSO	-	-	-
18	2	120	Ar	1,2-dichlorobenzene	27	52	>10:1
19	2	140	Ar	1,2-dichlorobenzene	20	44	4:1
20 ^c^	2	160	Ar	1,2-dichlorobenzene	-	-	-
21 ^c^	2	180	Ar	1,2-dichlorobenzene	-	-	-

## Data Availability

Data are contained within the article and Supplementary Materials.

## Supplementary Materials

The following supporting information can be downloaded at: https://www.mdpi.com/article/10.3390/molecules29071445/s1. See supporting information for characterization data on all compounds and accompanying ^1^H, ^13^C, and ^19^F NMR spectra.
