# Supplementary material for: Rearrangement of Arylsulfamates and Sulfates to Para-Sulfonyl Anilines and Phenols
Source: Molecules. 2024 Mar 23;29(7):1445. doi: 10.3390/molecules29071445 (PMC11013102; doi:10.3390/molecules29071445)
Supplement: Supplementary file 1 [file molecules-29-01445-s001.zip › molecules-2926470-supplementary.pdf]

SUPPORTING INFORMATION

Rearrangement of Arylsulfamates and Sulfates To *Para*-Sulfonyl Anilines and Phenols

Yifei Zhou,[1] Alan M. Jones[1*]
[1] Molecular Synthesis Laboratory, School of Pharmacy, University of Birmingham, Edgbaston, Birmingham, B15 2TT, United Kingdom
* Corresponding author: (A.M.J.) a.m.jones.2@bham.ac.uk; +44(0)121-414-7288

CONTENTS

GENERAL CONSIDERATIONS PAGE 2
GENERAL PROCEDURES PAGE 2
COMPOUND CHARACTERISATION PAGE 3
Copies of ^{1}H, ^{13}C, ^{19}F NMR spectra PAGE 15
Sulfonation of Aniline-D_2 PAGE 63

GENERAL CONSIDERATIONS

All reactions involving moisture sensitive reagents were carried out using standard Schlenk techniques, in a dry reaction vessel under argon. All solvents used under anhydrous conditions were decanted directly from an SPS dispensary or were stored over 4 Å molecular sieves 24 h prior to use.
Solvents used for workup procedures were of technical grade from Sigma-Aldrich, Honeywell, VWR or Fisher Scientific. Unless stated otherwise, solvents were removed by rotary evaporation under reduced pressure between 30-50 °C. All chemical reagents were used as received unless stated otherwise. Reactions were monitored by TLC analysis on Merck silica gel 60 F254 using UV light (254 nm) and/or potassium permanganate.
^{1}H and ^{13}C NMR spectra were recorded either on a Bruker AVIII operating at 300 MHz for ^{1}H and fitted with a 5mm BBFO probe or on a Bruker AVANCE NEO operating at 400 MHz for ^{1}H fitted with a 5mm "smart" BBFO probe, respectively. ^{1}H-^{1}H COSY, DEPT-45, ^{1}H-^{13}C HSQC, and ^{1}H-^{13}C HMBC NMR spectra were recorded on a Bruker AVANCE NEO console operating at 400 MHz for 1H and fitted with a nitrogen-cooled BBFO probe. Chemical shift data for ^{1}H are reported in parts per million (ppm, δ scale) downfield from tetramethylsilane (TMS: δ 0.0) and referenced internally to the residual proton in the solvent. The deuterated solvents used for NMR analysis were chloroform ($CDCl_3$: δH 7.26, δC 77.2), methanol (MeOD: δH 3.31, δC 49.2) and dimethyl sulfoxide (DMSO-d_6: δH 2.50, δC 39.5). Coupling constants (*J*) are given in hertz (Hz). The data are presented as follows: chemical shift, multiplicity (s = singlet, d = doublet, t = triplet, q = quartet, p = pentet, m = multiple, br = broad, app = apparent and combinations thereof), coupling constant and integration and assignment.
Mass spectra were recorded on a Waters Xevo G2-XS ToF or Synap G2-S mass spectrometer using Zspray, Electro-spray ionization in negative (ESI^-) and positive (ESI^+) mode, respectively.

GENERAL PROCEDURES

General procedure 1. Synthetic procedure for the preparation of phenylsulfamates using tributyl sulfoammonium betaine (Bu_3NSO_3, TBSAB)

$$RNH_2 \xrightarrow[\text{MeCN, 30 °C}]{Bu_3NSO_3\ (2.0\ eq)} RNHSO_3^{-}\ \overset{+}{H}NBu_3$$

A flask was charged with amine (1.0 mmol) and TBSAB (2.0 eq) under argon. Anhydrous MeCN was added (giving a concentration of 0.50 Mol dm^{-3} to the limiting reagent), the reaction mixture was heated at 30 °C and monitored by TLC. After reaction completion, the flask was cooled to room temperature and the solvent removed under reduced pressure. The reaction was quenched with cold water (10 mL) and filtered. The aqueous solution was extracted with EtOAc (4 x 50 mL). The organic layer was dried ($MgSO_4$), filtered, and the solvent was removed in vacuo to afford the desired compound.

General procedure 2. Preparation of 4-aminobenzenesulfonate tributyl ammonium salt by using the phenylsulfamates from **General procedure 1**.

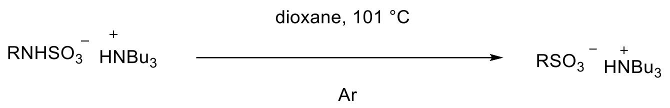

A flask was charged with amine (1.0 mmol) and TBSAB (2.0 eq) under argon. Anhydrous dioxane was added (giving a concentration of 0.50 Mol dm^{-3} to the limiting reagent), the reaction mixture was heated at 101 °C under reflux and monitored by TLC. After reaction completion, the flask was cooled to room temperature and the solvent removed under reduced pressure to give the desired product.

General Procedure 3. Sulfonation of Aromatic ring system containing heteroatoms

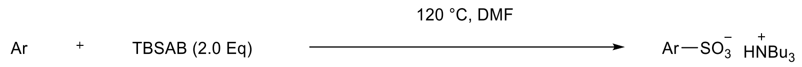

A flask was charged with starting material (4.0 mmol) and TBSAB (2.0 eq) under argon. Anhydrous DMF was added (giving a concentration of 0.50 Mol dm^{-3} to the limiting reagent), the reaction mixture was heated at 120 °C under reflux and monitored by TLC. After reaction completion, the flask was cooled to room temperature and the solvent removed under reduced pressure. The reaction was quenched with cold water (2 x 10 mL) and filtered. The aqueous solution was extracted with EtOAc (4 x 50 mL). The organic layer was dried ($MgSO_4$), filtered, and the solvent was removed *in vacuo* to give the desired product.

COMPOUND CHARACTERISATION

Tributylammonium phenylsulfamate (**2a**)

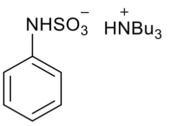

Following general procedure **1**: Aniline (0.09 mL, 1.0 mmol) and TBSAB (530 mg, 2.0 mmol) were dissolved in anhydrous MeCN (2.0 mL) and heated at 30 °C for 1 h. The title compound was extracted from cold water (10 mL) with EtOAc (4 × 50 mL) and washed with brine (10 mL). The organic layer was dried ($MgSO_4$), filtered, and evaporated *in vacuo* to afford the title compound as a white solid (326 mg, 91%).

M.P. 95-97 °C

IR ν_{max} cm^{-1} 3241w, 2958w, 2734w, 2652w, 1594w, 1490w, 1296w, 1222w, 1177w, 1028w

^{1}H NMR (300 MHz, $CDCl_3$) δ_H 9.85 (s, 1H), 7.26 – 6.88 (m, 5H), 2.99 – 2.88 (m, 6H), 1.62 (ddt, *J* = 11.1, 7.1, 4.0 Hz, 6H), 1.31 (h, *J* = 7.3 Hz, 6H), 0.92 (t, *J* = 7.3 Hz, 9H).

^{13}C NMR (101 MHz, $CDCl_3$) δ_C 141.5, 128.8, 121.5, 118.3, 52.3, 25.1, 20.0, 13.6
LRMS m/z (ESI-) 172.01 ($[M\text{-}Bu_3NH]^-$, 100%)
HRMS m/z (ESI-) $C_6H_6NO_3S$ requires 172.0180, found 172.0179 ($[M\text{-}Bu_3NH]^-$)

Tributylammonium 2,6-dimethylpheylsulfamate (**2b**)

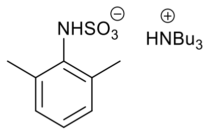

Following **general procedure 1**: 2,6-dimethylaniline (0.12 mL, 1.0 mmol) and TBSAB (531 mg, 2.0 mmol) were dissolved in anhydrous MeCN (2.0 mL) and heated at 30 °C for 1 h. The title compound was extracted from cold water (15 mL) with EtOAc (4 × 50 mL) and washed with brine (10 mL). The organic layer was dried ($MgSO_4$), filtered, and evaporated *in vacuo* to afford the title compound as a white solid (326 mg, 91%).

M.P. 122-124 °C
^{1}H NMR (300 MHz, $CDCl_3$) δ_H 9.79 (s, 1H), 7.00 – 6.91 (m, 3H), 5.44 (s, 1H), 2.74 – 2.67 (m, 6H), 2.46 (s, 6H), 1.53 – 1.42 (m, 6H), 1.24 (dt, *J* = 7.3 Hz, 6H), 0.89 (t, *J* = 7.3 Hz, 9H)
^{13}C NMR (101 MHz, $CDCl_3$) δ_C 137.7, 137.2, 128.3, 125.8, 52.2, 25.0, 20.1, 19.2, 13.7
LRMS m/z (ESI-) 200.14 ($[M\text{-}Bu_3NH]^-$, 100%)
HRMS m/z (ESI-) $C_8H_{10}NSO_3$ requires 200.1442, found 200.1440 ($[M\text{-}Bu_3NH]^-$)

Tributylammonium 2,4-dimethylpehnylsulfamate (**2c**)

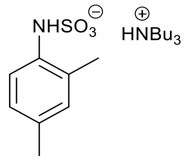

Following **general procedure 1**: 2,4-dimethylaniline (0.13 mL, 1.0 mmol) and TBSAB (531 mg, 2.0 mmol) were dissolved in anhydrous MeCN (2.0 mL) and heated at 30 °C for 1 h. The title compound was extracted from cold water (10 mL) with EtOAc (4 × 50 mL) and washed with brine (10 mL). The organic layer was dried ($MgSO_4$), filtered, and evaporated *in vacuo* to afford the title compound as a white solid (371.6 mg, 95.7 %).

M.P. 135-137 °C
^{1}H NMR (400 MHz, $CDCl_3$) δ_H 9.98 (s, 1H), 7.46 (d, *J* = 8.4 Hz, 1H), 6.92 (d, *J* = 7.1 Hz, 2H), 2.94 – 2.87 (m, 6H), 2.23 (s, 6H), 1.67-1.57 (m, 6H), 1.33 (h, *J* = 7.4 Hz, 6H), 0.94 (t, *J* = 7.4 Hz, 9H)

^{13}C NMR (101 MHz, $CDCl_3$) δ 136.9, 131.9, 131.0, 127.8, 127.2, 120.9, 52.4, 25.2, 20.8, 20.1, 17.9, 13.7
LRMS m/z (ESI-) 200.04 ($[M\text{-}Bu_3NH]^-$, 100%)
HRMS m/z (ESI-) $C_8H_{10}NSO_3$ requires 200.0442, found 200.0441 ($[M\text{-}Bu_3NH]^-$)

Tributylammonium 2,6-diethylphenylsulfamate (**2d**)

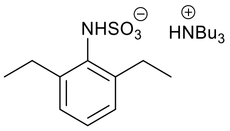

Following **general procedure 1**: 2,6-diethylaniline (0.32 mL, 2.0 mmol) and TBSAB (1.060 g, 4.0 mmol) were dissolved in anhydrous MeCN (2.0 mL) and heated at 30 °C for 1 h. The title compound was extracted from cold water (15 mL) with $CHCl_3$ (4 × 50 mL) and washed with brine (10 mL). The organic layer was dried ($MgSO_4$), filtered, and evaporated *in vacuo* to afford the title compound as a clear oil (820.3 mg, 99 %).

^{1}H NMR (400 MHz, $CDCl_3$) δ_H 9.94 (s, 1H), 7.11 – 7.03 (m, 3H), 3.00 (q, *J* = 7.6 Hz, 4H), 2.80 – 2.66 (m, 6H), 1.63 – 1.40 (m, 6H), 1.30 – 1.23 (m, *J* = 7.3 Hz, 6H), 1.19 (t, *J* = 7.6 Hz, 6H), 0.92 (t, *J* = 7.3 Hz, 9H).
^{13}C NMR (101 MHz, $CDCl_3$) δ_C 142.9, 136.1, 126.1, 126.0, 52.0, 24.9, 24.6, 20.0, 14.8, 13.5
LRMS m/z (ESI-) 228.07 ($[M\text{-}Bu_3NH]^-$, 100%)
HRMS m/z (ESI-) $C_{10}H_{14}NSO_3$ requires 228.0694, found 228.0699 ($[M\text{-}Bu_3NH]^-$)

Tributylammonium 2,6-diisopropylphenylsulfamate (**2e**)

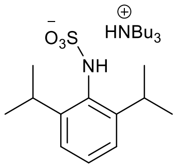

Following **general procedure 1**: 2,6-diisopropylaniline (0.19 mL, 1.0 mmol) and TBSAB (0.531 g, 2.0 mmol) were dissolved in anhydrous MeCN (2.0 mL) and heated at 30 °C for 4 h. The title compound was extracted from cold water (10 mL) with EtOAc (4 × 50 mL) and washed with brine (10 mL). The organic layer was dried ($MgSO_4$), filtered, and evaporated *in vacuo* to afford the title compound as a clear oil (308.6 mg, 70 %).

^{1}H NMR (400 MHz, $CDCl_3$) δ_H 10.09 (s, 1H), 7.16 – 7.07 (m, 3H), 3.90 (p, *J* = 6.9 Hz, 2H), 2.81 (m, 6H), 1.61 – 1.43 (m, 6H), 1.29 – 1.23 (m, *J* = 7.3 Hz, 6H), 1.17 (d, *J* = 6.9 Hz, 12H), 0.89 (t, *J* = 7.3 Hz, 9H)
^{13}C NMR (101 MHz, $CDCl_3$) δ_C 147.6, 134.0, 126.6, 123.1, 52.2, 27.6, 24.9, 24.0, 20.0, 13.5

LRMS m/z (ESI-) 256.10 ($[M\text{-}Bu_3NH]^-$, 100%)

HRMS m/z (ESI-) $C_{12}H_{18}NSO_3$ requires 256.1007, found 256.1009 ($[M\text{-}Bu_3NH]^-$)

Tributylammonium 2-ethyl-6-methylphenylsulfamate (**2f**)

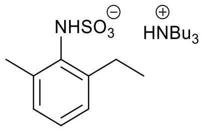

Following **general procedure 1:** 2-ethyl-6-methylaniline (0.14 mL, 1.0 mmol) and TBSAB (0.531 g, 2.0 mmol) were dissolved in anhydrous MeCN (2.0 mL) and heated at 30 °C for 1 h. The crude compound was extracted from cold water (10 mL) with EtOAc (4 × 50 mL) and washed with brine (10 mL). The organic layer was dried ($MgSO_4$), filtered, and evaporated *in vacuo*. The crude product was purified with (SiO_2; CH_2Cl_2/MeOH, 9.5:0.5, R_f = 0.2) to afford the title compound as a clear oil (386 mg, 97 %)

^{1}H NMR (400 MHz, $CDCl_3$) δ_H 9.79 (s, 1H), 7.04 – 6.88 (m, 3H), 2.95 (q, *J* = 7.6 Hz, 2H), 2.72 (m, 6H), 2.47 (s, 3H), 1.53 – 1.44 (m, 6H), 1.30 – 1.20 (m, *J* = 7.4 Hz, 6H), 1.15 (t, *J* = 7.6 Hz, 3H), 0.89 (t, *J* = 7.4 Hz, 9H)

^{13}C NMR (101 MHz, $CDCl_3$) δ_C 142.9, 137.2, 137.1, 128.4, 126.5, 126.5, 52.5, 25.3, 24.8, 20.2, 19.4, 15.1, 13.8

LRMS m/z (ESI-) 214.17 ($[M\text{-}Bu_3NH]^-$, 100%)

HRMS m/z (ESI-) $C_9H_{12}NO_3S$ requires 214.1711, found 214.1712 ($[M\text{-}Bu_3NH]^-$)

Tributylammonium 4-aminobenzenesulfonate (**4a**)

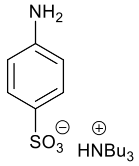

Following **general procedure 2**: Tributylammonium phenylsulfamate tributylammonium salt (600 mg, 1 mmol) was dissolved in dioxane (2.0 mL) and heated under reflux at 101 °C for 48 h. The flask was cooled and the solvent removed *in vacuo*. The crude reaction product was purified (SiO_2; CH_2Cl_2/MeOH, 8.5:1.5, R_f = 0.28) to yield the title compound as a clear oil (19 mg, 3%).

^{1}H NMR (400 MHz, MeOD) δ_H 7.57 (d, *J* = 8.5 Hz, 2H), 6.71 (d, *J* = 8.5 Hz, 2H), 3.15 - 3.07 (m, 6H), 1.67 (ddt, *J* = 13.4, 8.2, 6.2 Hz, 6H), 1.41 (h, *J* = 7.4 Hz, 6H), 1.00 (t, *J* = 7.4 Hz, 9H)

^{13}C NMR (101 MHz, MeOD) δ_C 150.0, 134.8, 126.2, 114.4, 51.7, 26.5, 20.3, 12.5.

LRMS m/z (ESI-) 172.01 ([M-$Bu_3NH]^-$, 100%)
HRMS m/z (ESI-) $C_6H_6NO_3S$ requires 172.0181, found 172.0179 ([M-$Bu_3NH]^-$)

Tributylammonium 3,5-dimethyl-4-aminobenzenesulfonate (**4b**)

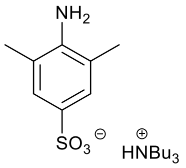

Following **general procedure 2**: 2,6-dimethylphenylsulfamate tributylammonium salt (500 mg, 1 mmol) was dissolved in dioxane (2 mL) and heated at 101 °C under reflux for 6 h. The crude product was purified firstly with (SiO_2; CH_2Cl_2/MeOH, 9:1, R_f = 0.23), and then (C_{18} silica gel; water/MeCN, 1:9) to yield the title compound as a yellow soild (122.1 mg, 24%).

M.P. 125-127 °C
^{1}H NMR (400 MHz, MeOD) δ_H 7.26 (t, *J* = 0.7 Hz, 2H), 3.04 – 2.95 (m, 6H), 2.08 (s, 6H), 1.62-1.50 (m, 6H), 1.30 (h, *J* = 7.4 Hz, 6H), 0.89 (t, *J* = 7.4 Hz, 9H)
^{13}C NMR (101 MHz, MeOD) δ_C 147.0, 134.2, 127.0, 122.0, 54.1, 27.0, 21.0, 17.9, 13.9
LRMS m/z (ESI-) 200.14 ([M-$Bu_3NH]^-$, 100%)
HRMS m/z (ESI-) $C_8H_{10}NSO_3$ requires 200.1440, found 200.1436 ([M-$Bu_3NH]^-$)

Tributylammonium 3,5-diethyl-4-aminobenzenesulfonate (**4d**)

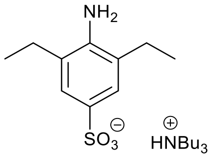

Following **general procedure 2**: 2,6-diethylphenylsulfamate tributylammonium salt (0.80 mL, 1 mmol) was dissolved in dioxane (2 mL) and heated at 101 °C under reflux for 8 h. The crude product was purified with (SiO_2; CH_2Cl_2/MeOH, 9:1, R_f = 0.23) to yield the title compound as a clear yellow oil (312 mg, 15%).

^{1}H NMR (400 MHz, MeOD) δ_H 7.42 (s, 2H), 3.12 – 3.08 (m, 6H), 2.57 (q, *J* = 7.5 Hz, 4H), 1.66 (m, 6H), 1.44 – 1.37 (m, *J* = 7.4 Hz, 6H), 1.25 (t, *J* = 7.5 Hz, 6H), 0.99 (t, *J* = 7.4 Hz, 9H)
^{13}C NMR (101 MHz, MeOD) δ_C 145.7, 134.4, 127.9, 124.8, 53.9, 26.8, 25.1, 20.9, 13.9, 13.3
LRMS m/z (ESI-) 228.07 ([M-$Bu_3NH]^-$, 100%)
HRMS m/z (ESI-) $C_{10}H_{14}NSO_3$ requires 228.0710, found 228.0711 ([M-$Bu_3NH]^-$)

Tributylammonium 3,5-diisopropyl-4-aminobenzenesulfonate (**4e**)

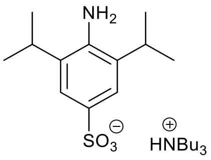

Following **general procedure 2**: 2,6-diisopropylphenylsulfamate tributylammonium salt (0.76 mL, 1 mmol) was dissolved in dioxane (2 mL) and heated at 101 °C under reflux for 8 h. The crude product was purified with (SiO_2; CH_2Cl_2/MeOH, 8:2, R_f = 0.2) to yield the title compound as a clear yellow oil (271 mg, 15%).

^{1}H NMR (400 MHz, MeOD) δ_H 7.49 (s, 2H), 5.50 (s, 1H), 3.15 – 3.10 (m, 6H), 3.02 (q, *J* = 6.8 Hz, 2H), 1.73 – 1.64 (m, 6H), 1.42 (m, *J* = 7.4 Hz, 6H), 1.26 (d, *J* = 6.8 Hz, 12H), 1.00 (t, *J* = 7.4 Hz, 9H)
^{13}C NMR (101 MHz, MeOD) δ_C 144.7, 134.7, 132.9, 121.9, 54.2, 28.9, 27.0, 23.0, 21.1, 14.1
LRMS m/z (ESI-) 256.10 ($[M\text{-}Bu_3NH]^-$, 100%)
HRMS m/z (ESI-) $C_{12}H_{18}NSO_3$ requires 256.1008, found 256.1011 ($[M\text{-}Bu_3NH]^-$)

Following **general procedure 3**: 2,6-diisopropylaniline (0.76 mL, 4 mmol) and TBSAB (2.124 g, 8 mmol) were dissolved in DMF (2 mL) and heated at 120 °C under reflux for 8.5 h. The crude product was purified with (SiO_2; CH_2Cl_2/MeOH, 9.4:0.6, R_f = 0.2) to yield the title compound as a clear yellow oil (646 mg, 44%).

^{1}H NMR (400 MHz, MeOD) δ_H 7.49 (s, 2H), 3.16 – 3.04 (m, 6H), 3.05 (p, *J* = 6.8 Hz, 2H), 1.74 – 1.61 (m, 6H), 1.40 (m, *J* = 7.4 Hz, 6H), 1.25 (d, *J* = 6.8 Hz, 12H), 0.99 (t, *J* = 7.4 Hz, 9H)
^{13}C NMR (101 MHz, MeOD) δ_C 144.7, 134.7, 132.9, 121.9, 54.2, 28.9, 27.0, 23.0, 21.1, 14.1
LRMS m/z (ESI-) 256.10 ($[M\text{-}Bu_3NH]^-$, 100%)
HRMS m/z (ESI-) $C_{12}H_{18}NSO_3$ requires 256.1011, found 256.1012 ($[M\text{-}Bu_3NH]^-$)

Tributylammonium 3-ethyl-5-methyl-4-aminobenzenesulfonate (**4f**)

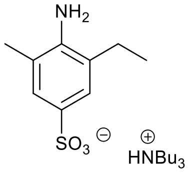

Following **general procedure 2**: The 2-ethyl-6-methylphenylsulfamate tributylammonium salt (0.56 mL, 1 mmol) was dissolved in dioxane (2 mL) and heated at 101 °C for 24 h. The crude product was firstly purified with (SiO_2; *n*-hexane/Ethyl acetate, 9.5:0.5, R_f = 0.2) and then purified with (SiO_2; CH_2Cl_2/MeOH, 9.5:0.5, R_f = 0.2) to give a total yield (82.5 mg, 5%). The purity was confirmed by ^{1}H NMR (14 % title compound; remainder 86 % is TBSAB)
Following **general procedure 3**: The 2-ethyl-6-methylaniline (560 mg, 4 mmol) and TBSAB

(2.124 g, 8 mmol) were dissolved in DMF (2 mL) and heated at 120 °C under reflux for 8.5 h. The crude product was purified with (SiO_2; CH_2Cl_2/MeOH, 9.4:0.6, R_f = 0.2) to yield the title compound as a clear oil (768 mg, 40%).

^{1}H NMR (400 MHz, MeOD) δ_H 7.41 – 7.39 (m, 1H), 7.38 – 7.36 (m, 1H), 3.14 – 3.05 (m, 6H), 2.57 (q, *J* = 7.5 Hz, 2H), 2.19 (s, 3H), 1.73 – 1.60 (m, 6H), 1.40 (h, *J* = 7.4 Hz, 6H), 1.24 (t, *J* = 7.5 Hz, 3H), 0.99 (t, *J* = 7.4 Hz, 9H).
^{13}C NMR (101 MHz, MeOD) δ_C 146.0, 134.6, 128.0, 126.8, 125.1, 122.5, 54.0, 26.8, 25.1, 20.9, 18.0, 13.9, 13.4
LRMS m/z (ESI-) 214.17 ([M-Bu_3NH]$^-$, 100%)
HRMS m/z (ESI-) $C_9H_{12}NO_3S$ requires 214.1699, found 214.1701 ([M-Bu_3NH]$^-$)

Tributylammonium 3,5-dichloro-4-aminobenzenesulfonate (**4g**)

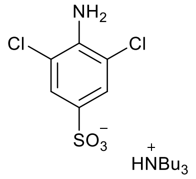

Following **general procedure 3**: 2,6-dichloroaniline (640 mg, 4 mmol) and TBSAB (2.124 g, 8 mmol) were dissolved in DMF (2 mL) and heated at 120 °C under reflux for 8.5 h. The crude product was purified with (SiO_2; CH_2Cl_2/MeOH, 9.4:0.6, R_f = 0.2) to yield the title compound as a clear brown oil (85 mg, 5%).

^{1}H NMR (400 MHz, MeOD) δ_H 7.60 (s, 2H), 3.15 – 3.08 (m, 6H), 1.73 – 1.64 (m, 6H), 1.42 (m, *J* = 7.4 Hz, 6H), 1.00 (t, *J* = 7.4 Hz, 9H)
^{13}C NMR (101 MHz, MeOD) δ_C 144.0, 135.3, 126.8, 119.0, 54.0, 26.9, 20.9, 13.9
LRMS m/z (ESI-) 241.07 ([M^{35}Cl - Bu_3NH]$^-$, 100%), 243.07 ([M^{37}Cl - Bu_3NH]$^-$, 40%)
HRMS m/z (ESI-) $C_6H_4Cl_2NO_3S$ requires 241.0742, found 241.0744 ([M^{35}Cl - Bu_3NH]$^-$)

Tributylammonium 2,3-dichloro-4-aminobenzenesulfonate (**4h**)

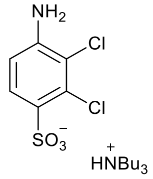

Following **general procedure 3**: 2,3-dichloroaniline (0.48 mL, 4 mmol) and TBSAB (2.124 g, 8 mmol) were dissolved in DMF (2 mL) and heated at 120 °C under reflux for 8.5 h. The

product was purified with (SiO2; CH_2Cl_2/MeOH, 9:1, Rf = 0.23) to yield the title compound as a clear oil (111 mg, 7%).

^{1}H NMR (400 MHz, MeOD) δ_H 7.70 (d, *J* = 8.7 Hz, 1H), 6.72 (d, *J* = 8.7 Hz, 1H), 3.15 – 3.08 (m, 6H), 1.72 – 1.63 (m, 6H), 1.41 (t, *J* = 7.4 Hz, 6H), 1.00 (t, *J* = 7.4 Hz, 9H).
^{13}C NMR (101 MHz, MeOD) δ_C 148.9, 132.6, 131.7, 128.8, 118.4, 112.2, 53.8, 26.6, 20.7, 13.7.
LRMS m/z (ESI-) 241.06 ([M^{35}Cl - $Bu_3NH]^-$, 100%), 243.06 ([M^{37}Cl - $Bu_3NH]^-$, 40%)
HRMS m/z (ESI-) $C_6H_4Cl_2NO_3S$ requires 241.0640, found 241.0639 ([M^{35}Cl - $Bu_3NH]^-$)

Tributylammonium 3-methyl-5-nitro-4-aminobenzenesulfonate (**4i**)

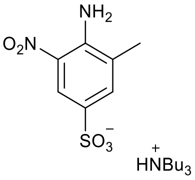

2-methyl-6-nitroaniline (681 mg, 4 mmol) and TBSAB (2.124 g, 8 mmol) were dissolved in DMF (2 mL) and heated at 120 °C under reflux for 8.5 h. The product was purified with (SiO2; CH2Cl2/MeOH, 9.4:0.6, R_f = 0.2) to yield the title compound as yellow oil (1008 mg, 60%).

^{1}H NMR (400 MHz, MeOD) δ_H 8.43 (d, *J* = 2.0 Hz, 1H), 7.70 (d, *J* = 2.0 Hz, 1H), 3.17 – 3.08 (m, 6H), 2.29 (s, 3H), 1.74 – 1.62 (m, 6H), 1.40 (h, *J* = 7.3 Hz, 6H), 0.99 (t, *J* = 7.3 Hz, 9H)
^{13}C NMR (101 MHz, MeOD) δ_C 145.4, 132.5, 131.9, 129.8, 126.4, 121.5, 52.6, 25.4, 19.50, 16.6, 12.5.
LRMS m/z (ESI-) 231.20 ([M-$Bu_3NH]^-$, 100%)
HRMS m/z (ESI-) $C_7H_7N_2SO_5$ requires 231.2008, found 231.2007 ([M-$Bu_3NH]^-$)

Tributylammonium *N,N*-dimethyl-4-aminobenzenesulfonate (**4j**)

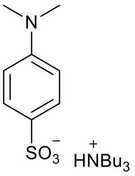

N,N-dimethylaniline (0.52 mL, 4 mmol) and TBSAB (2.124 g, 8 mmol) were dissolved in DMF (2 mL) and heated at 120 °C under reflux for 2 h. The product was purified with (SiO_2; CH_2Cl_2/MeOH, 9.6:0.4, R_f = 0.2) to yield the title compound as white solid (1.08 g, 70%).

M.P. 78-79 °C
^{1}H NMR (400 MHz, MeOD) δ_H 7.69 – 7.61 (m, 2H), 6.76 – 6.68 (m, 2H), 3.14 – 3.05 (m, 6H), 2.99(s, 6H), 1.73 – 1.60 (m, 6H), 1.40 (h, *J* = 7.4 Hz, 6H), 0.99 (t, *J* = 7.4 Hz, 9H).

^{13}C NMR (101 MHz, MeOD) δ_C 153.2, 133.4, 128.2, 112.1, 54.0, 40.4, 26.9, 20.9, 13.9
LRMS m/z (ESI-) 200.14 ($[M-Bu_3NH]^-$, 100%)
HRMS m/z (ESI-) $C_8H_{10}NSO_3$ requires 200.1440, found 200.1442 ($[M-Bu_3NH]^-$)

Tributylammonium 4-hydroxy-3,5-diisopropylbenzenesulfonate (**5**)

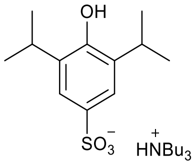

N,*N*-dimethylaniline (0.52 mL, 4 mmol) and TBSAB (2.124 g, 8 mmol) were dissolved in DMF (2 mL) and heated at 120 °C under reflux for 2 h. The product was purified with (SiO2; CH_2Cl_2/MeOH, 9.6:0.4, R_f = 0.2) to yield the title compound as white solid (1.08 g, 70%).

M.P. 43-45 °C
^{1}H NMR (400 MHz, MeOD) δ_H 7.10 (s, 2H), 3.79 (q, *J* = 6.9 Hz, 2H), 3.16 – 3.07 (m, 6H), 1.73 – 1.61 (m, 6H), 1.41 (h, *J* = 7.4 Hz, 6H), 1.19(d, *J* = 6.9 Hz, 12H), 1.00 (t, *J* = 7.4 Hz, 9H).
^{13}C NMR (101 MHz, MeOD) δ_C 148.5, 144.3, 126.7, 124.8, 54.1, 27.9, 26.9, 24.2, 20.9, 13.9
LRMS m/z (ESI-) 257.32 ($[M-Bu_3NH]^-$, 100%)
HRMS m/z (ESI-) $C_{12}H_{17}O_4S$ requires 257.3211, found 257.3210 ($[M-Bu_3NH]^-$)

Tributylammonium thiophene-2-sulfonate (**6**)

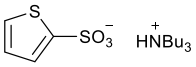

Thiophene (0.32 mL, 4 mmol) and TBSAB (2.124 g, 8 mmol) were dissolved in DMF (2 mL) and heated at 120 °C under reflux for 24 h. The product was purified with (SiO_2; CH_2Cl_2/MeOH, 9.6:0.4, R_f = 0.2) to yield the title compound as red oil (0.908 g, 65%).

^{1}H NMR (400 MHz, MeOD) δ_H 7.49 (dd, *J* = 5.0, 1.3 Hz, 1H), 7.42 (dd, *J* = 3.6, 1.3 Hz, 1H), 7.00 (dd, *J* = 5.0, 3.6 Hz, 1H), 3.17 – 3.09 (m, 6H), 1.75 – 1.62 (m, 6H), 1.42 (h, *J* = 7.4 Hz, 6H), 1.00 (t, *J* = 7.4 Hz, 9H).
^{13}C NMR (101 MHz, MeOD) δ_C 148.4, 128.9, 128.9, 127.4, 54.1, 26.9, 20.9, 13.9
LRMS m/z (ESI-) 163.19 ($[M-Bu_3NH]^-$, 100%)
HRMS m/z (ESI-) $C_4H_3O_3S_2$ requires 163.1922, found 163.1920 ($[M-Bu_3NH]^-$)

Tributylammonium 1-methyl-1H-pyrrole-2-sulfonate (**7**)

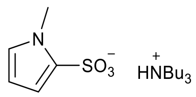

N-Methylpyrrole (0.36 mL, 4 mmol) and TBSAB (2.124 g, 8 mmol) were dissolved in DMF (2 mL) and heated at 120 °C under reflux for 24 h. The product was purified with (SiO_2; CH_2Cl_2/MeOH, 9.2:0.8, R_f = 0.2) to yield the title compound as white solid (0.706 g, 51%).

M.P. 76–79 °C
^{1}H NMR (400 MHz, MeOD) δ_H 6.70 (dd, *J* = 2.6, 1.9 Hz, 1H), 6.47 (dd, *J* = 3.7, 1.9 Hz, 1H), 5.95 (dd, *J* = 3.7, 2.6 Hz, 1H), 3.84 (s, 3H), 3.16 – 3.05 (m, 6H), 1.73 – 1.61 (m, 6H), 1.41 (h, *J* = 7.4 Hz, 6H), 1.00 (t, *J* = 7.4 Hz, 9H).
^{13}C NMR (101 MHz, MeOD) δ_C 134.9, 126.2, 112.2, 106.8, 54.1, 35.4, 26.9, 20.9, 13.9
LRMS m/z (ESI-) 160.17 ([M-Bu_3NH]$^-$, 100%)
HRMS m/z (ESI-) $C_5H_6NO_3S$ requires 160.1712, found 160.1713 ([M-Bu_3NH]$^-$)

Tributylammonium 1H-pyrrole-2-sulfonate (**8**)

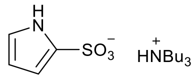

Pyrrole (0.28 mL, 4 mmol) and TBSAB (2.124 g, 8 mmol) were dissolved in DMF (2 mL) and heated at 120 °C under reflux for 2 h. The crude was purified with (SiO_2; CH_2Cl_2/MeOH, 9.2:0.8, R_f = 0.2) to yield the title compound as solid (0.678 g, 60%).

M.P. 103-104 °C
^{1}H NMR (400 MHz, MeOD) δ_H 6.75 (dd, *J* = 2.6, 1.9 Hz, 1H), 6.45 (dd, *J* = 3.6, 1.9 Hz, 1H), 6.06 (dd, *J* = 3.6, 2.6 Hz, 1H), 3.15 – 3.06 (m, 6H), 1.73 – 1.61 (m, 6H), 1.43 (h, *J* = 7.4 Hz, 6H), 1.00 (t, *J* = 7.4 Hz, 9H).
^{13}C NMR (101 MHz, MeOD) δ_C 132.8, 118.7, 108.5, 107.1, 52.6, 25.4, 19.5, 12.5
LRMS m/z (ESI-) 146.14 ([M-Bu_3NH]$^-$, 100%)
HRMS m/z (ESI-) $C_4H_4NO_3S$ requires 146.1486, found 146.1488 ([M-Bu_3NH]$^-$)
Tributylammonium 4-ethyl-3,5-dimethyl-1*H*-pyrrole-2-sulfonate (**9**)

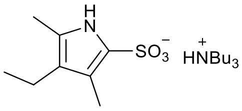

Pyrrole (0.28 mL, 4 mmol) and TBSAB (2.124 g, 8 mmol) were dissolved in DMF (2 mL) and heated at 120 °C under reflux for 2 h. The crude was purified with (SiO_2; CH_2Cl_2/MeOH, 9.4:0.6, R_f = 0.2) to yield the title compound as purple solid (1220 mg, 79%).

M.P. 155-161 °C

¹H NMR (400 MHz, MeOD) δ $_H$ 3.13 – 3.07 (6 H, m), 2.35 (2 H, q, *J* = 9.7), 2.17 (3 H, s), 2.12 (3 H, s), 1.72 – 1.62 (6 H, m), 1.40 (6 H, h, *J* = 7.5), 1.00 (12 H, dt, *J* = 9.7, 7.5).

¹³C NMR (101 MHz, MeOD) δ$_C$ 125.7, 123.3, 121.0, 117.0, 52.5, 25.5, 19.6, 16.9, 14.6, 12.5, 9.3, 8.4

LRMS m/z (ESI-) 202.25 ([M-$Bu_3NH]^-$, 100%)

HRMS m/z (ESI-) $C_8H_{12}NO_3S$ requires 202.2510, found 202.2511 ([M-$Bu_3NH]^-$)

Tributylammonium 1-methyl-1H-indole-3-sulfonate (**10**)

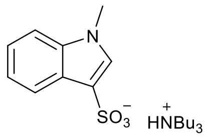

Following **general procedure 3**: *N*-Methylindole (0.52 mL, 4 mmol) and TBSAB (2.124 g, 8 mmol) were put in a 10 mL round bottom flask, followed by DMF (2 mL). The reaction was heated at 120 °C under reflux for 8.5 h. The crude product was purified with (SiO_2; CH_2Cl_2/MeOH, 9.4:0.6, R_f = 0.2) to yield the title compound as yellow solid (1280 mg, 80%).

M.P. 104-107 °C

¹H NMR (400 MHz, MeOD) δ$_H$ 7.93 (1 H, dt, *J* = 8.0, 1.1), 7.53 (1 H, s), 7.40 (1 H, dt, *J* = 8.3, 1.3), 7.24 (1 H, ddd, *J* = 8.3, 7.1, 1.3), 7.15 (1 H, ddd, *J* = 8.0, 7.1, 1.1), 3.81 (3 H, s), 3.09 – 3.05 (6 H, m), 1.68 – 1.60 (6 H, m), 1.38 (6 H, m, *J* = 7.4), 0.97 (9 H, t, *J* = 7.4).

¹³C NMR (101 MHz, MeOD) δ$_C$ 138.2, 137.0, 129.3, 124.6, 122.1, 120.0, 119.1, 109.3, 52.6, 31.7, 25.4, 19.5, 12.6.

LRMS m/z (ESI-) 210.02 ([M-$Bu_3NH]^-$, 100%)

HRMS m/z (ESI-) $C_9H_8NO_3S$ requires 210.0225, found 210.0228 ([M-$Bu_3NH]^-$)

Tributylammonium 4-amino-3-fluoro-5-(trifluoromethyl)benzenesulfonate (**11**)

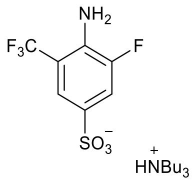

Following **general procedure 3**: 2-fluoro-6-(trifluoromethyl)aniline (0.52 mL, 4 mmol) and TBSAB (2.124 g, 8 mmol) were put in a 10 mL round bottom flask, followed by DMF (2 mL). The reaction was heated at 120 °C under reflux for 8.5 h. The crude product was purified with (SiO_2; CH_2Cl_2/MeOH, 9.4:0.6, R_f = 0.2) to yield the title compound as clear oil (1123mg, 46%).

¹H NMR (400 MHz, MeOD) δ$_H$ 7.68 (d, *J* = 2.0 Hz, 1H), 7.56 (d, *J* = 2.0 Hz, 1H), 3.17 – 3.09

(m, 6H), 1.75 – 1.62 (m, 6H), 1.42 (h, *J* = 7.4 Hz, 6H), 1.00 (t, *J* = 7.4 Hz, 9H).

^{13}C NMR (101 MHz, MeOD) δ_C 151.5, 149.1, 132.5 (d, $^{1}J_{C\text{-}F}$ = 220.3 Hz), 133.8, 125.8 (q, $^{2}J_{C\text{-}F}$ = 33.6 Hz), 123.5, 120.1 (q, $^{1}J_{C\text{-}F}$ = 273.9 Hz), 116.4 (d, $^{2}J_{C\text{-}F}$ = 35.5 Hz), 54.0, 26.8, 20.9, 13.9.

^{19}F NMR (377 MHz, MeOD) δ -64.53, -134.66

LRMS m/z (ESI-) 258.17 ($[M\text{-}Bu_3NH]^-$, 100%)

HRMS m/z (ESI-) $C_7H_4F_4NO_3S$ requires 210.0225, found 210.0228 ($[M\text{-}Bu_3NH]^-$)

Copies of ^{1}H, ^{13}C, ^{19}F NMR spectra

^{1}H NMR spectrum of **2a** (300 MHz, $CDCl_3$)

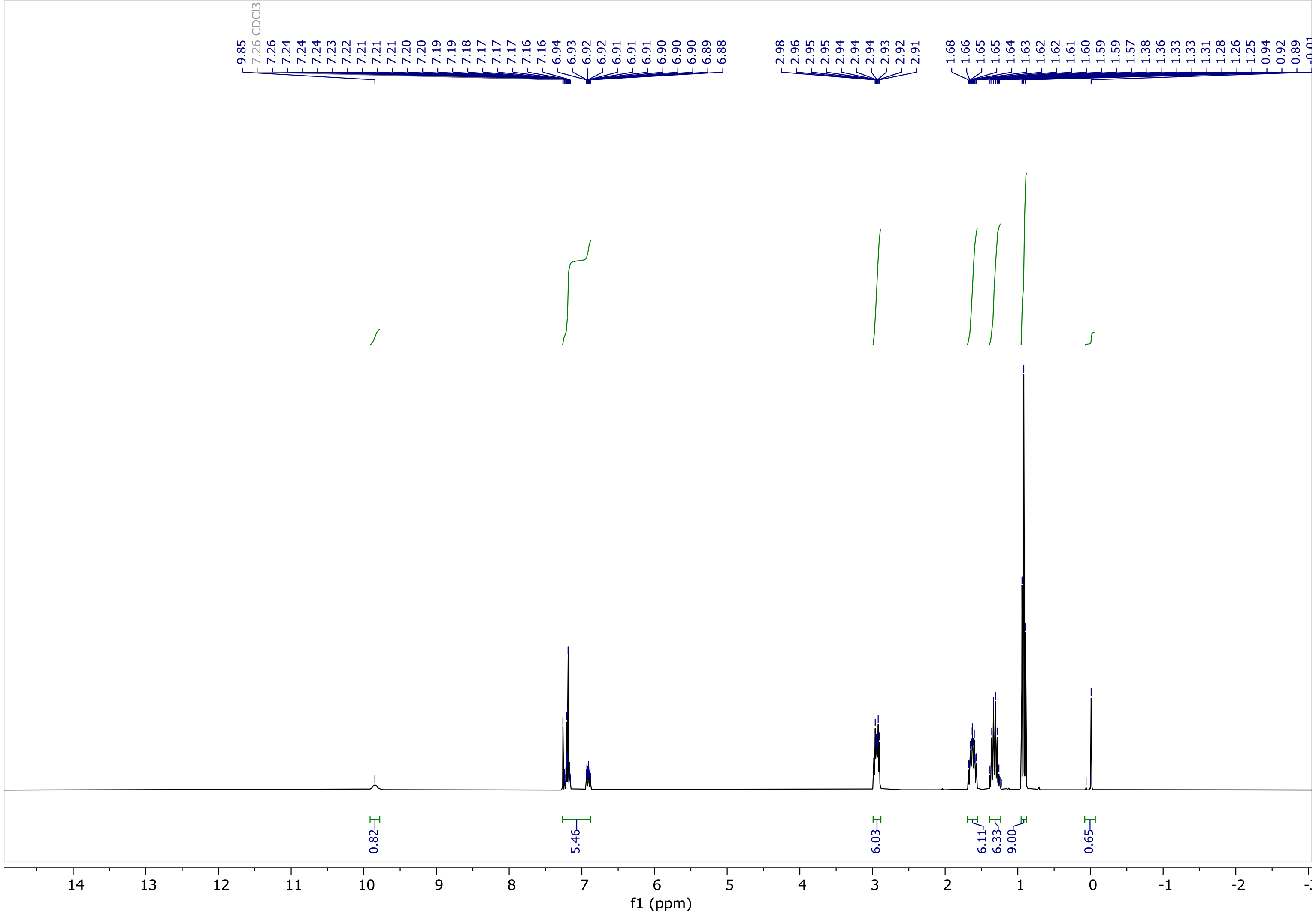

^{13}C NMR spectrum of **2a** (101 MHz, $CDCl_3$)

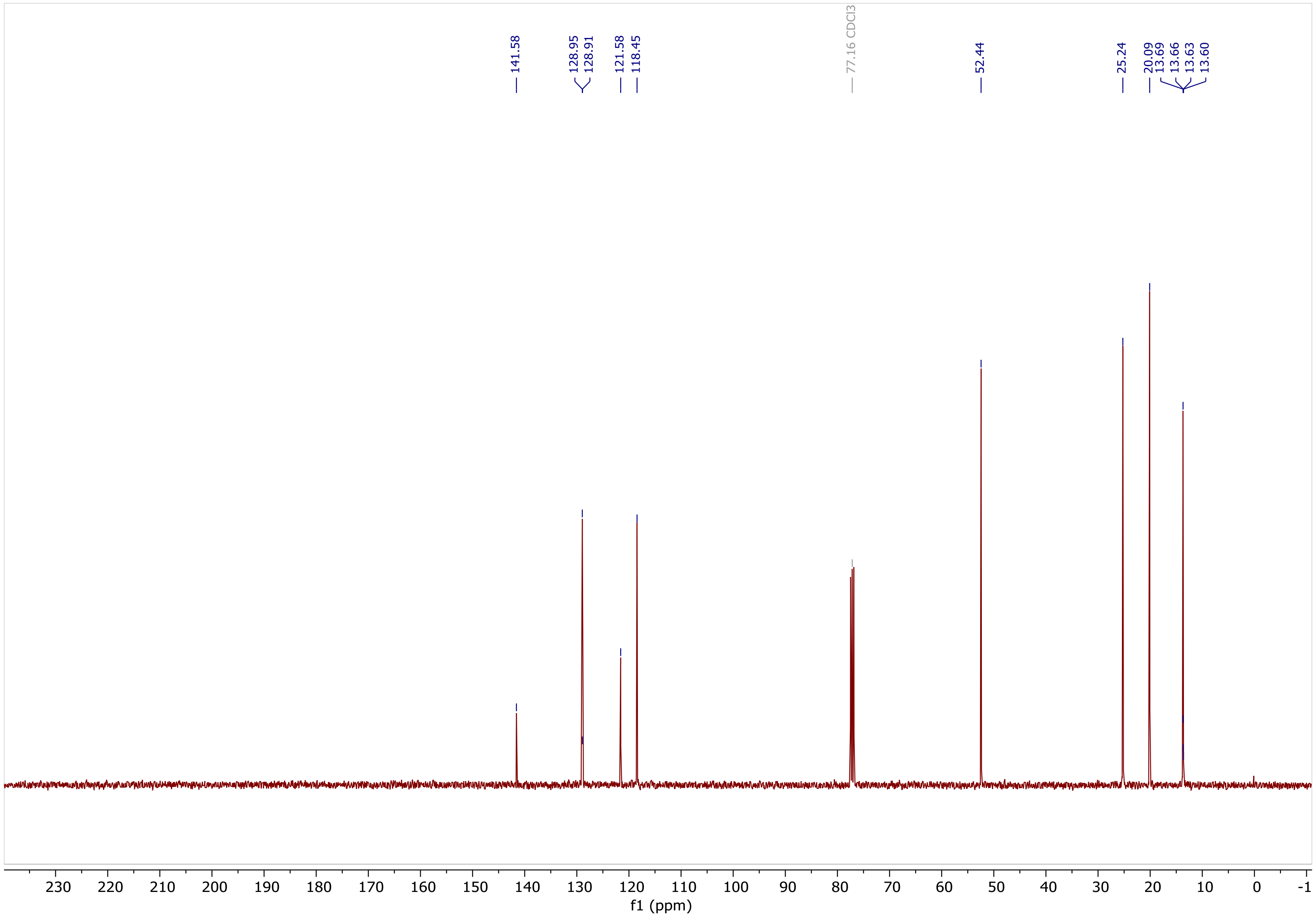

^{1}H NMR spectrum of **2b** (300 MHz, $CDCl_3$)

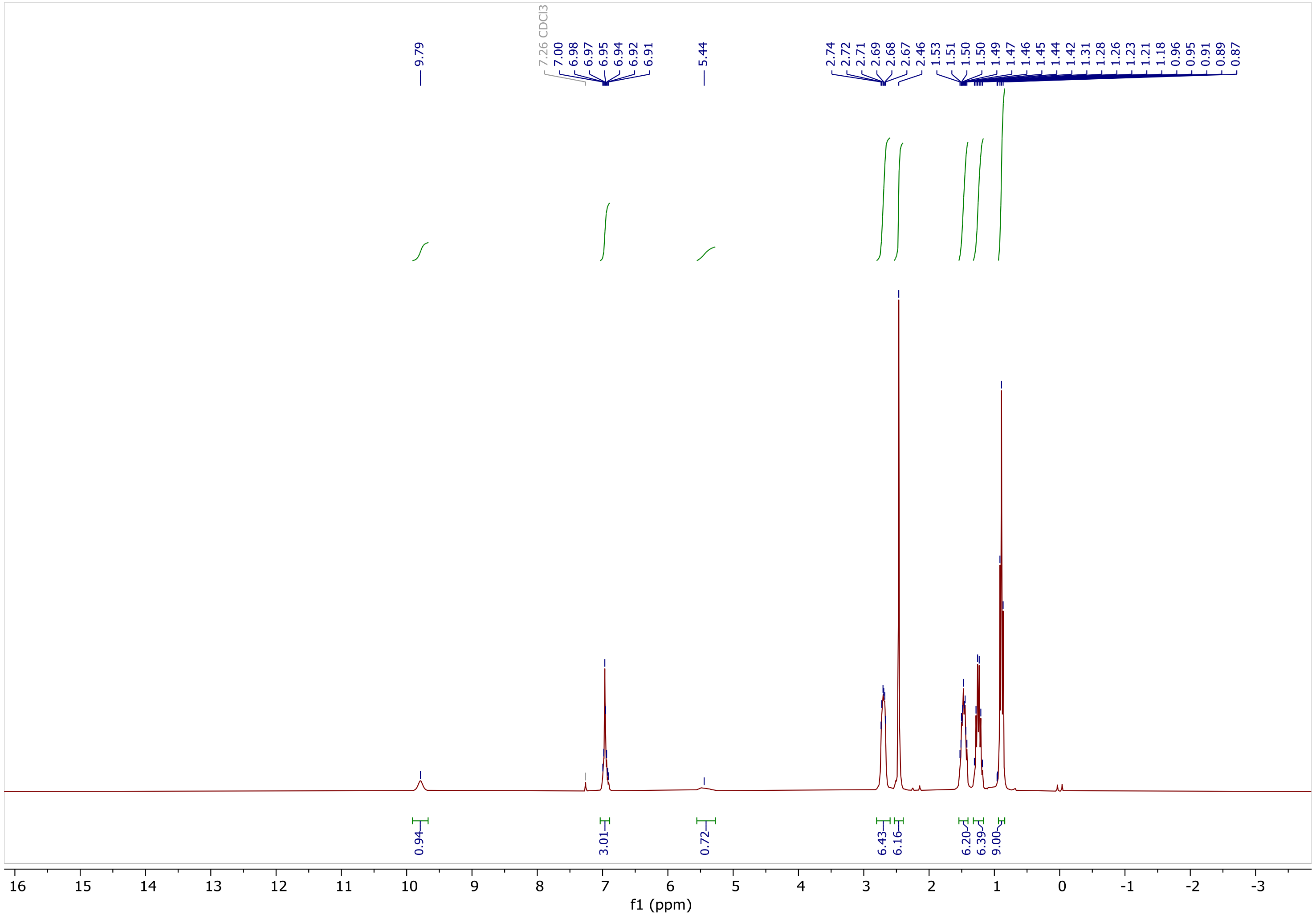

^{13}C NMR spectrum of **2b** (101 MHz, $CDCl_3$)

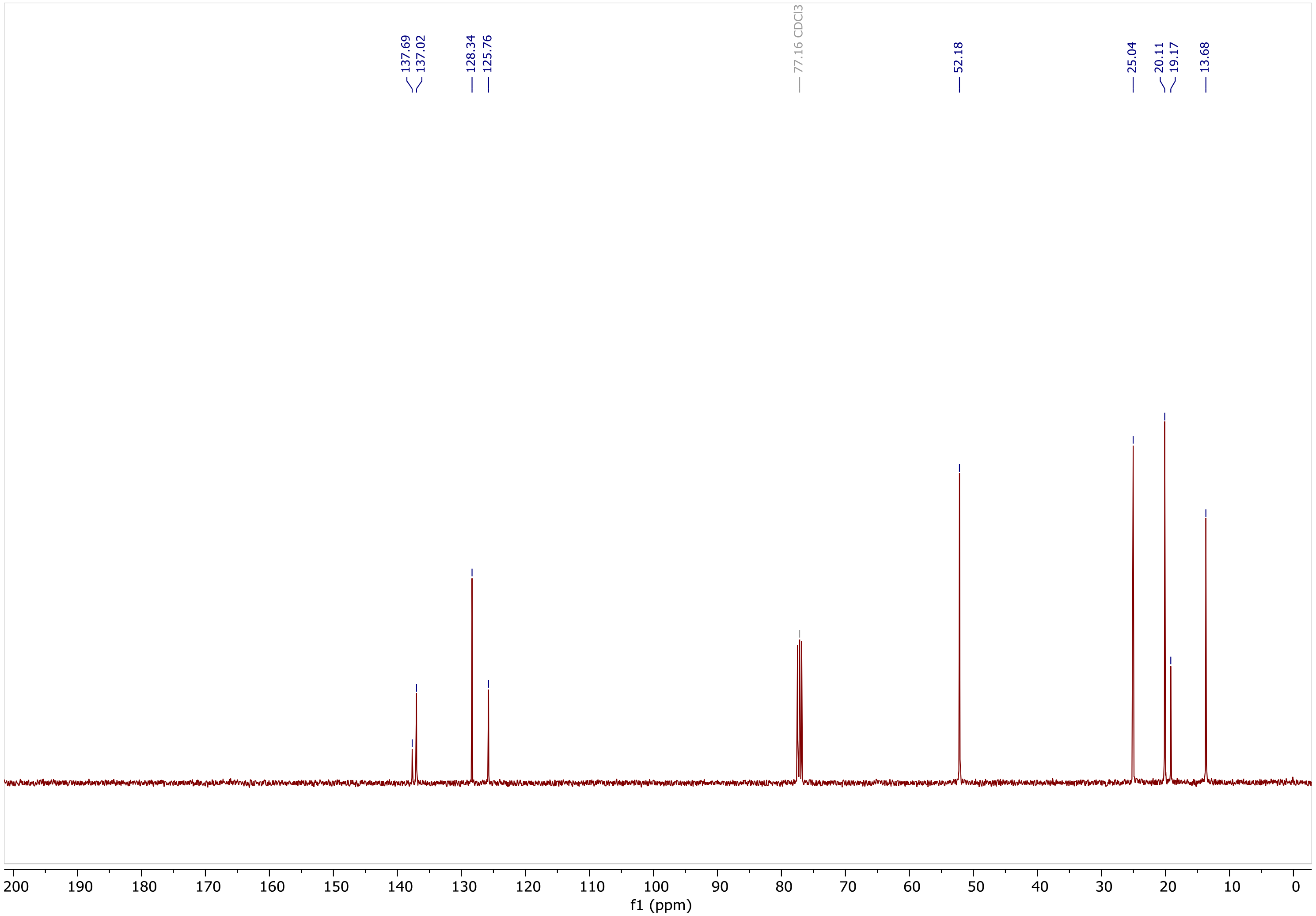

^{1}H NMR spectrum of **2c** (400 MHz, $CDCl_3$)

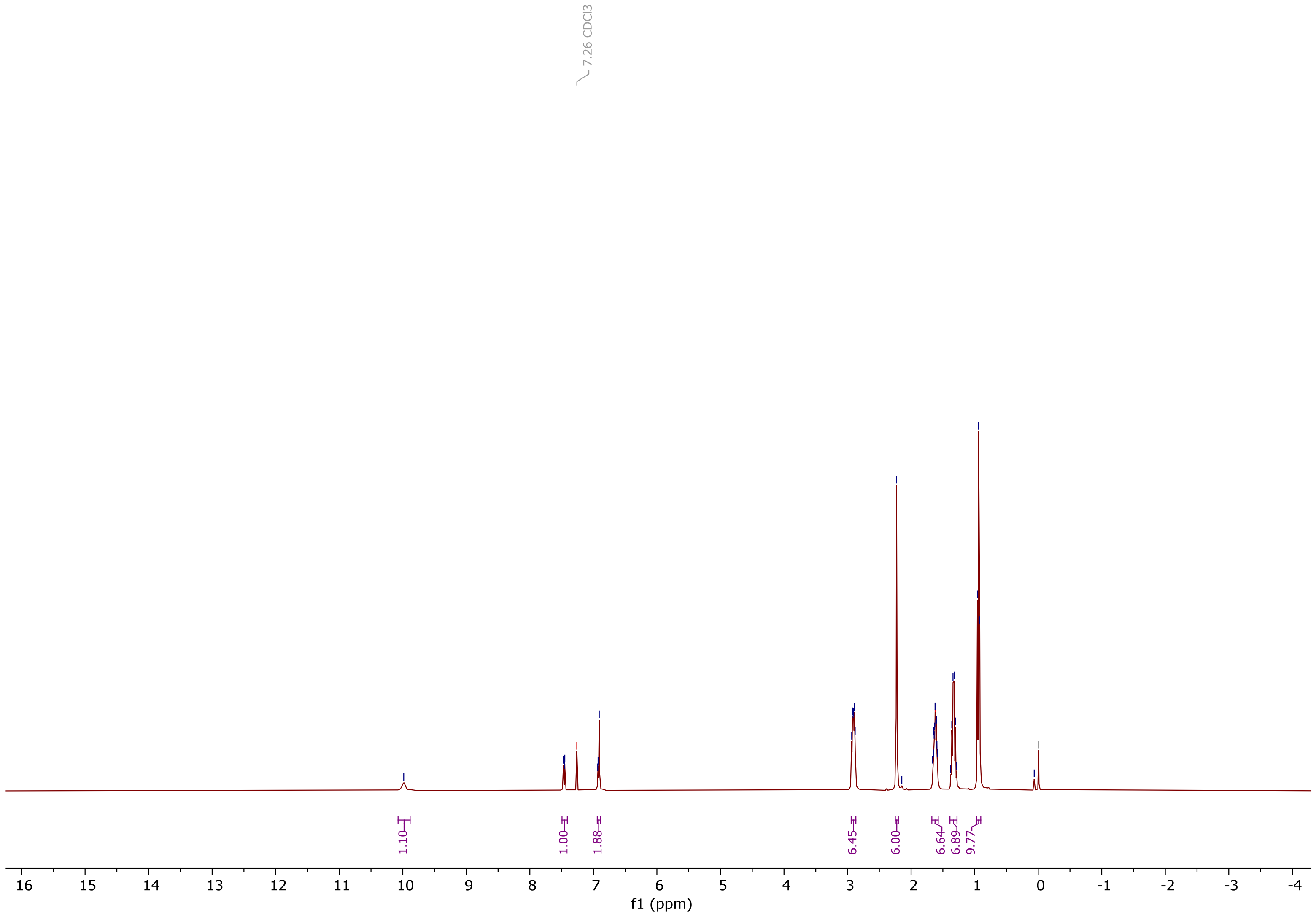

^{13}C NMR spectrum of **2c** (400 MHz, $CDCl_3$)

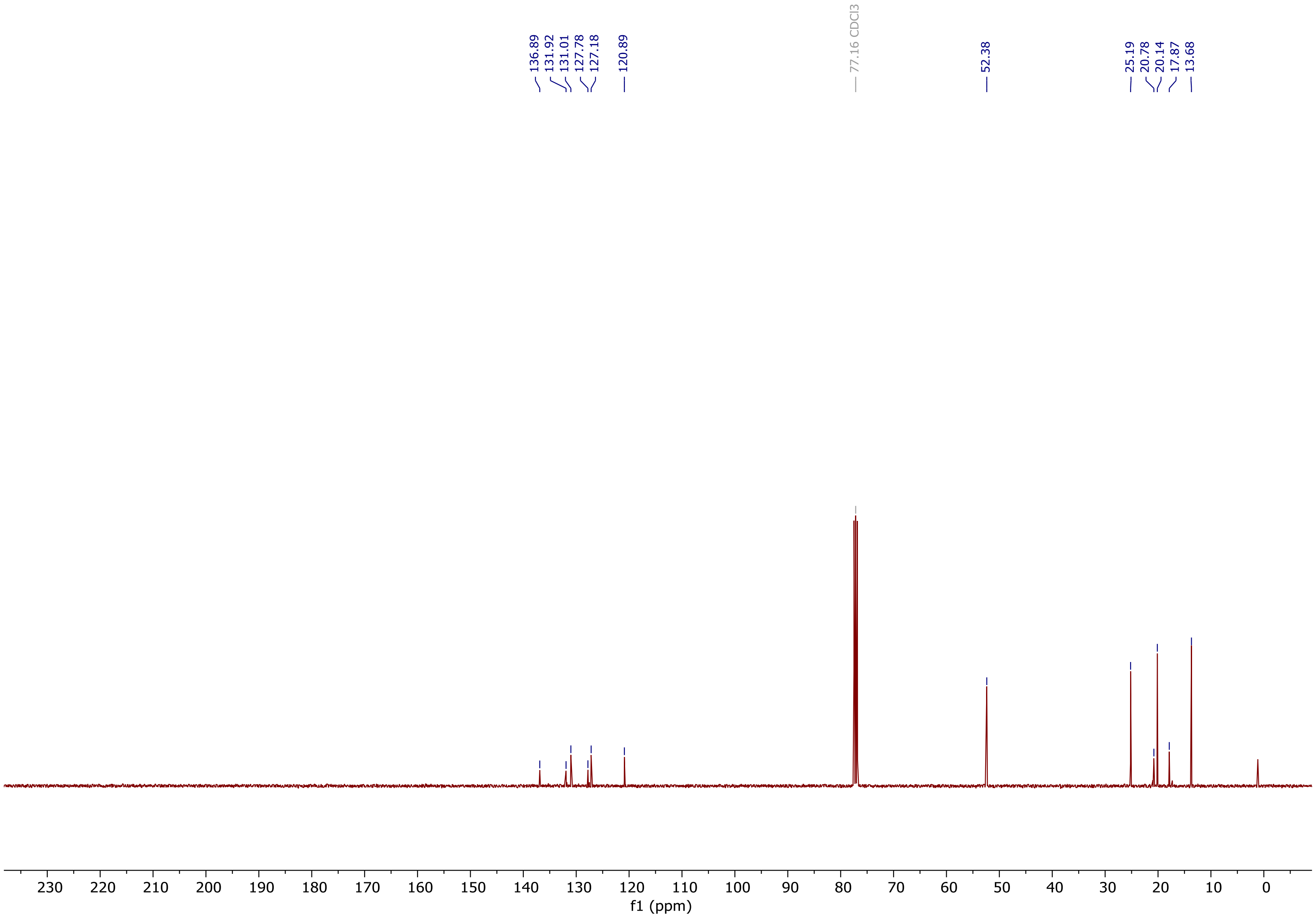

^{1}H NMR spectrum of **2d** (400 MHz, $CDCl_3$)

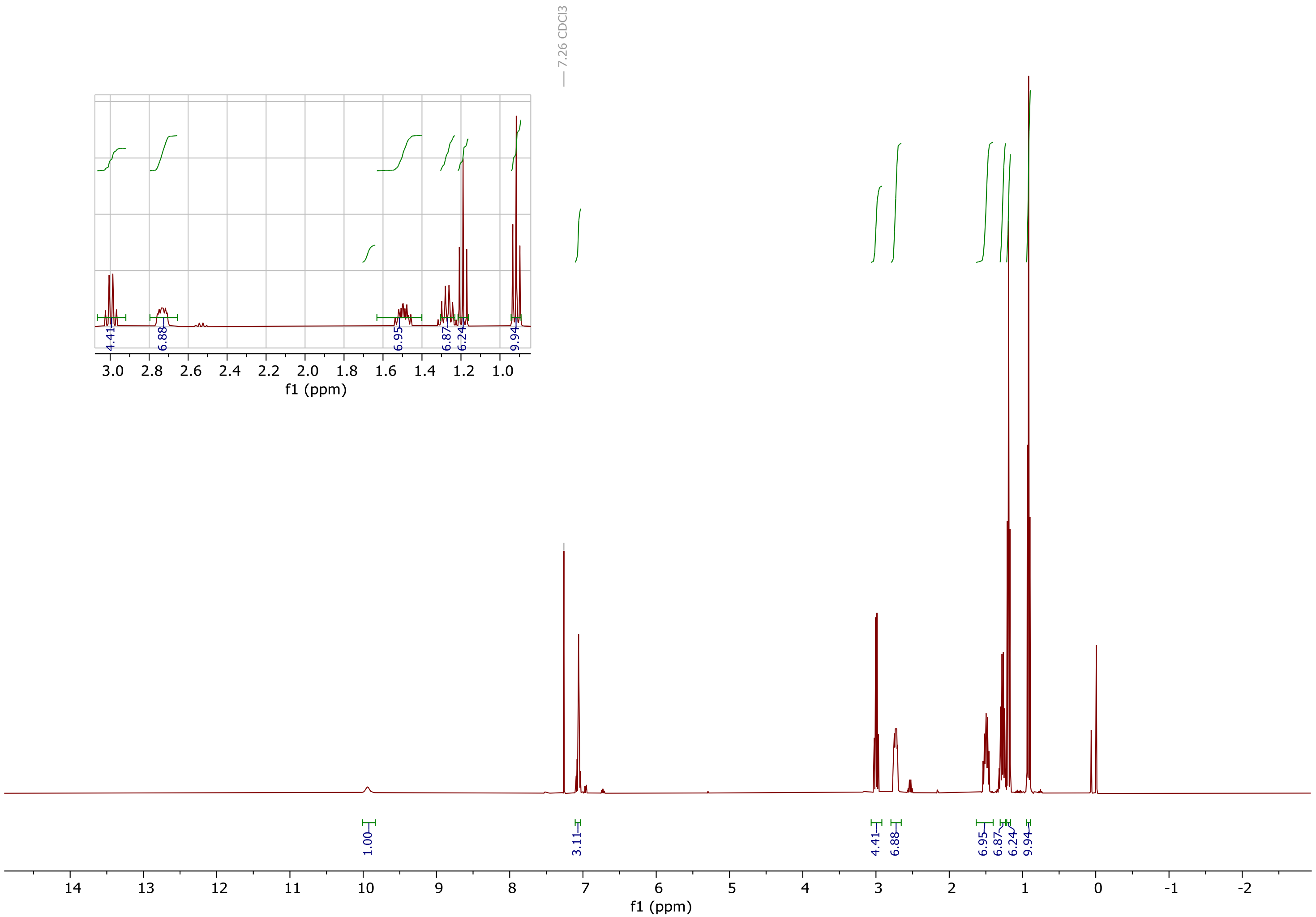

^{13}C NMR spectrum of **2d** (101 MHz, $CDCl_3$)

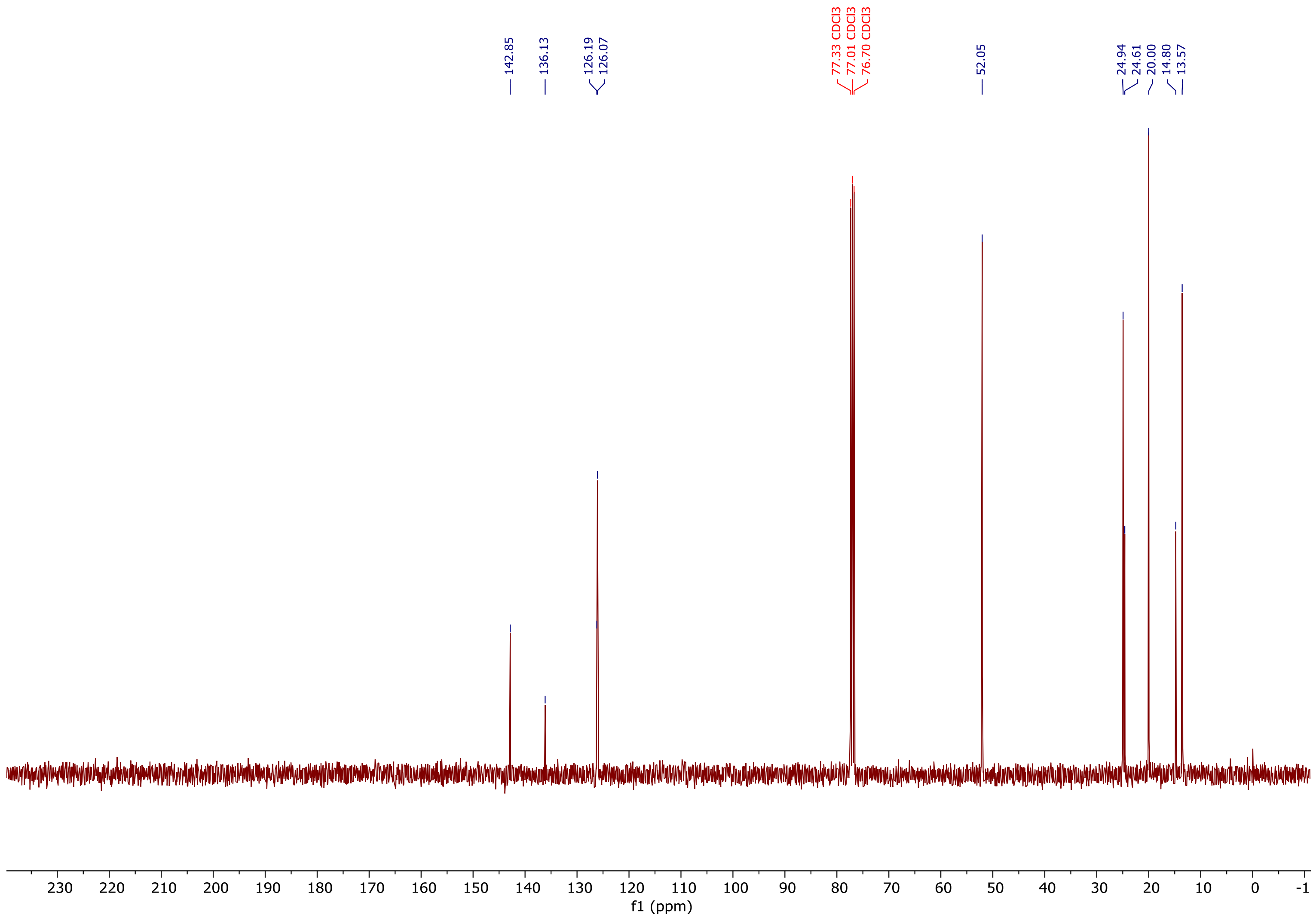

^{1}H NMR spectrum of **2e** (400 MHz, $CDCl_3$)

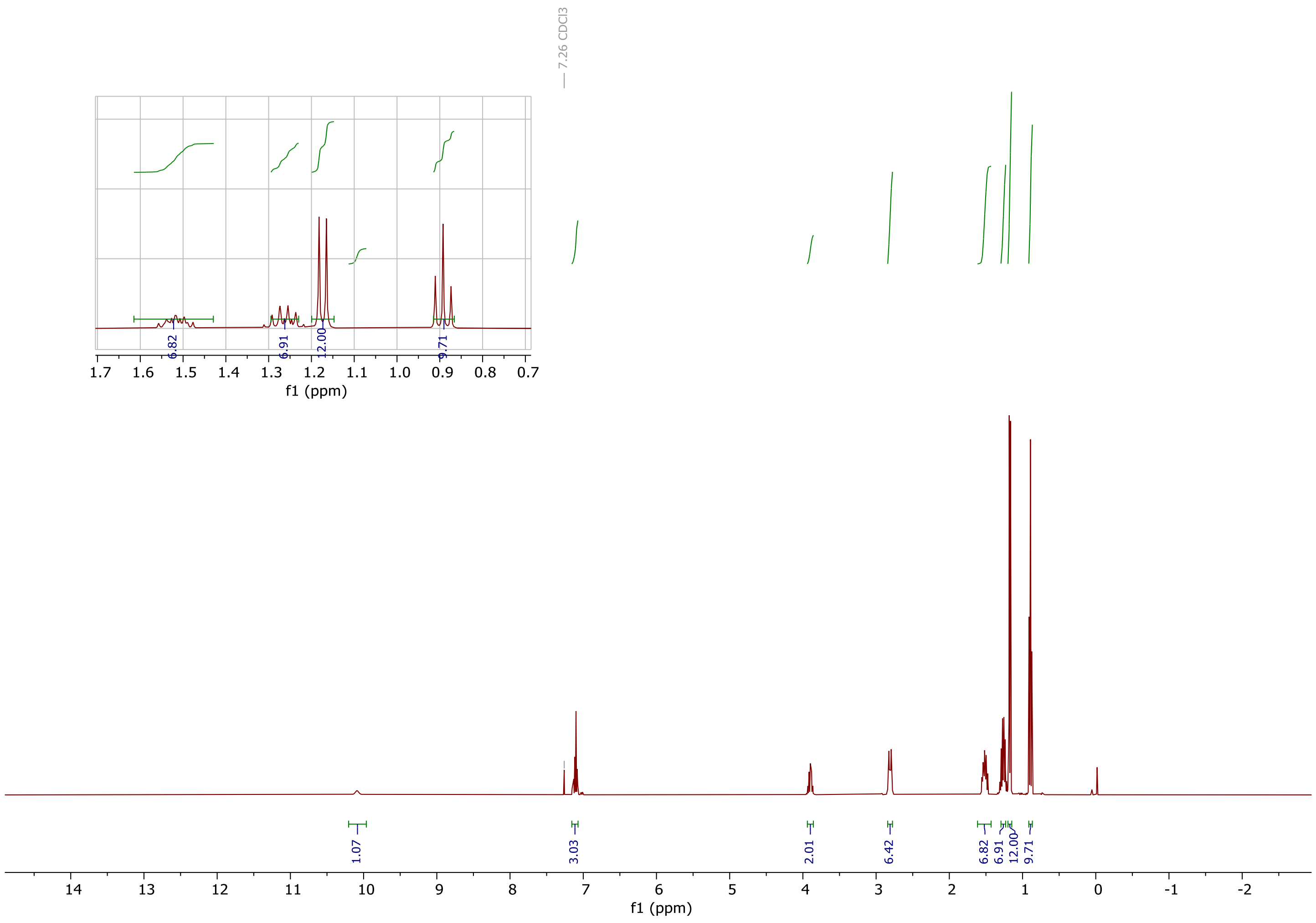

^{13}C NMR spectrum of **2e** (101 MHz, $CDCl_3$)

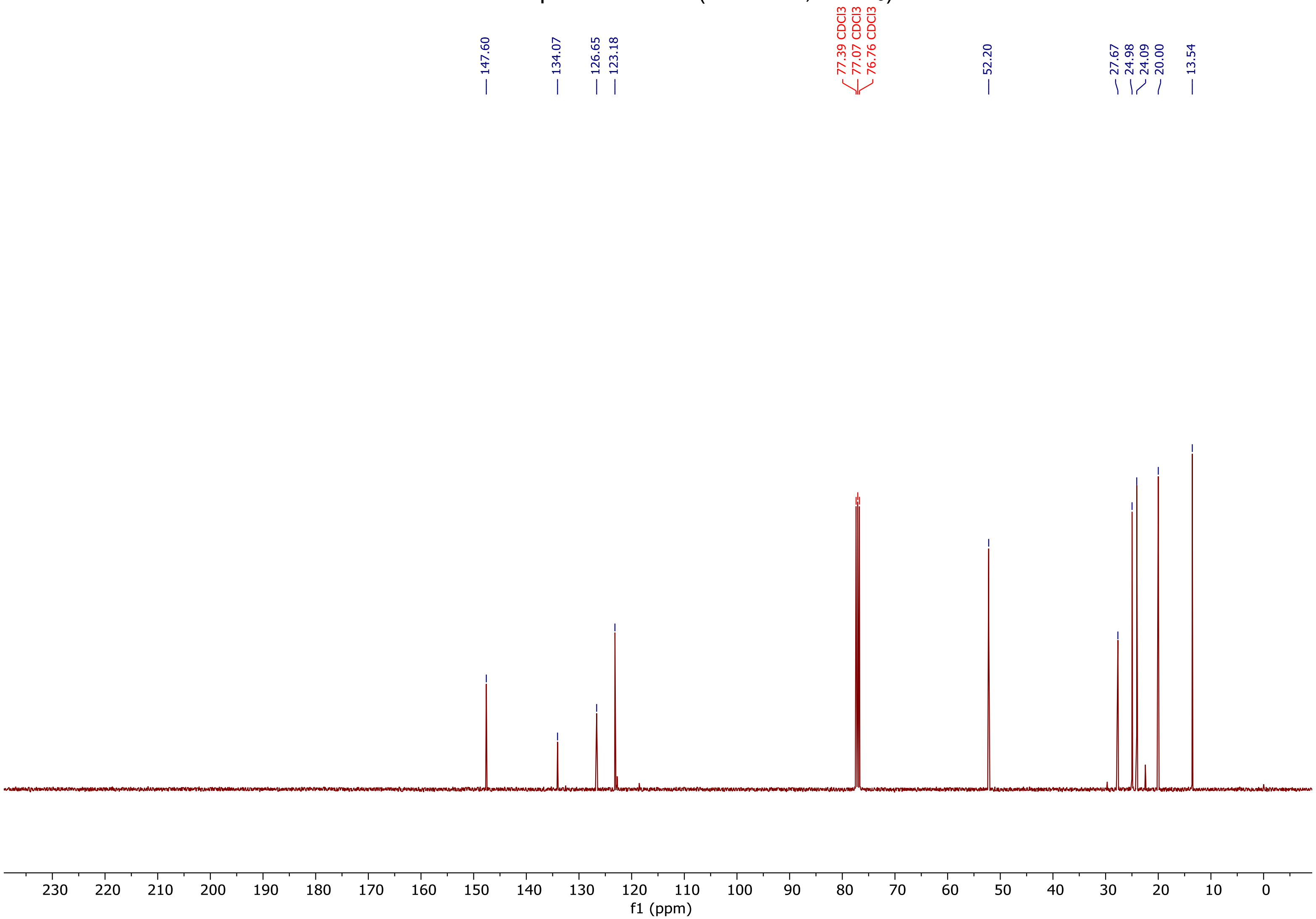

^{1}H NMR spectrum of **2f** (400 MHz, $CDCl_3$)

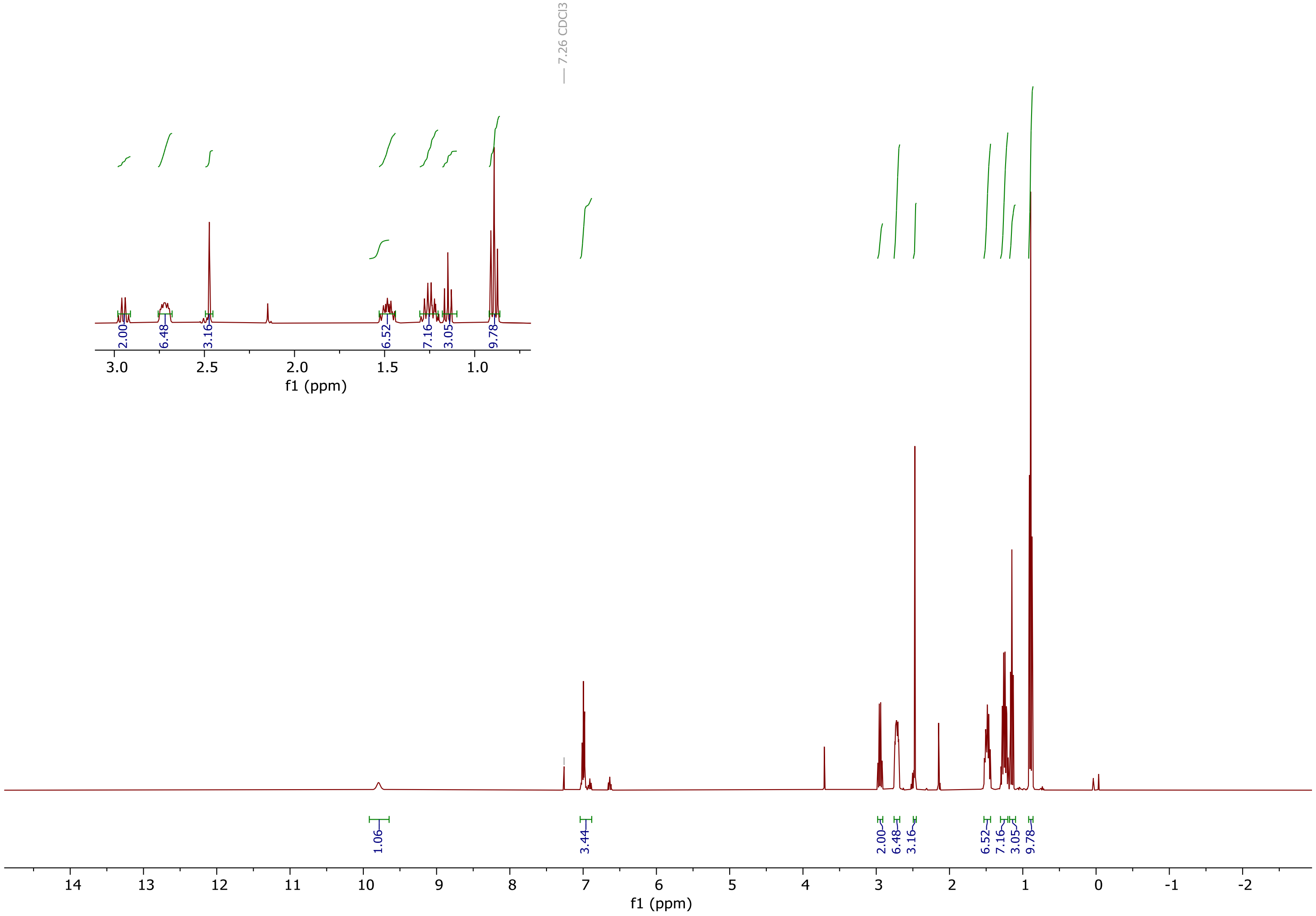

^{13}C NMR spectrum of **2f** (101 MHz, $CDCl_3$)

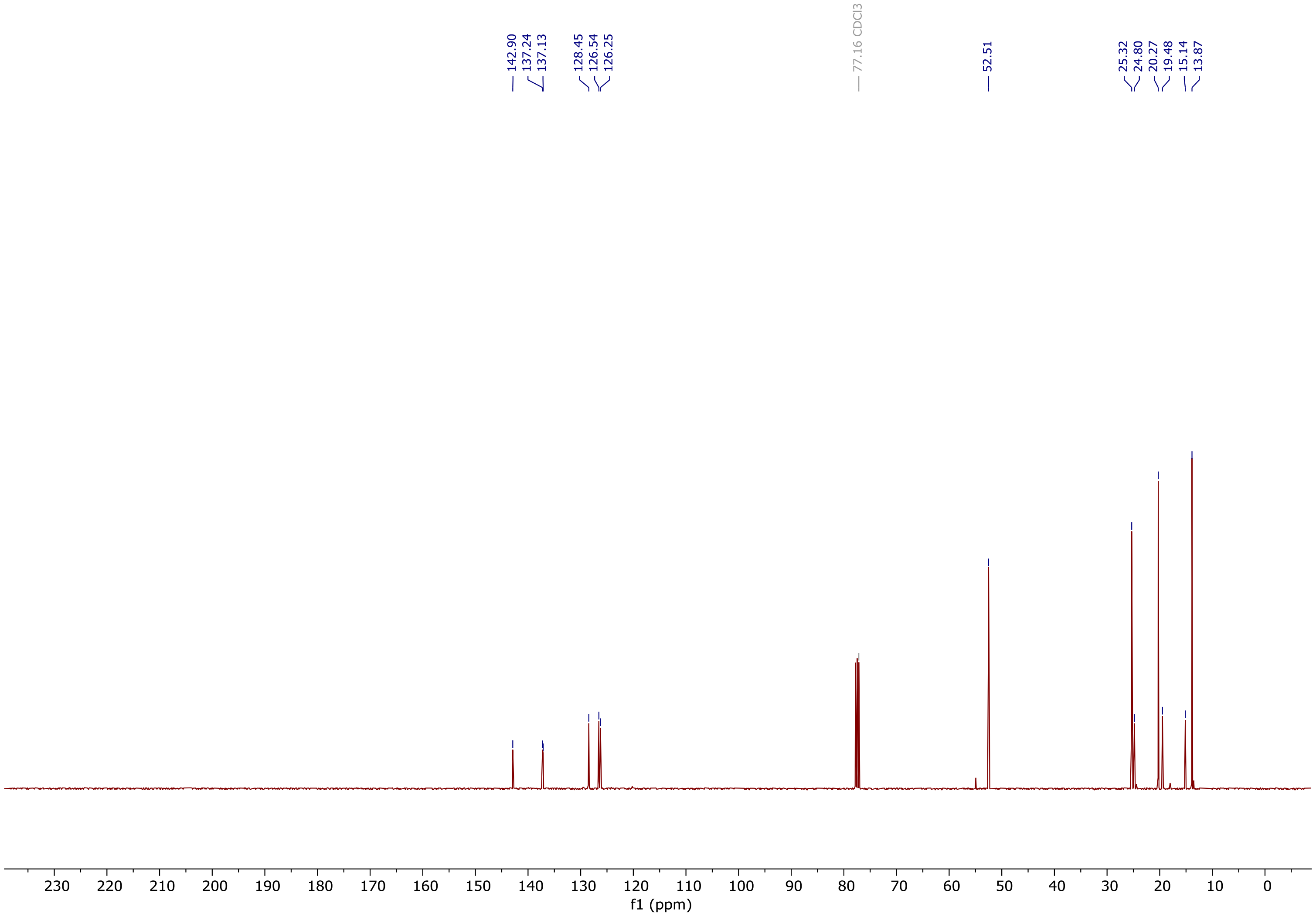

^{1}H NMR spectrum of **4a** (400 MHz, MeOD)

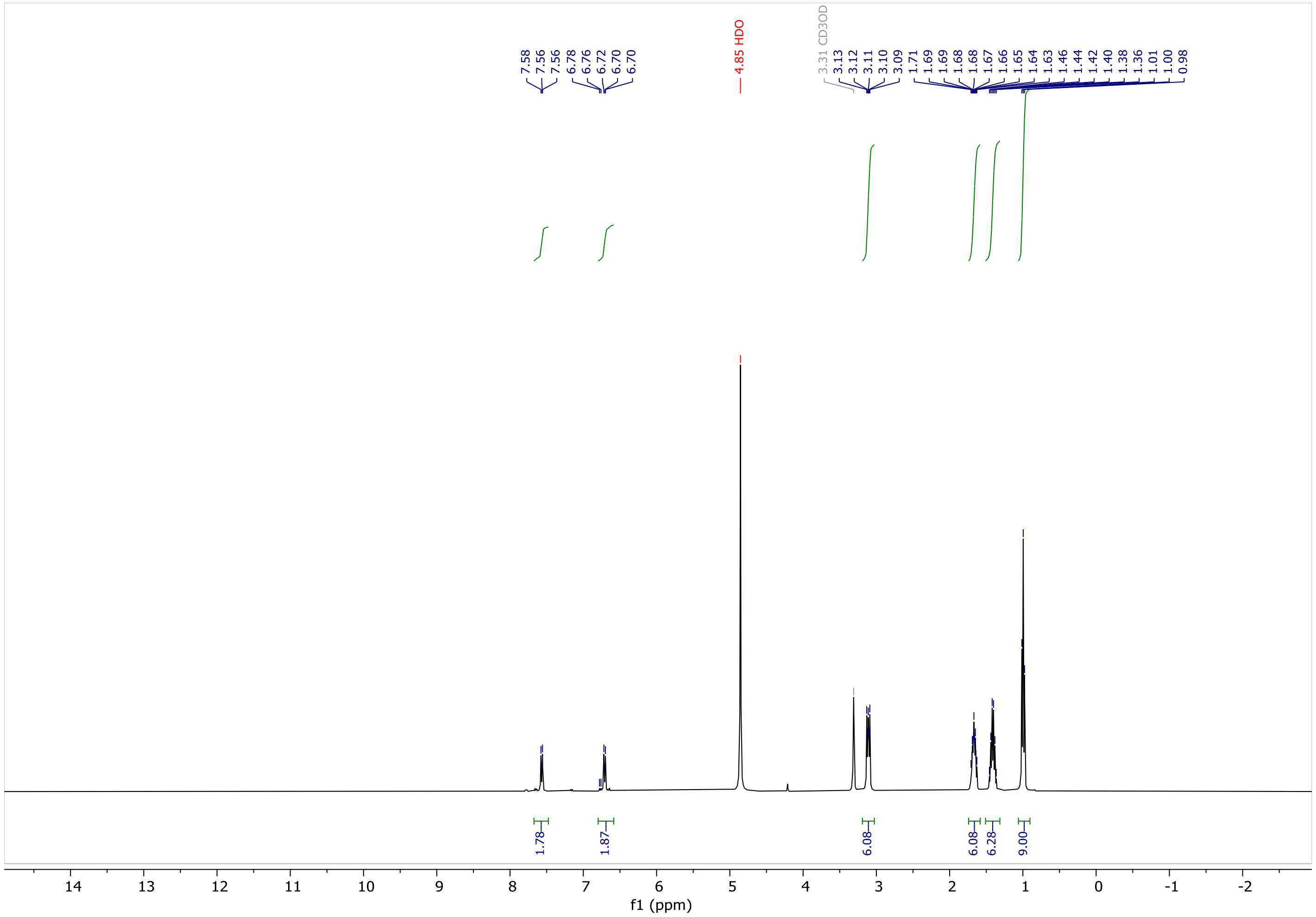

^{13}C NMR spectrum of **4a** (101 MHz, MeOD)

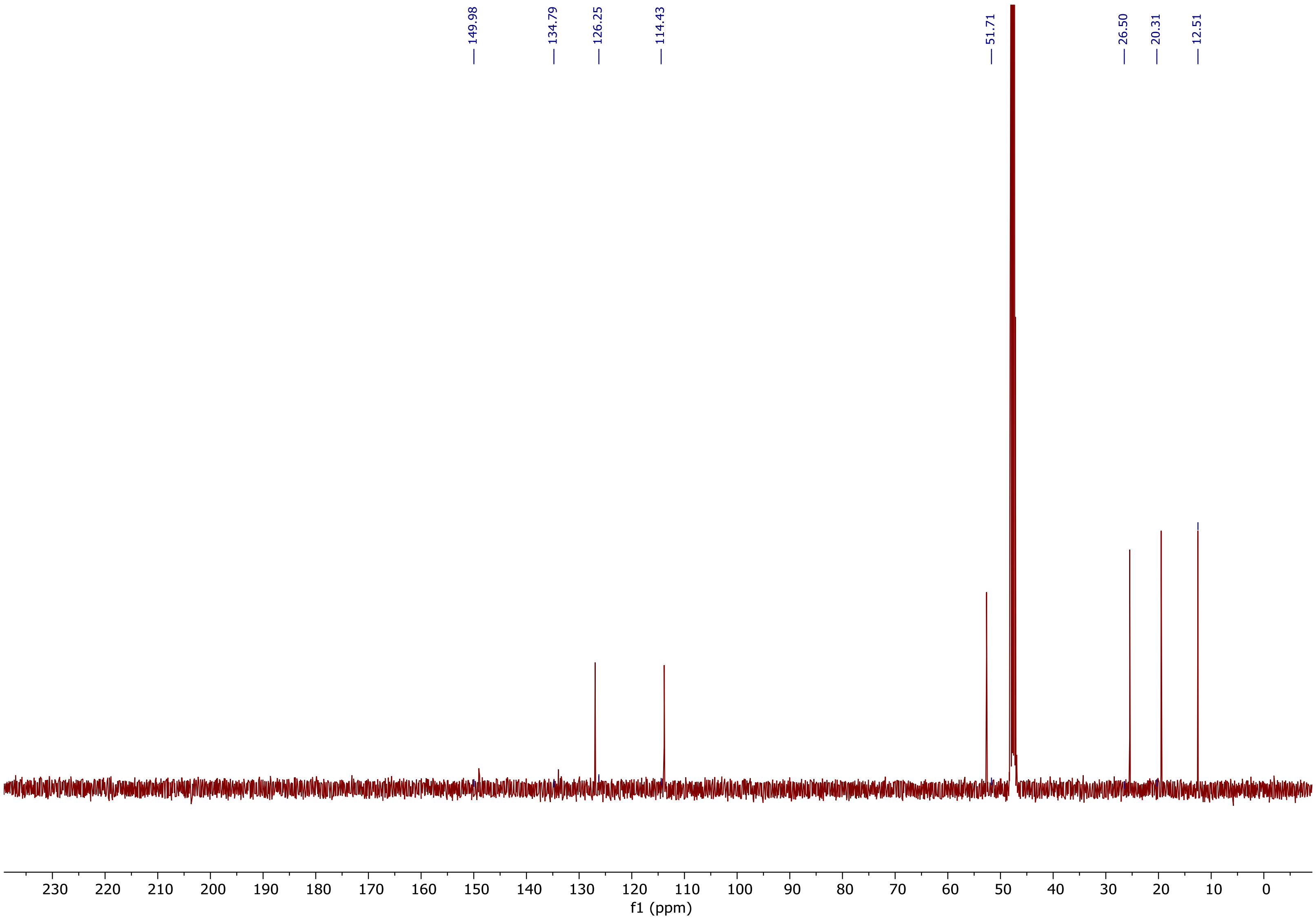

^{1}H NMR spectrum of **4b** (400 MHz, MeOD)

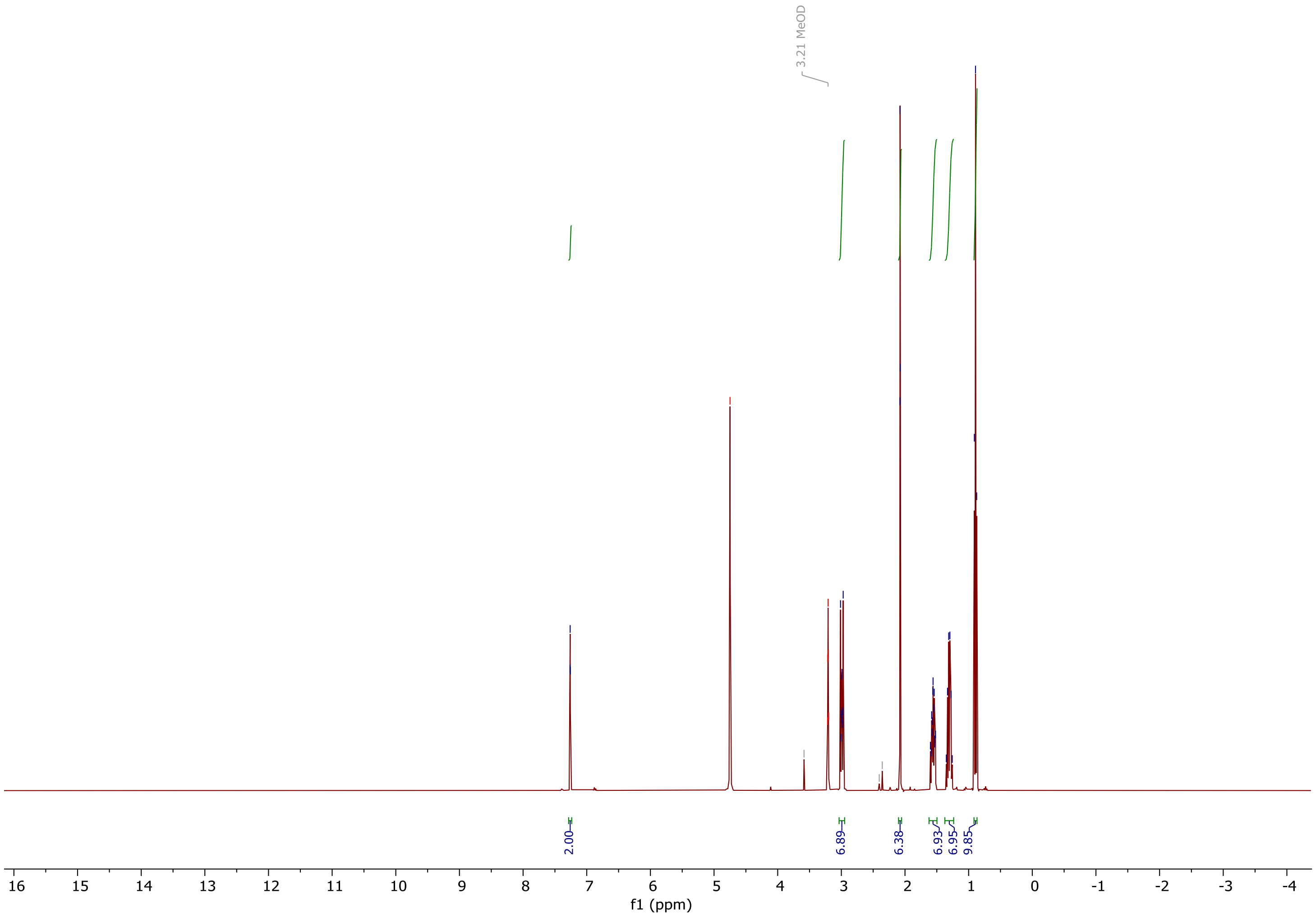

^{13}C NMR spectrum of **4b** (101 MHz, MeOD)

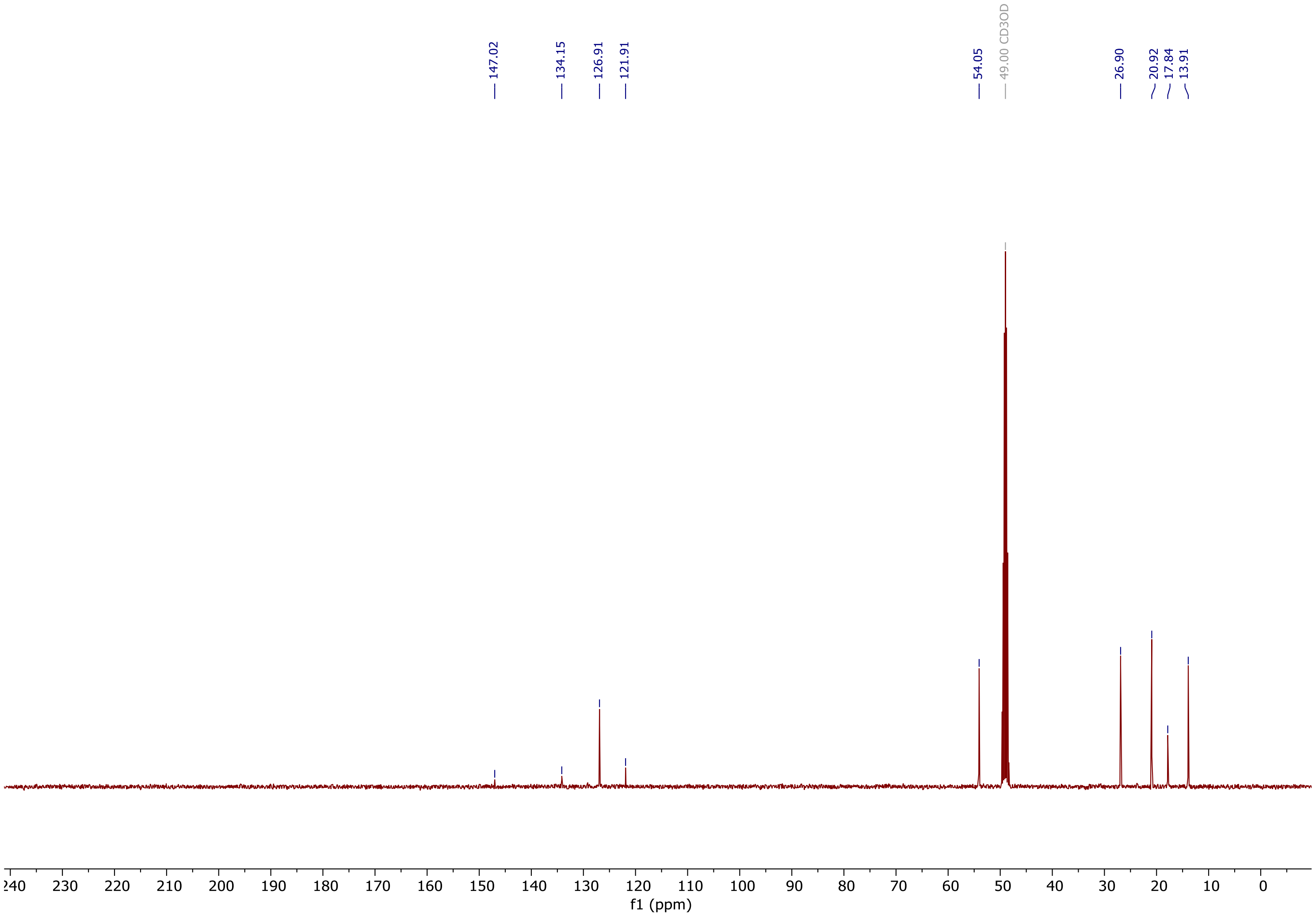

^{1}H NMR spectrum of **4d** (400 MHz, MeOD)

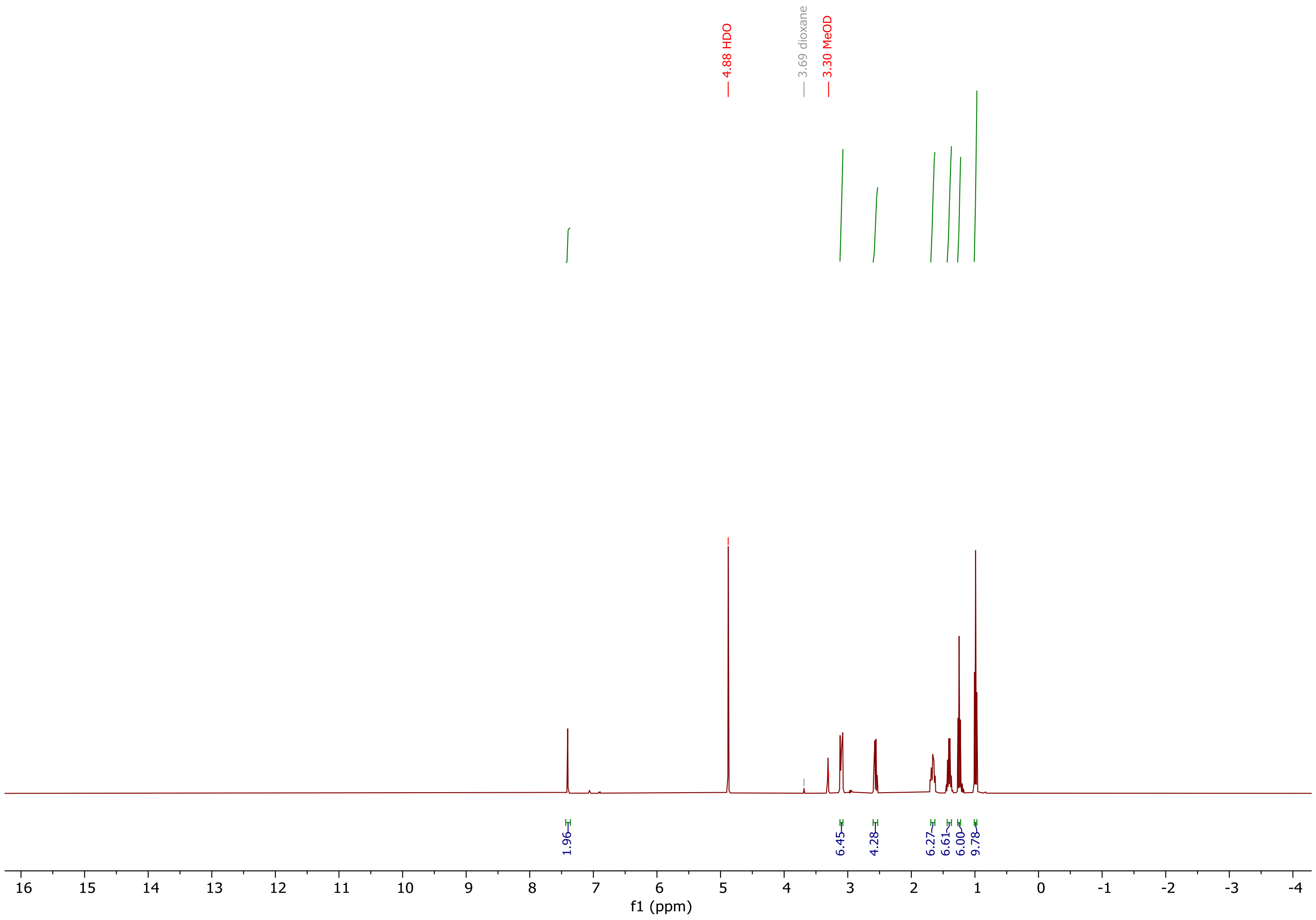

^{13}C NMR spectrum of **4d** (101 MHz, MeOD)

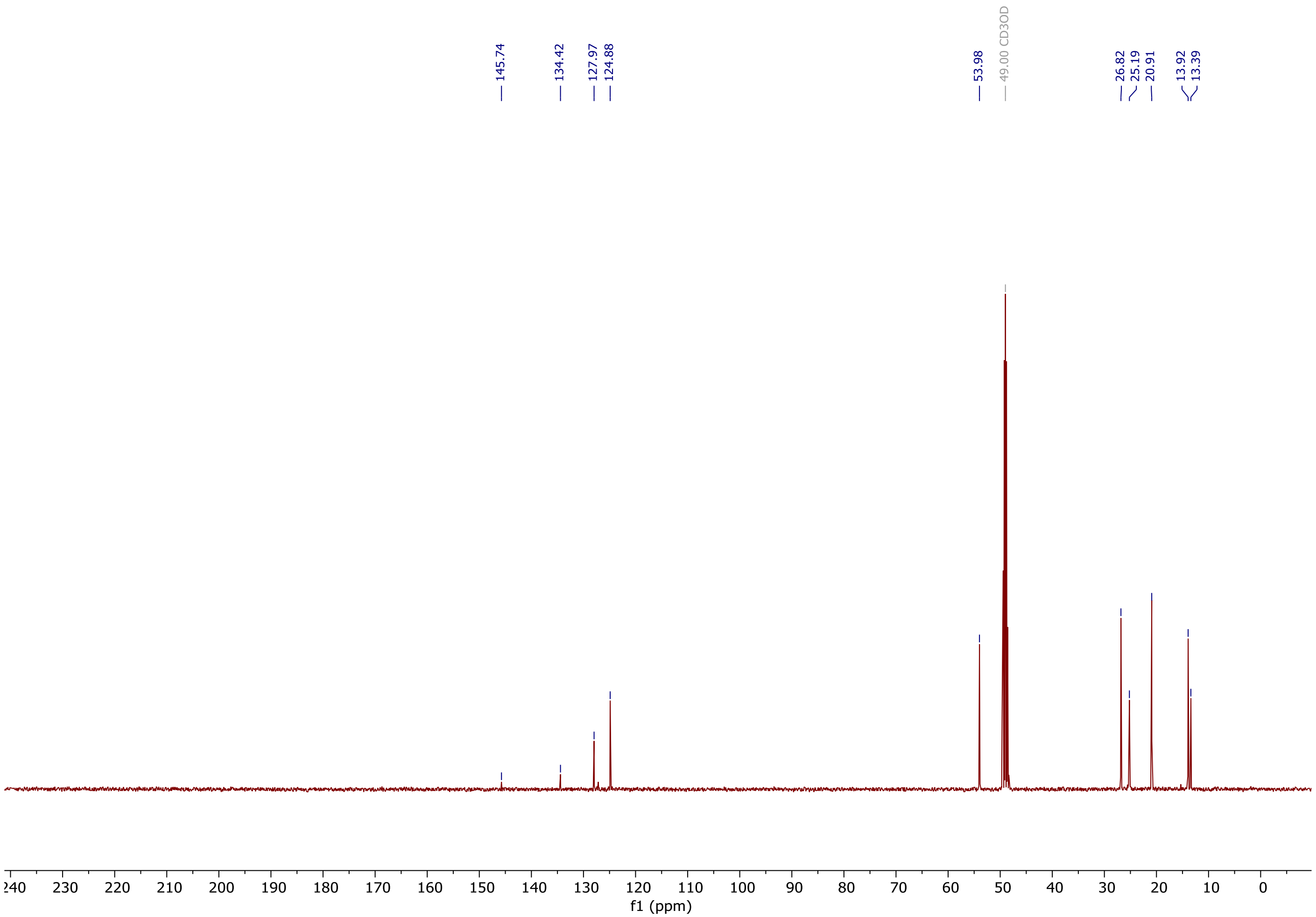

^{1}H NMR spectrum of **4e** (400 MHz, MeOD)

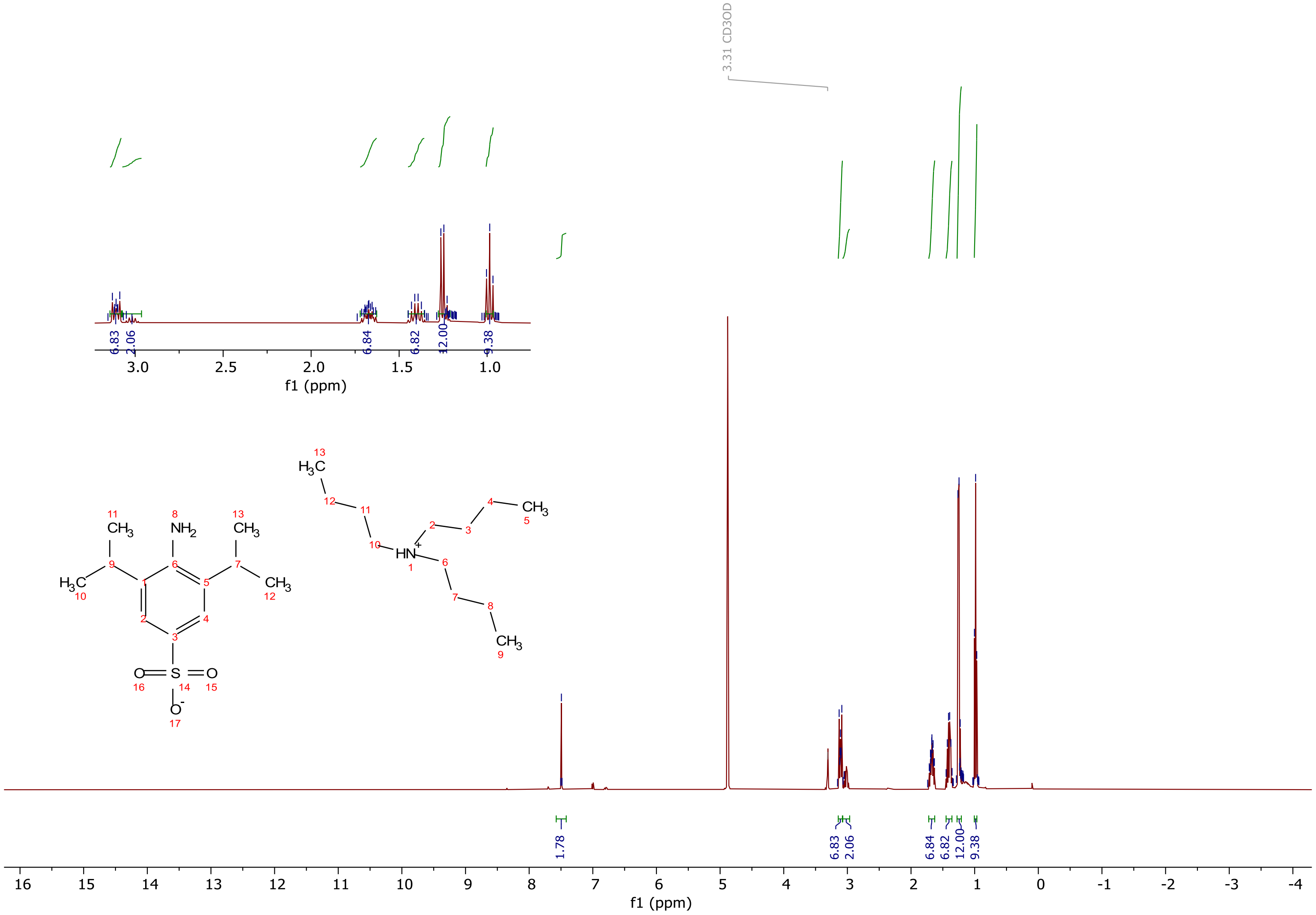

^{13}C NMR spectrum of **4e** (101 MHz, MeOD)

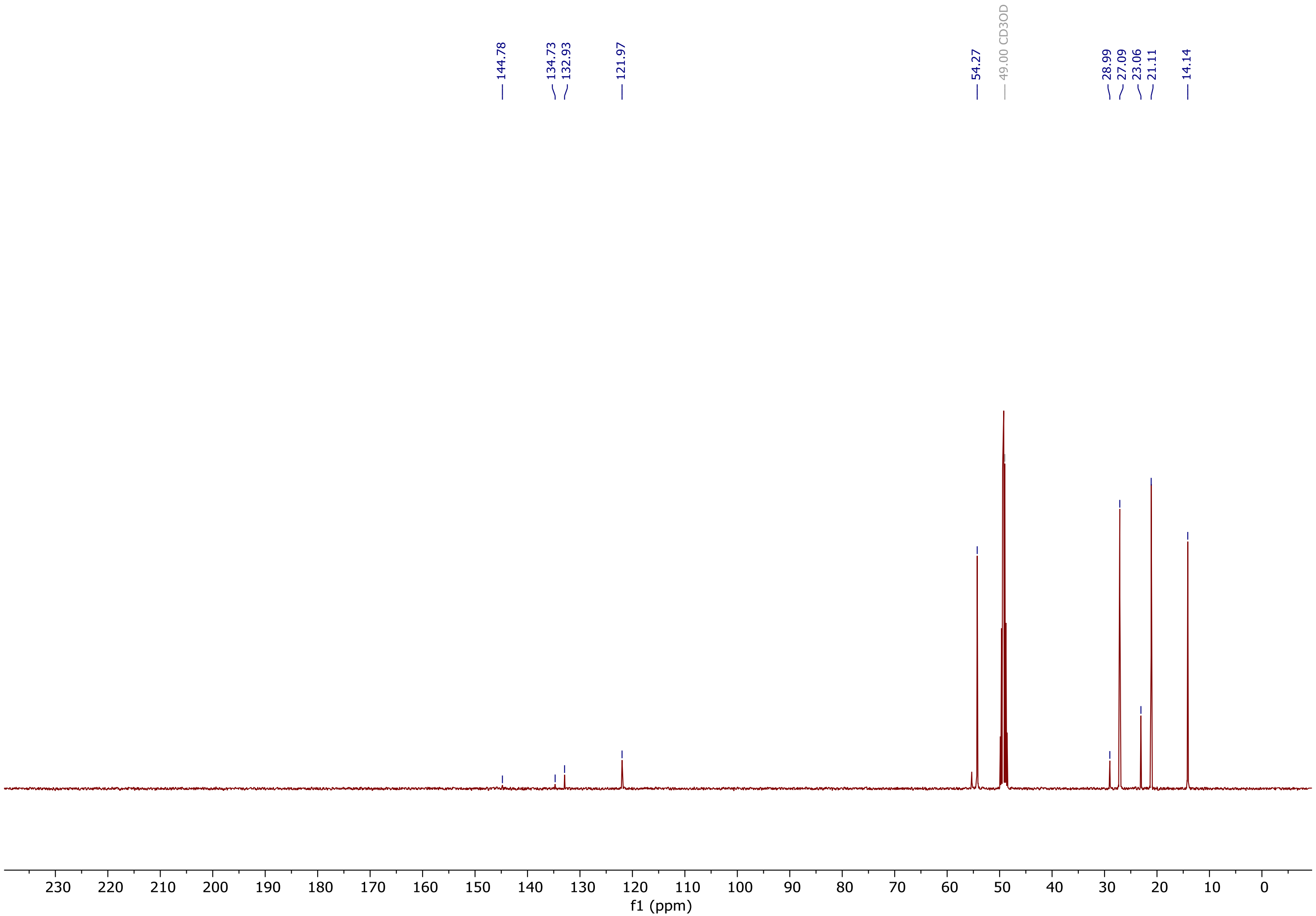

^{1}H NMR spectrum of **4f** (400 MHz, MeOD)

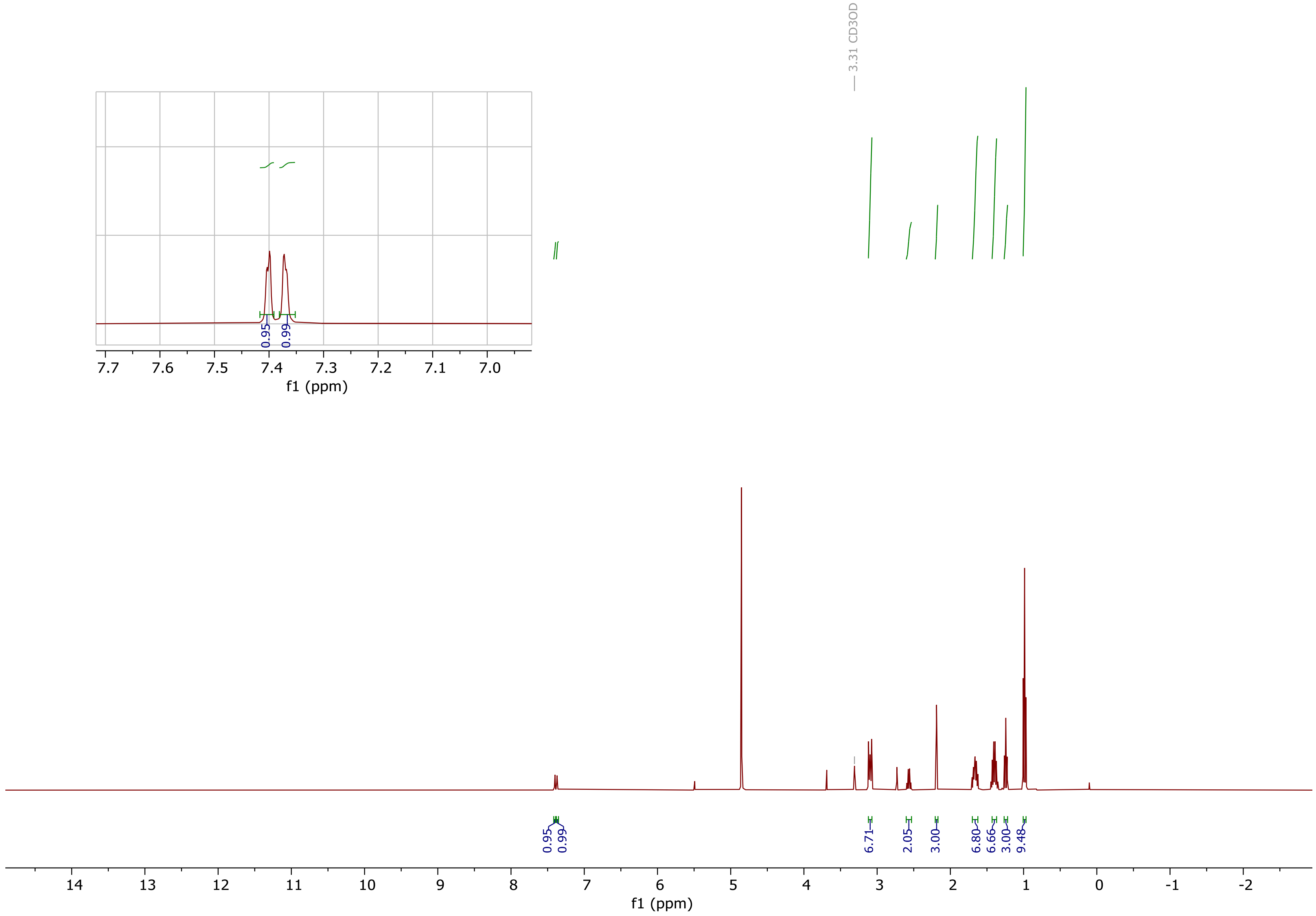

^{13}C NMR spectrum of **4f** (101 MHz, MeOD)

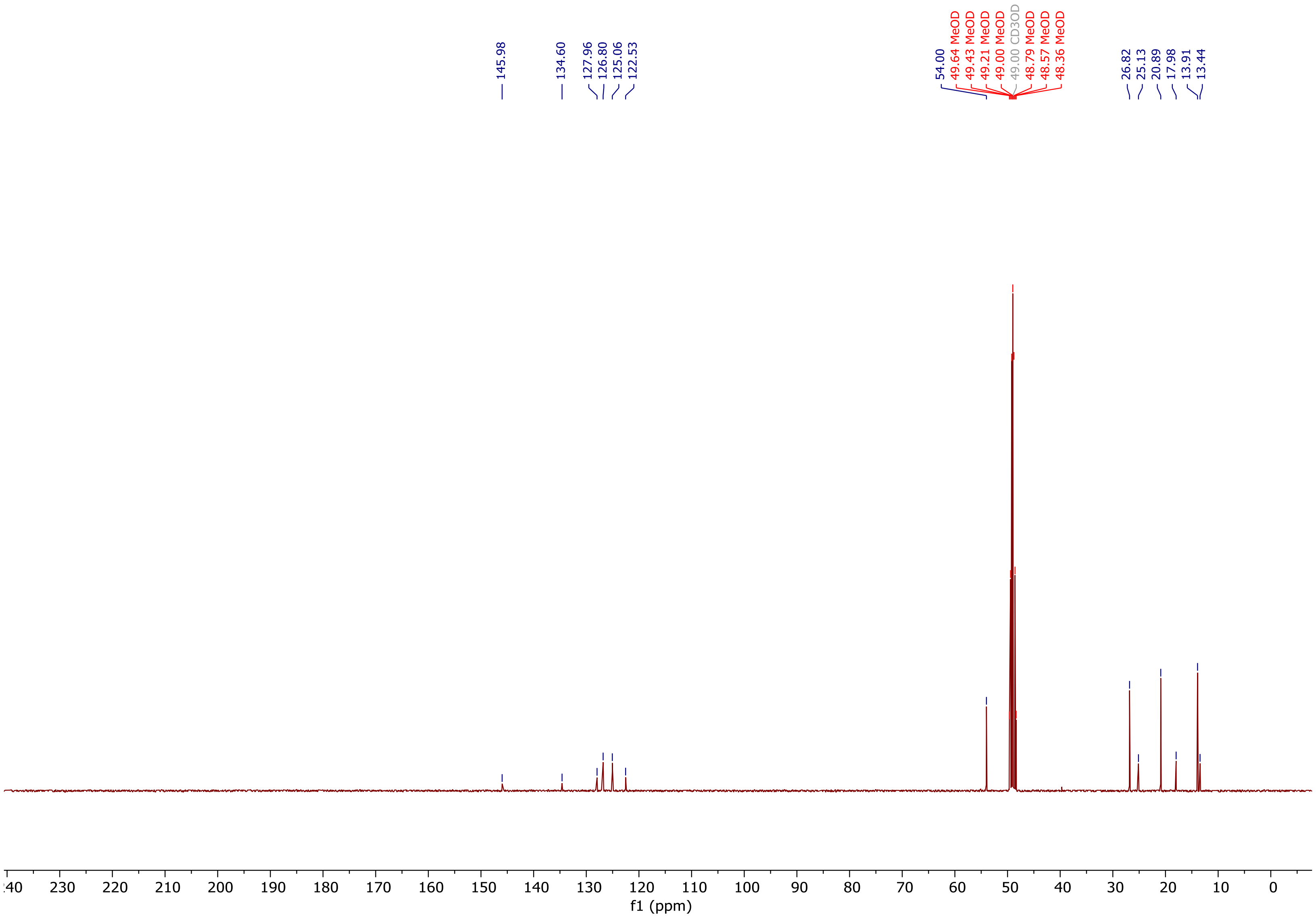

^{1}H NMR spectrum of **4g** (400 MHz, MeOD)

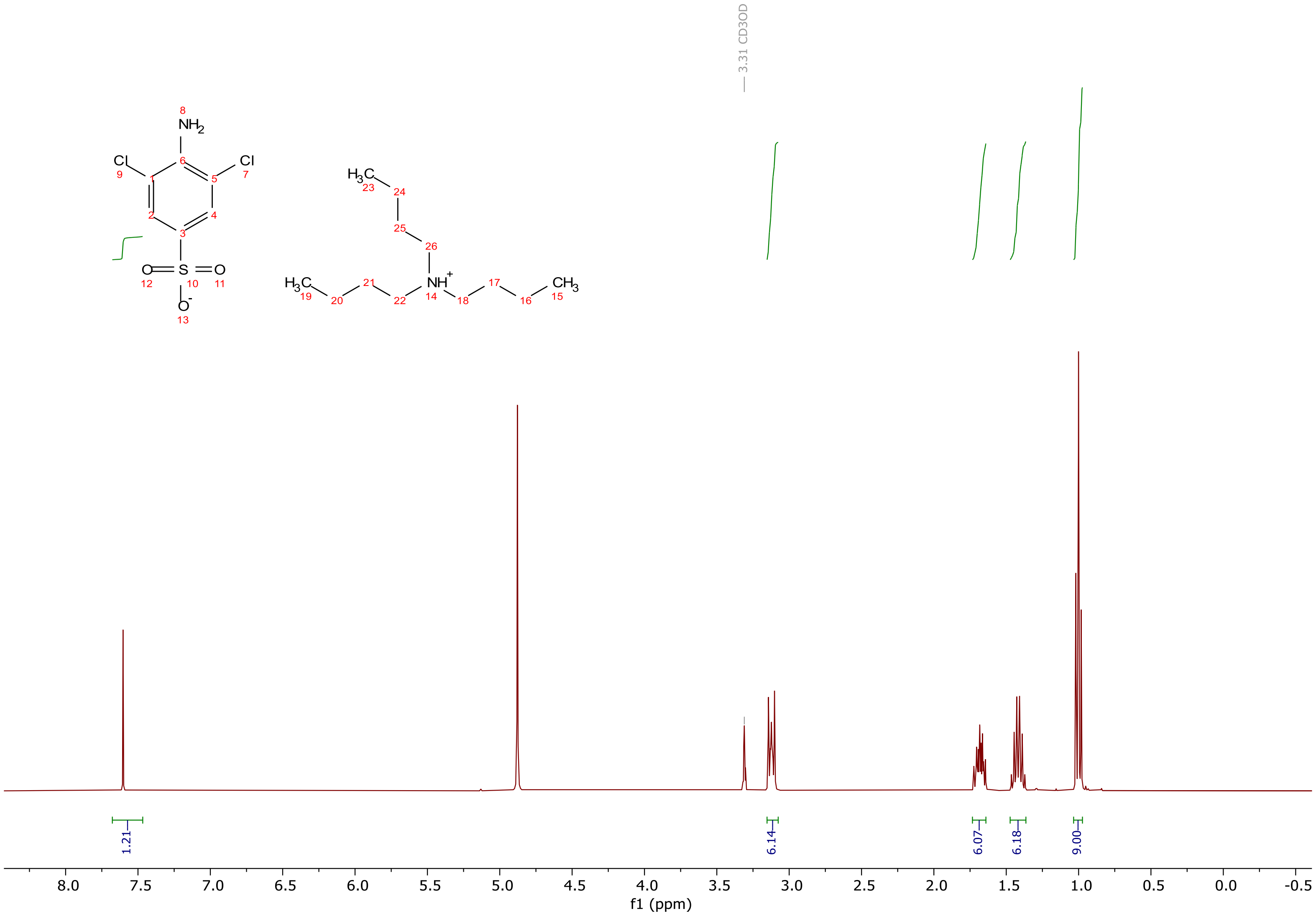

^{13}C NMR spectrum of **4g** (101 MHz, MeOD)

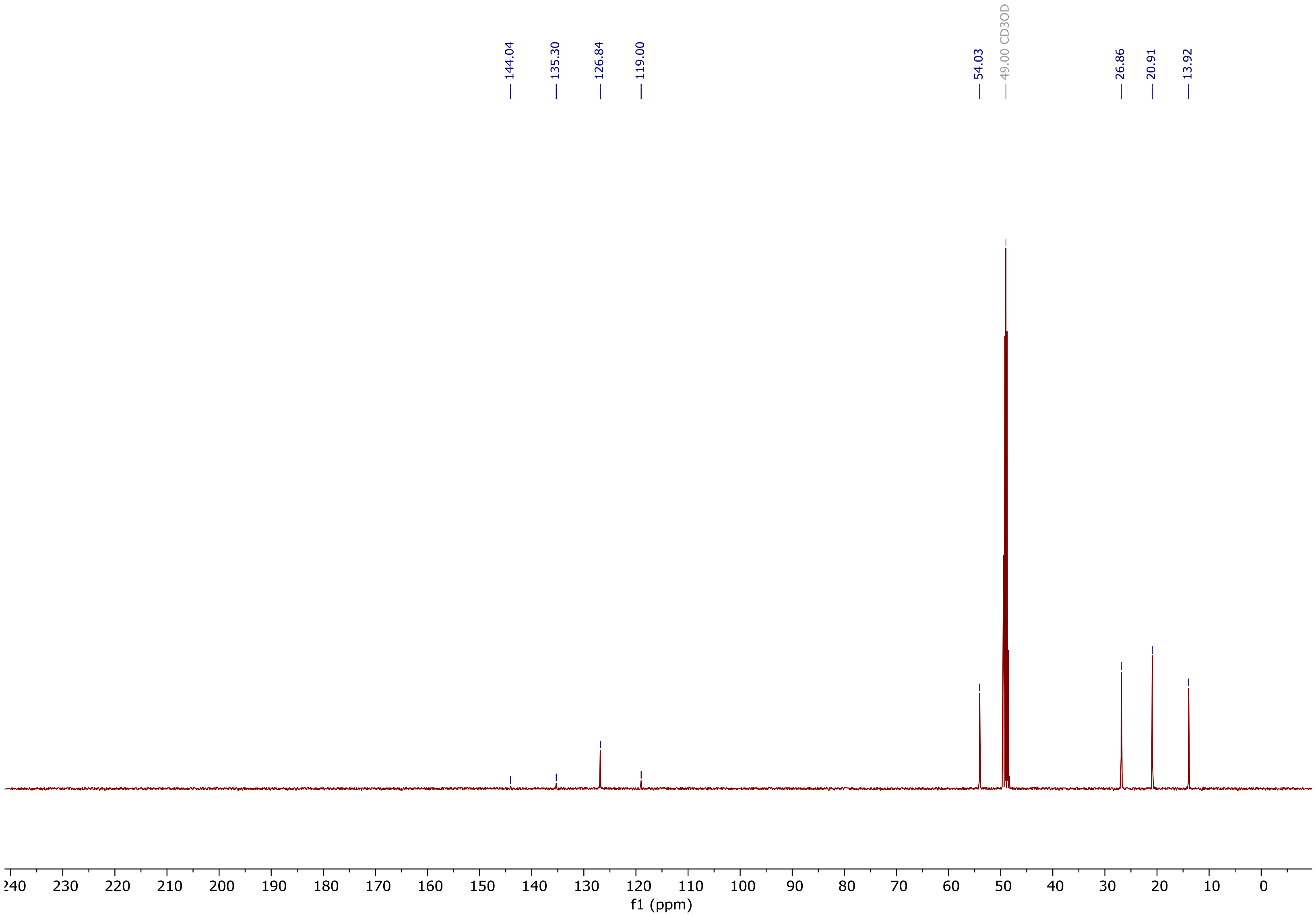

^{1}H NMR spectrum of **4h** (400 MHz, MeOD)

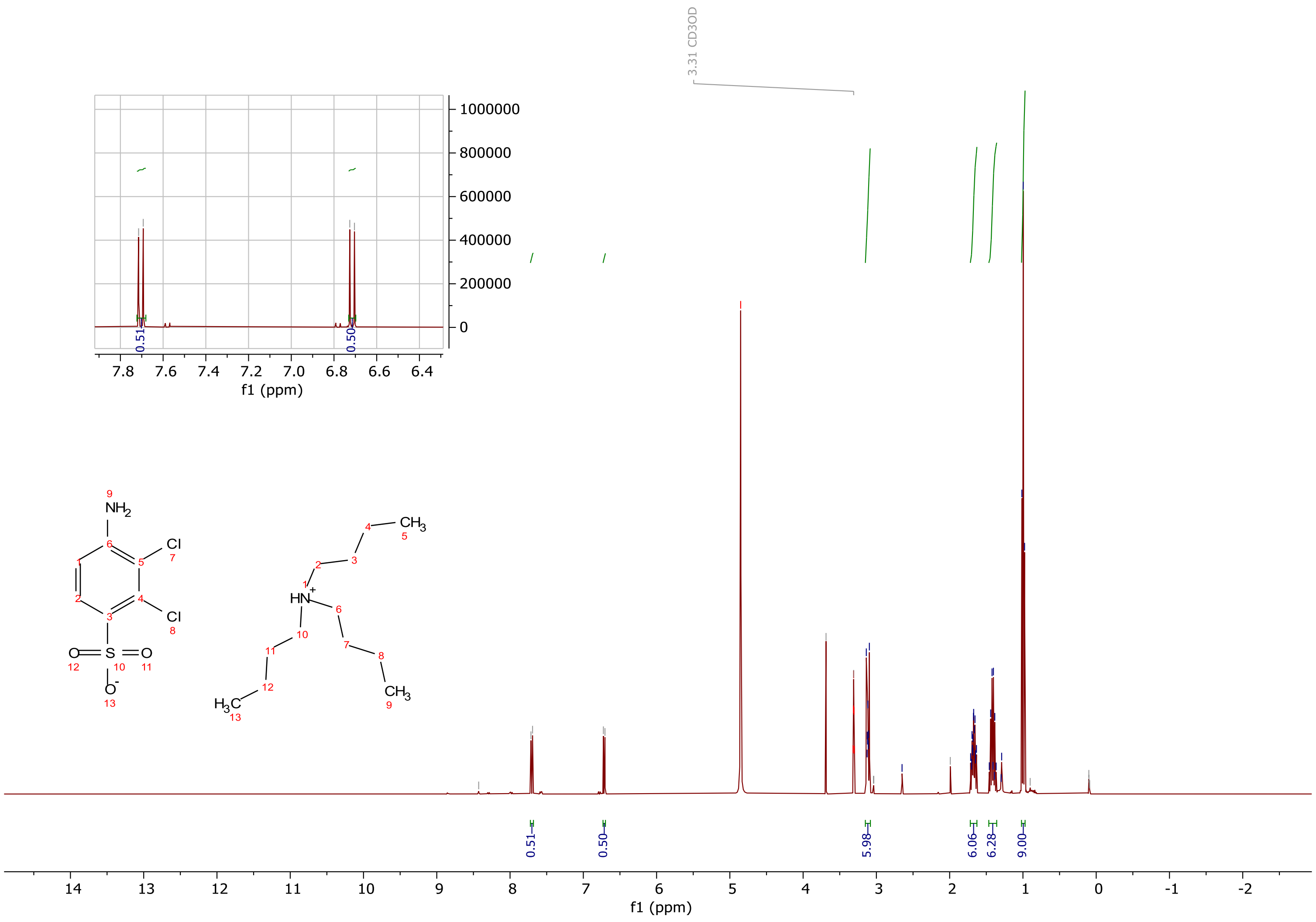

^{13}C NMR spectrum of **4h** (101 MHz, MeOD)

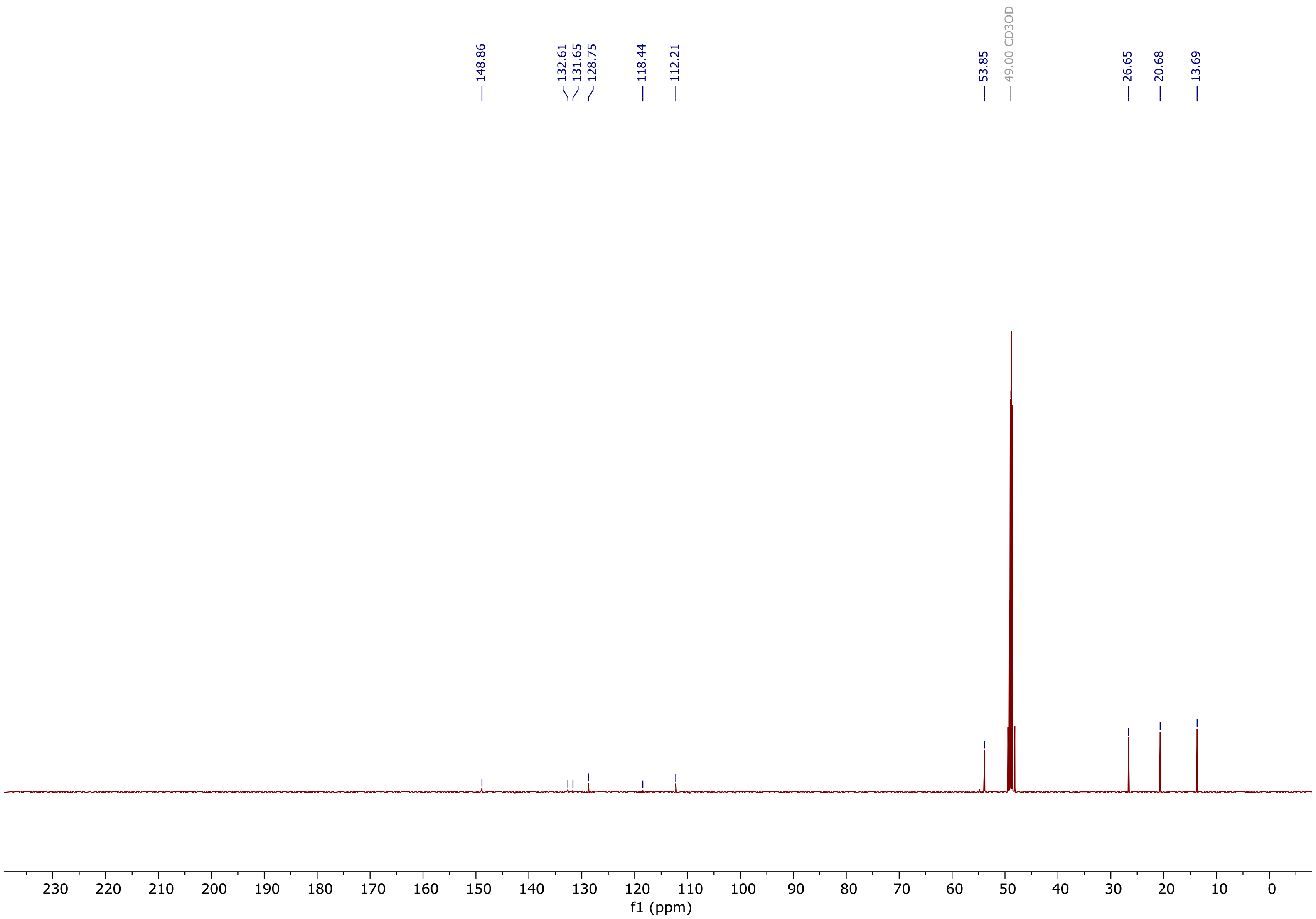

^{1}H NMR spectrum of **4i** (400 MHz, MeOD)

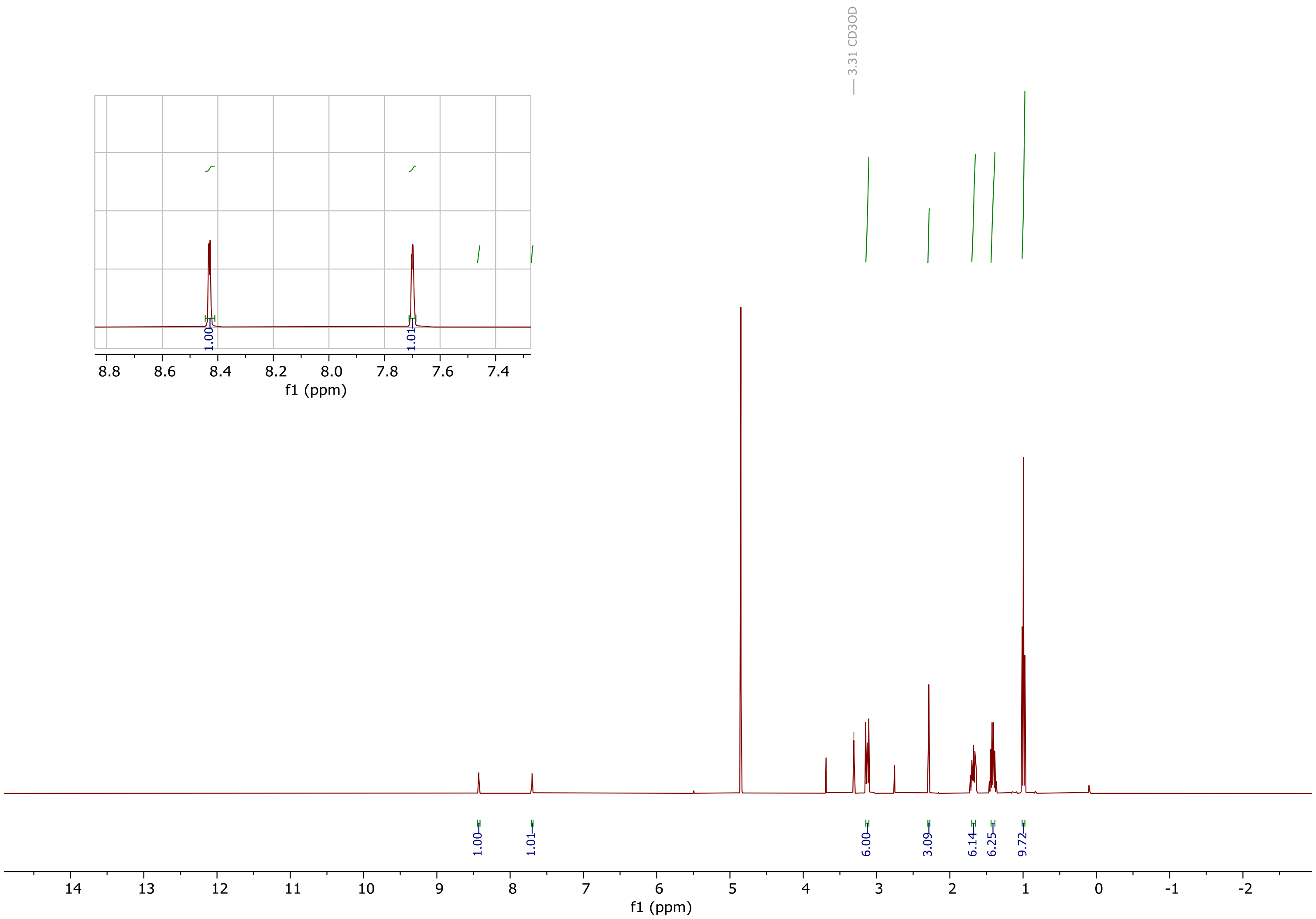

^{13}C NMR spectrum of **4i** (101 MHz, MeOD)

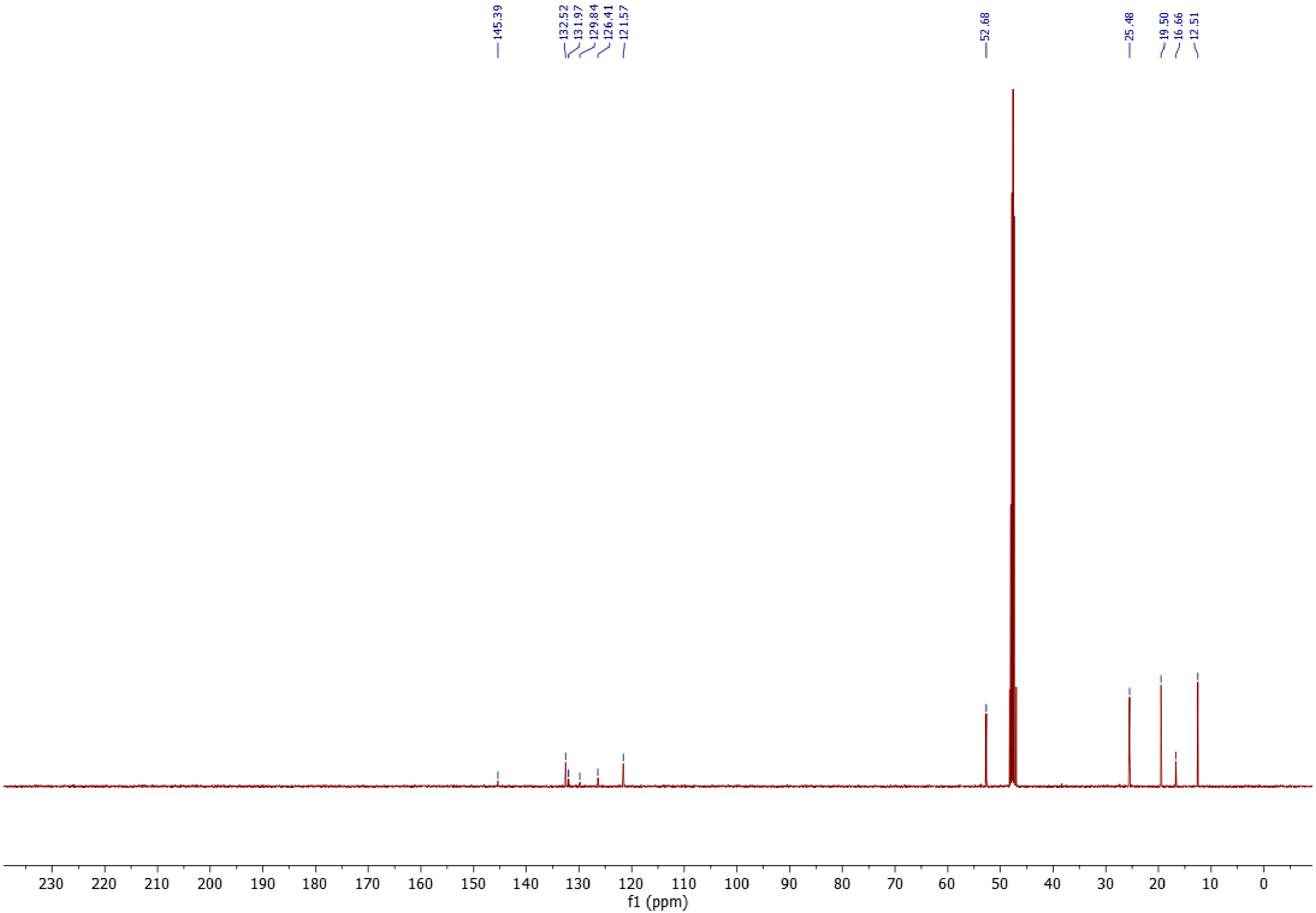

^{1}H NMR spectrum of **4j** (400 MHz, MeOD)

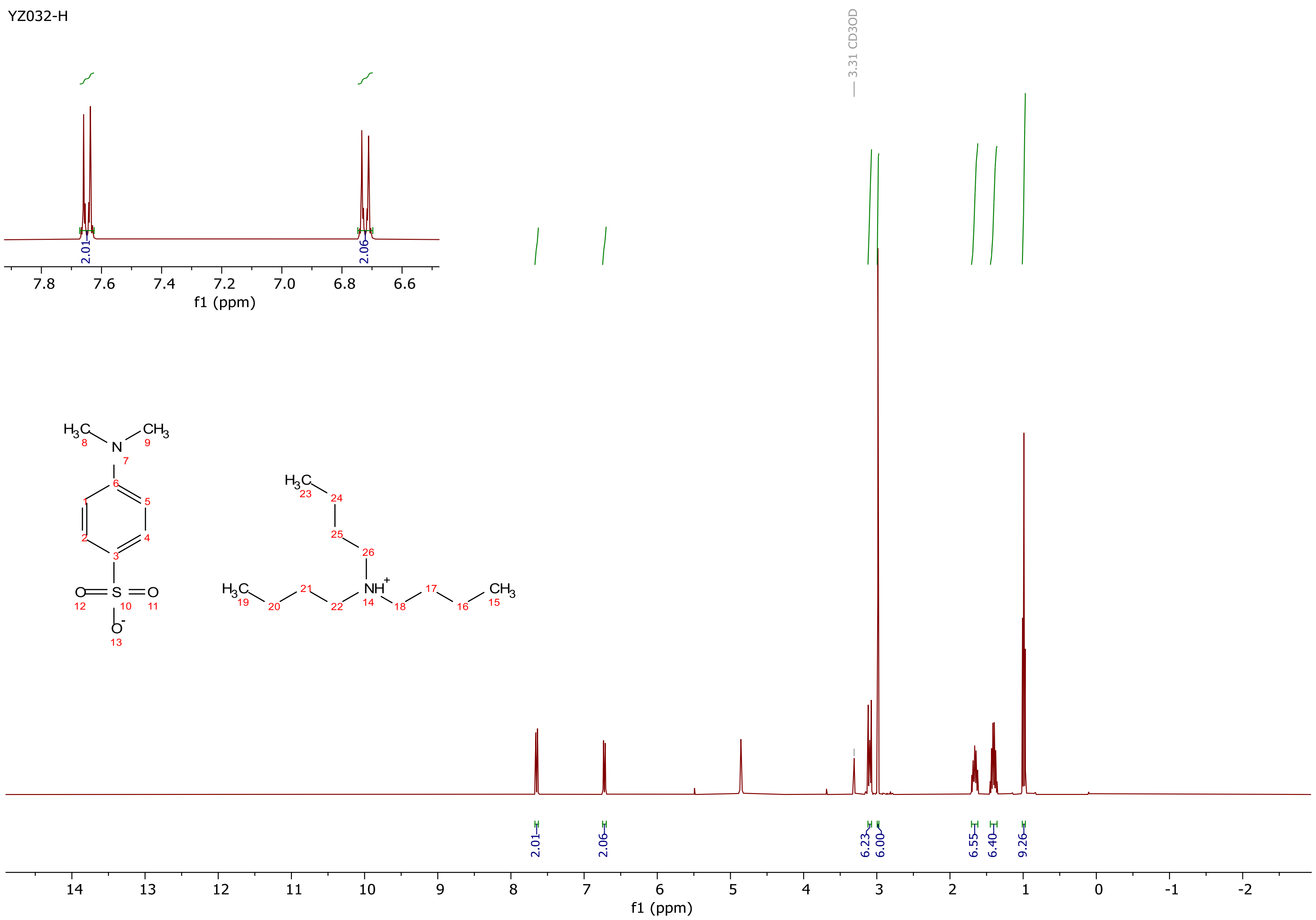

^{13}C NMR spectrum of **4j** (101 MHz, MeOD)

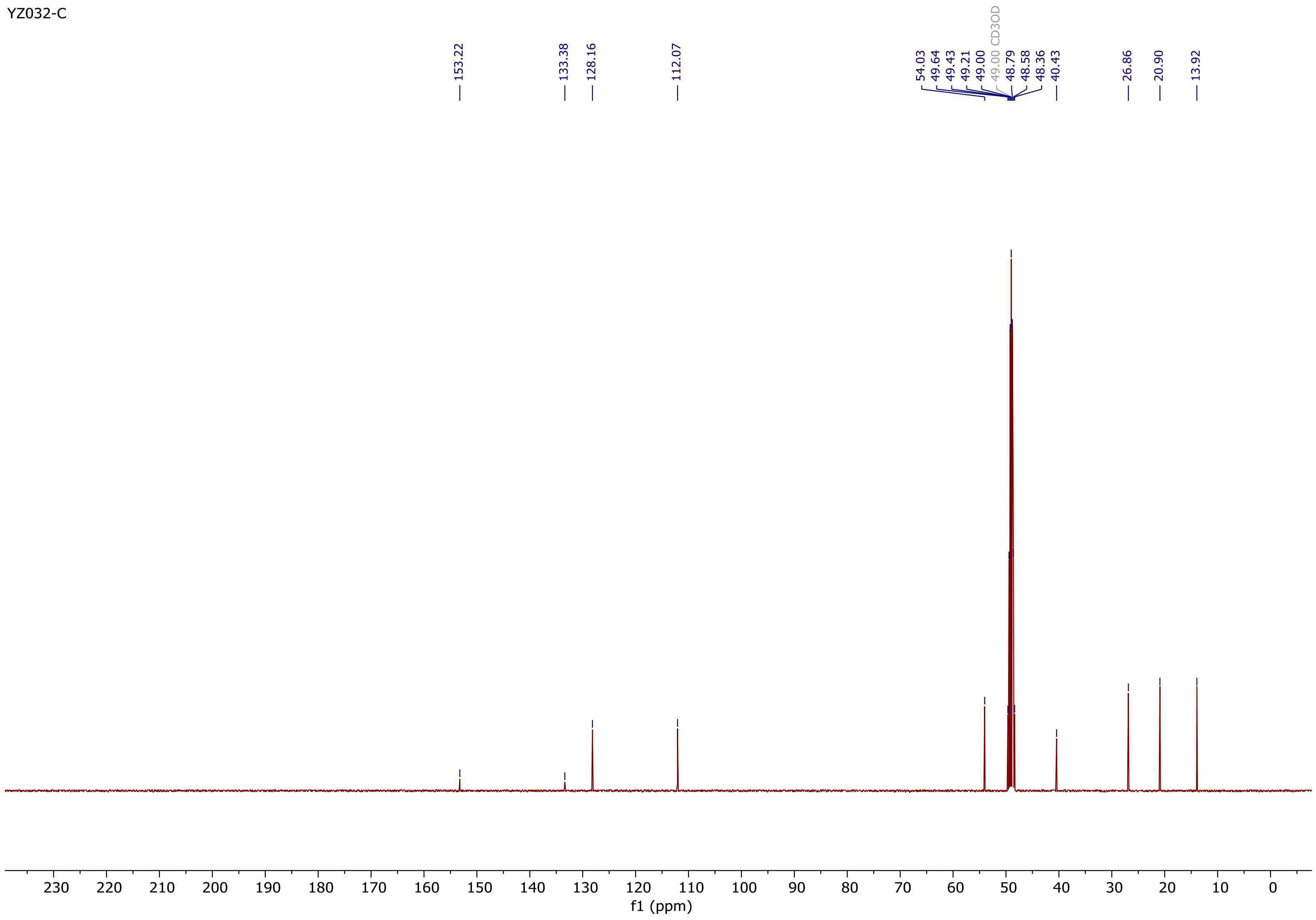

^{1}H NMR spectrum of **5** (400 MHz, MeOD)

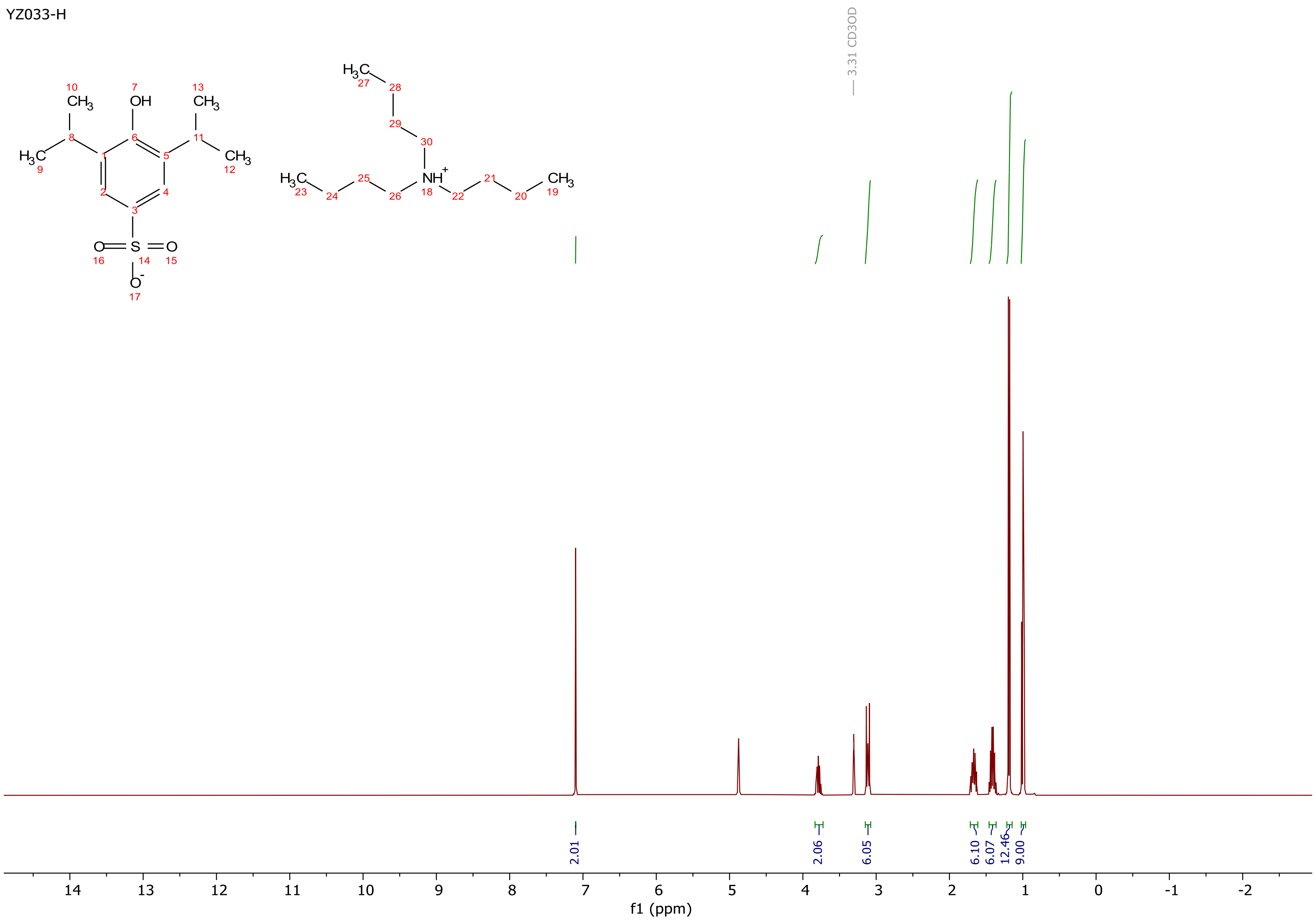

^{13}C NMR spectrum of **5** (101 MHz, MeOD)

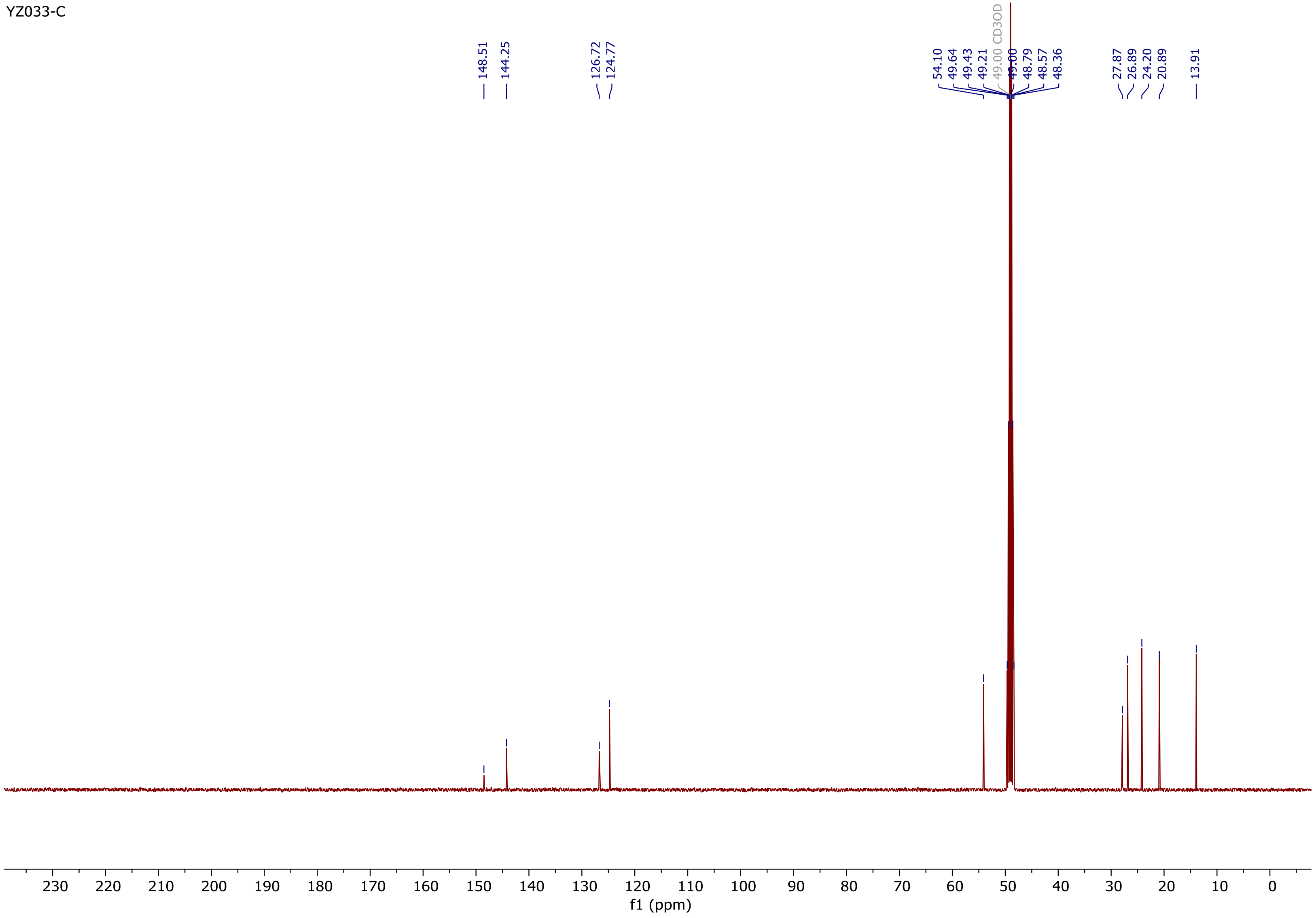

^{1}H NMR spectrum of **6** (400 MHz, MeOD4)

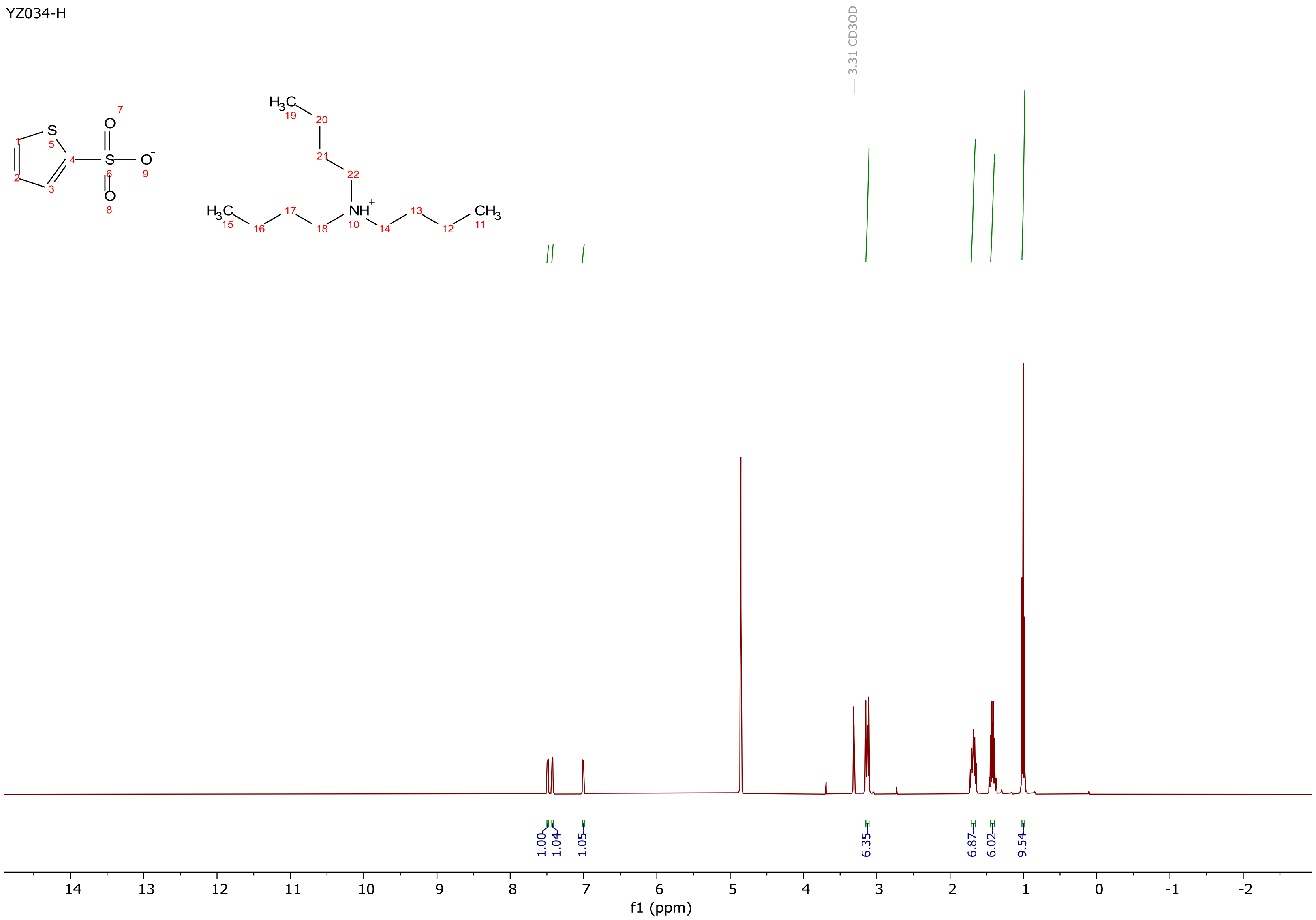

^{13}C NMR spectrum of **6** (101 MHz, MeOD)

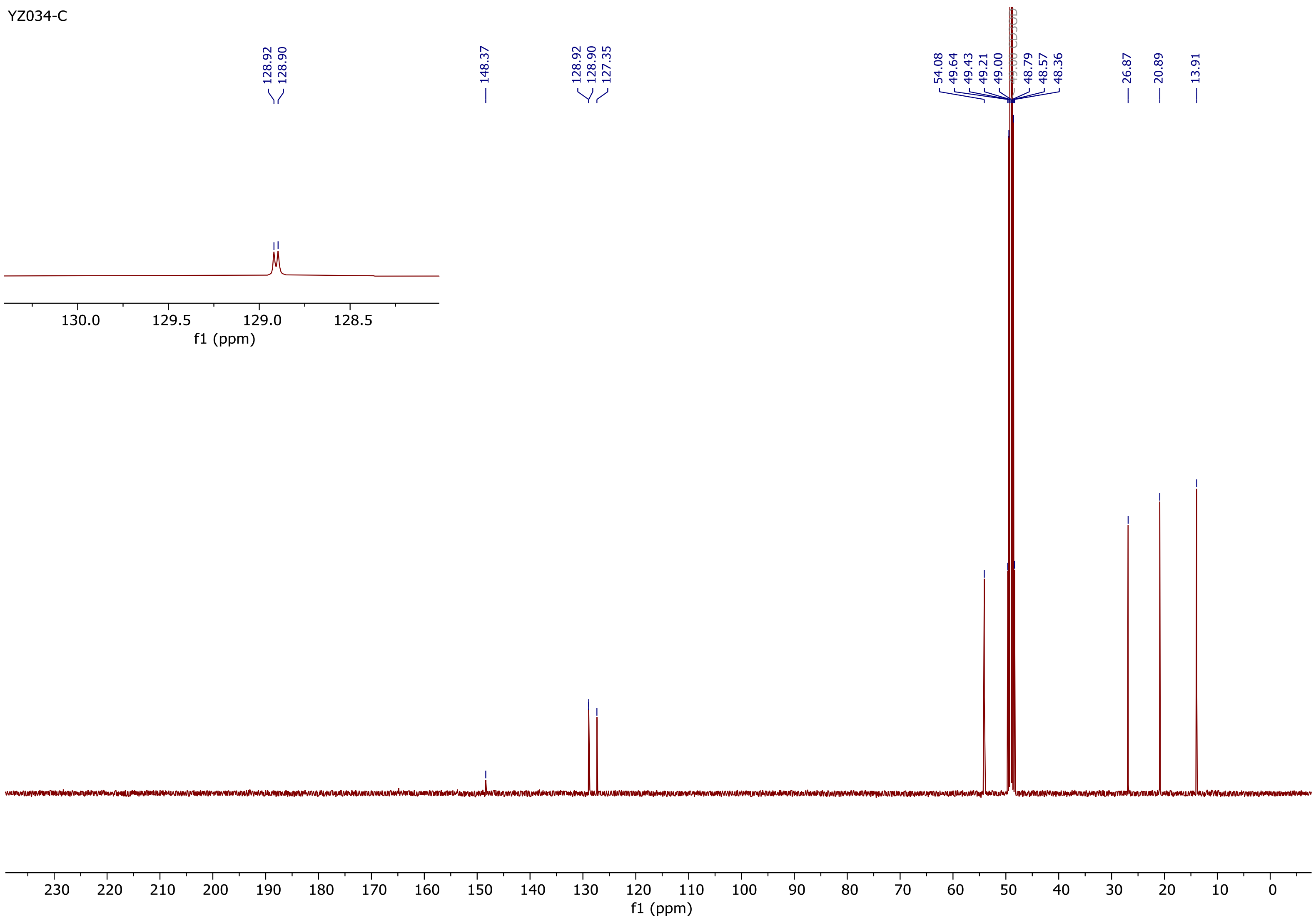

^{1}H NMR spectrum of **7** (400 MHz, MeOD)

YZ036-H

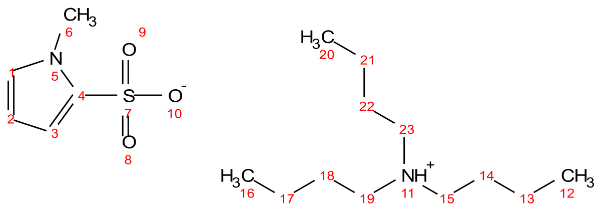

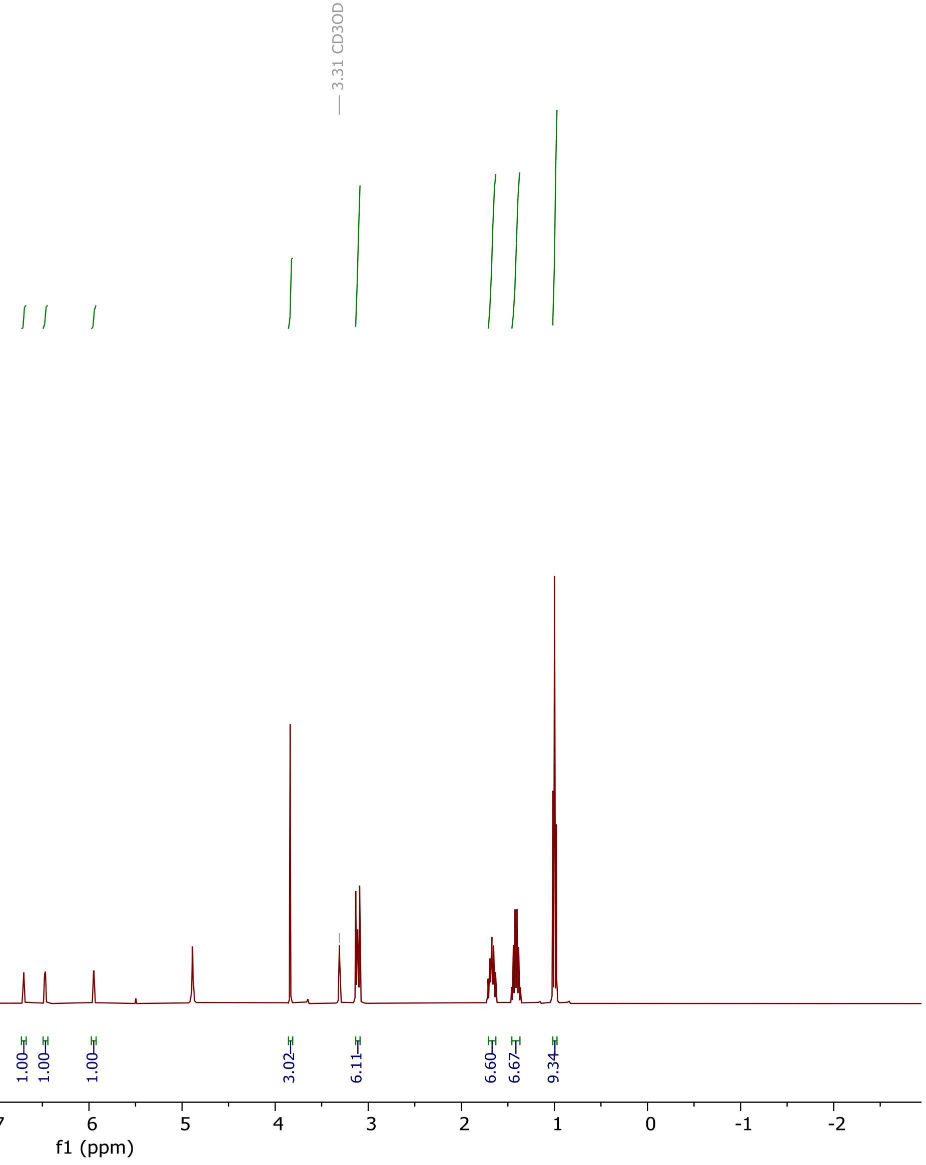

^{13}C NMR spectrum of **7** (101 MHz, MeOD)

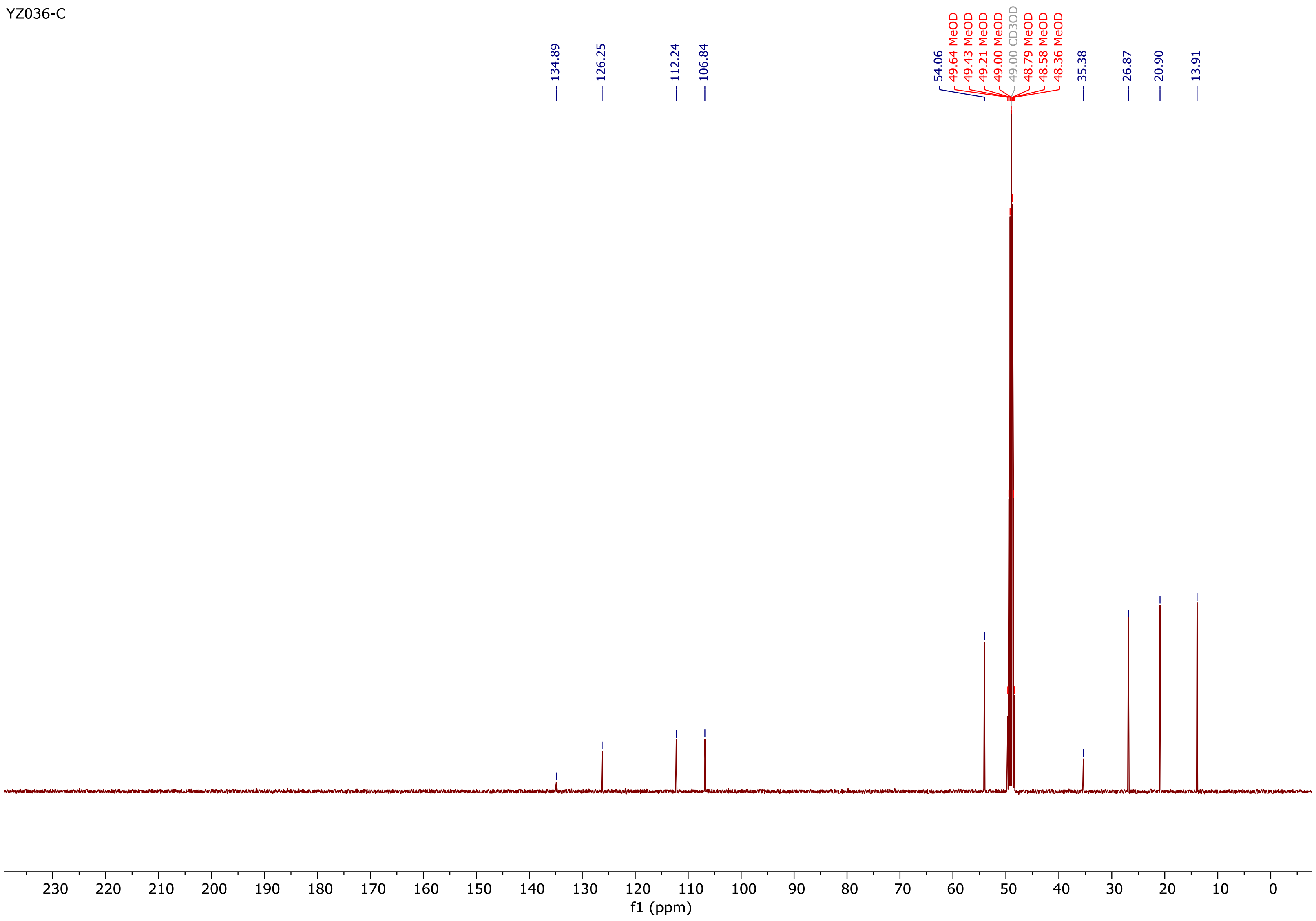

^{1}H NMR spectrum of **8** (400 MHz, MeOD)

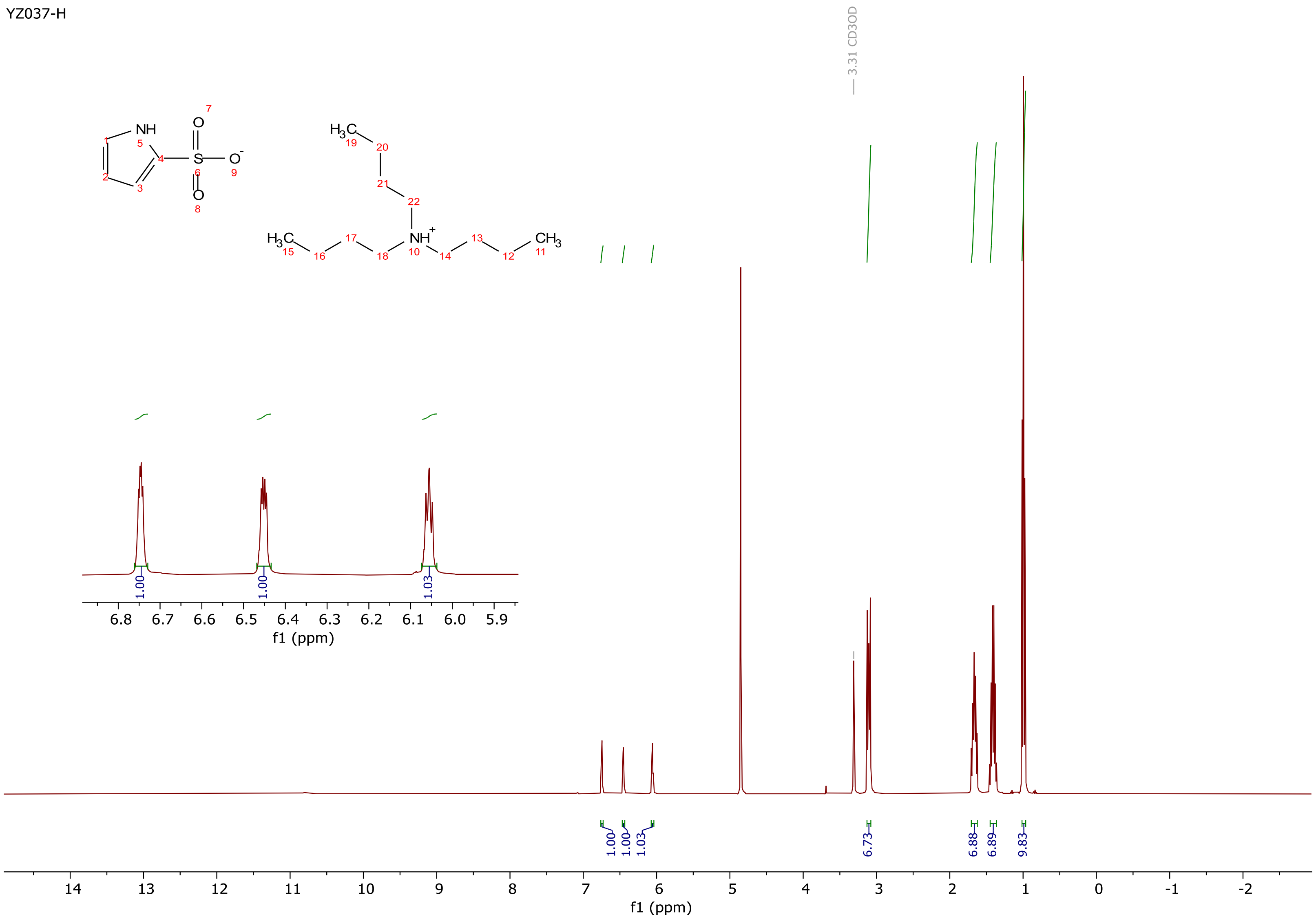

^{13}C NMR spectrum of **8** (101 MHz, MeOD)

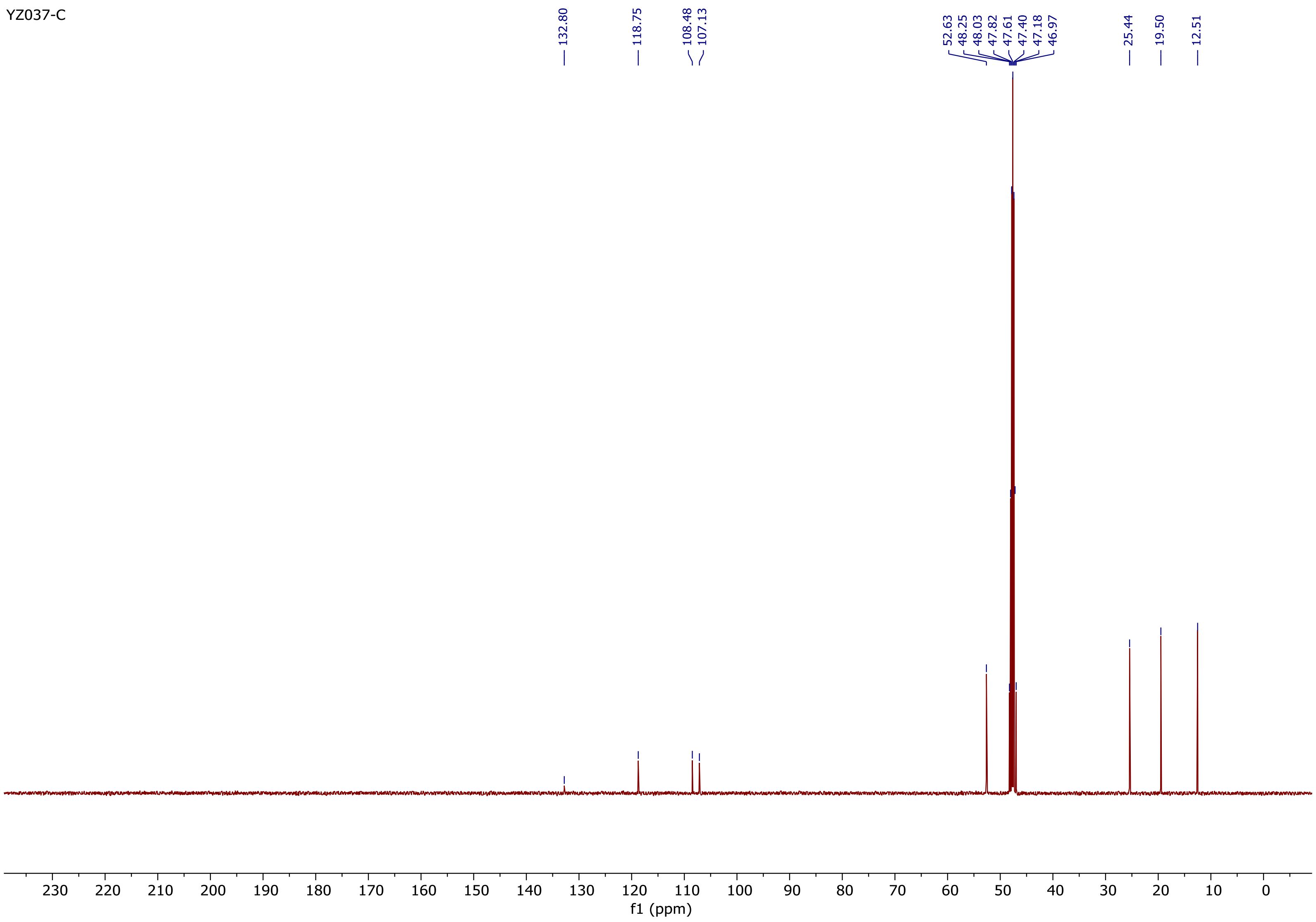

^{1}H NMR spectrum of **9** (400 MHz, MeOD)

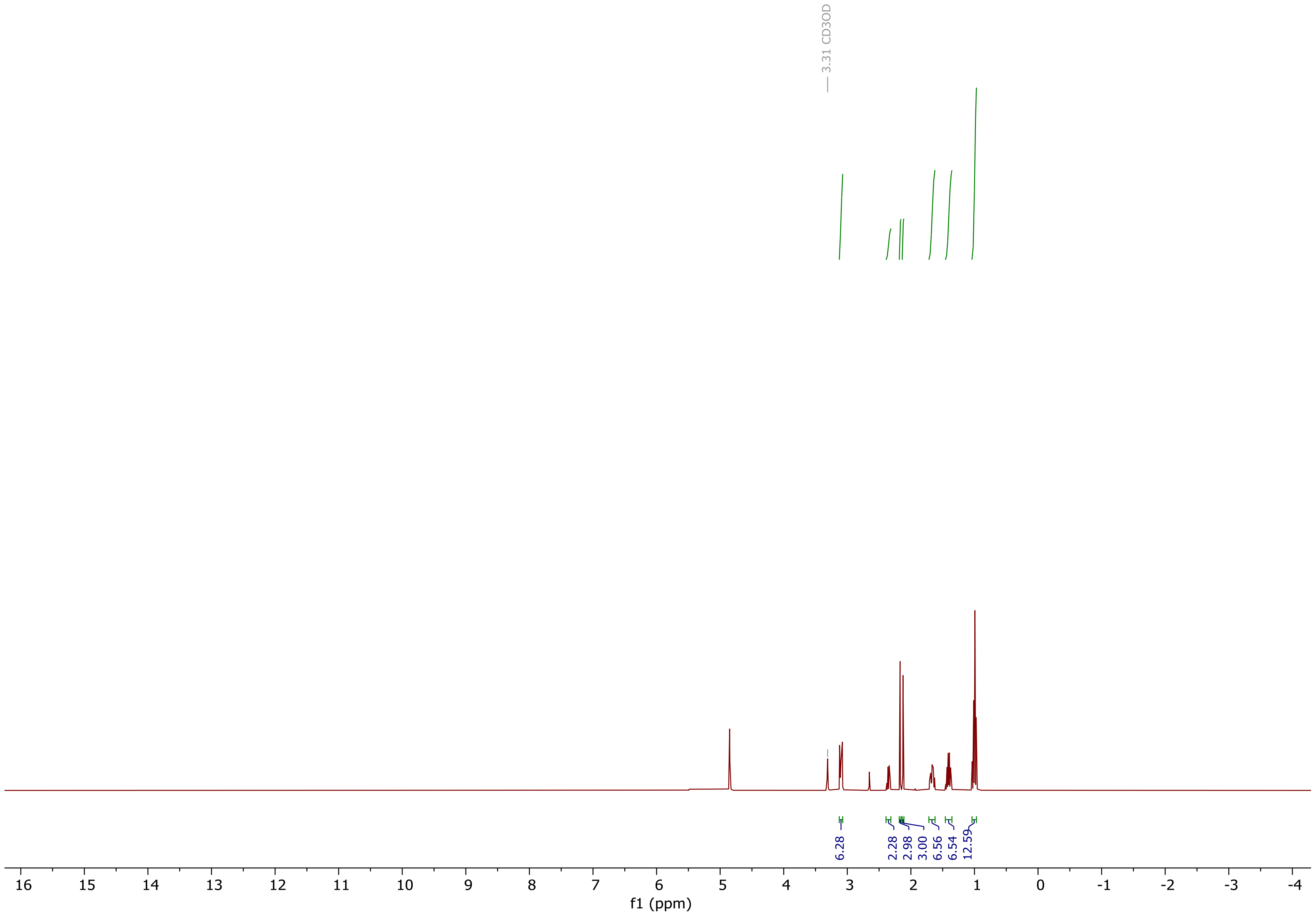

^{13}C NMR spectrum of **9** (101 MHz, MeOD)

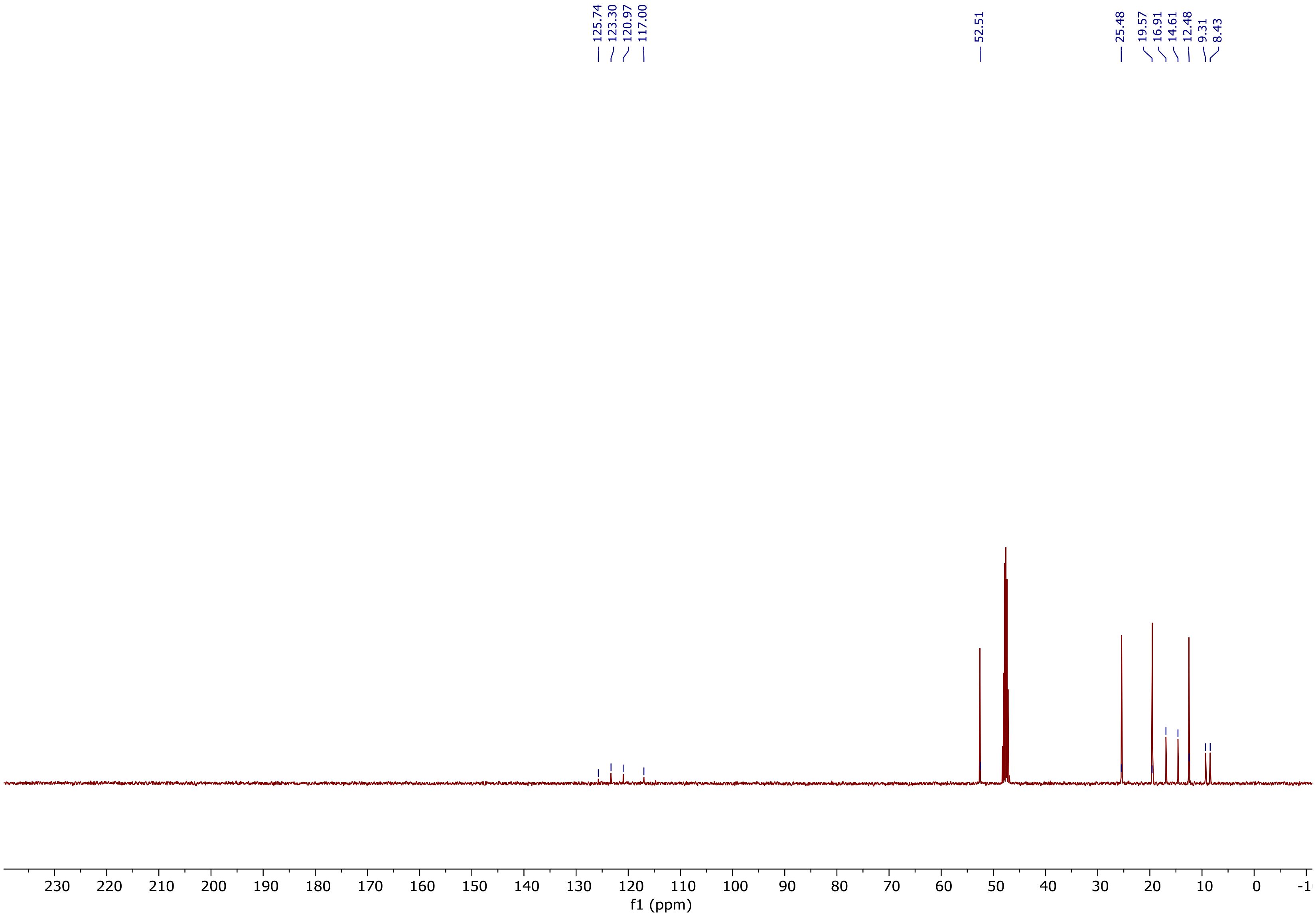

^{1}H NMR spectrum of **10** (400 MHz, MeOD)

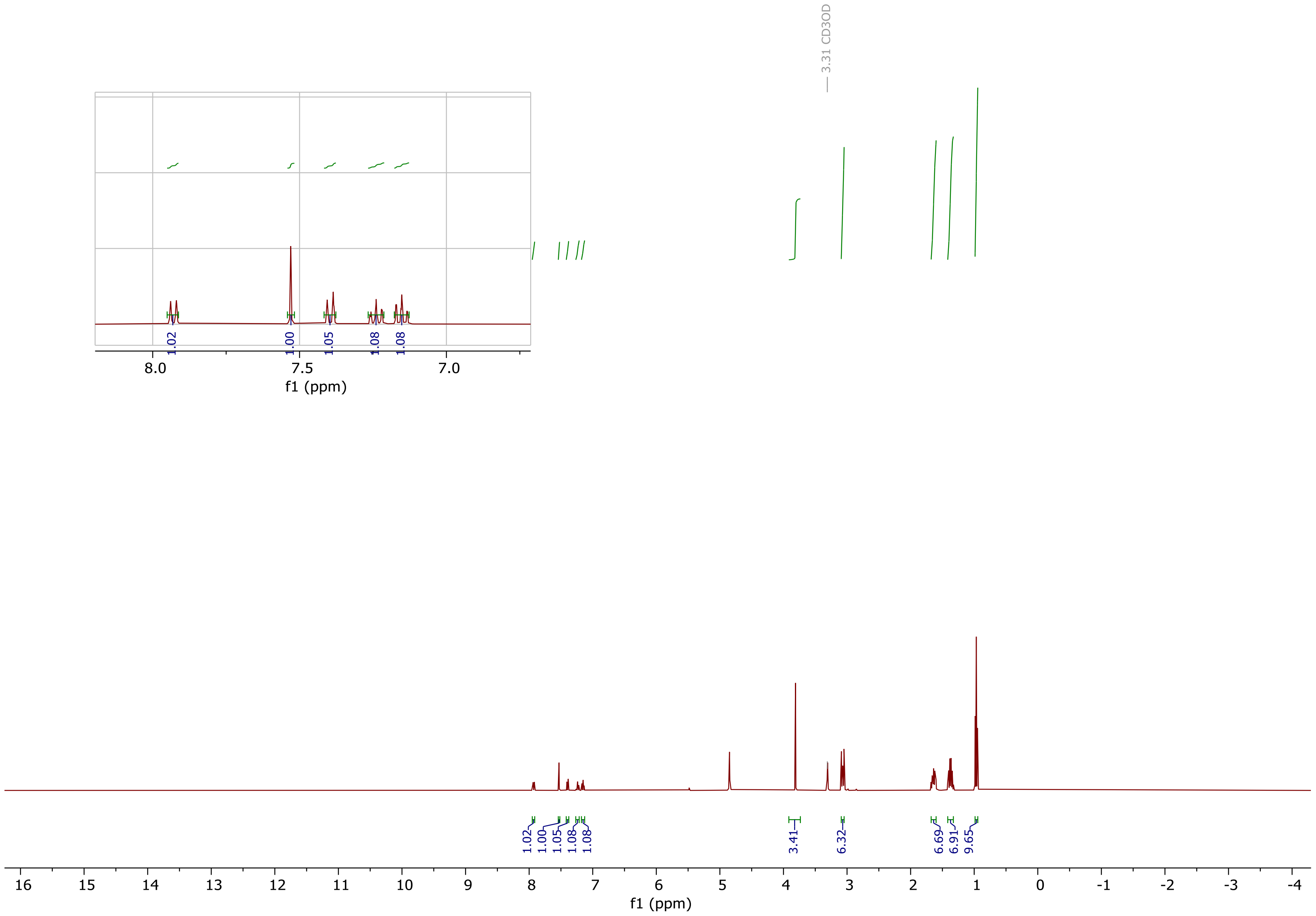

^{13}C NMR spectrum of **10** (101 MHz, MeOD)

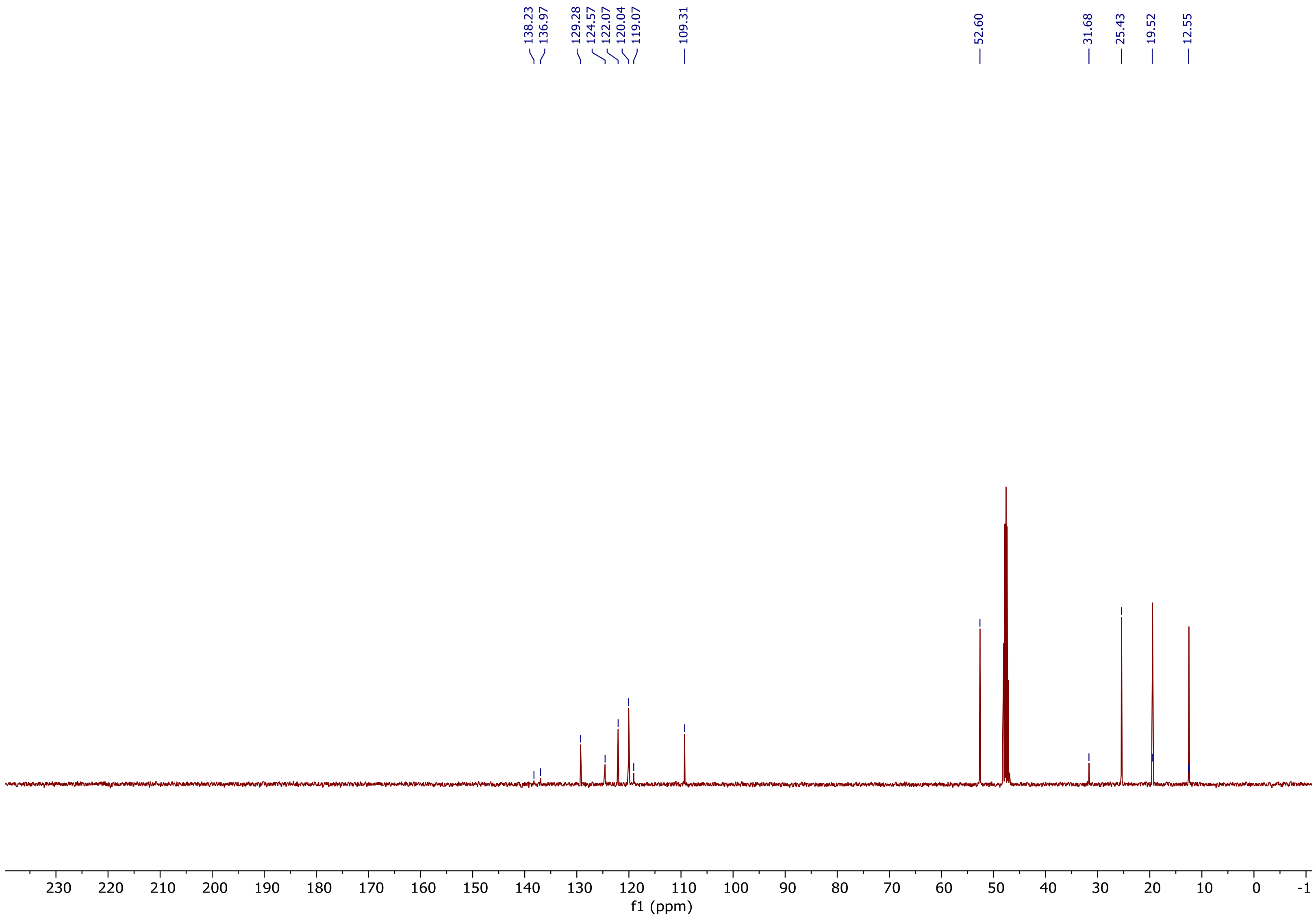

spectrum of **11** (400 MHz, MeOD)

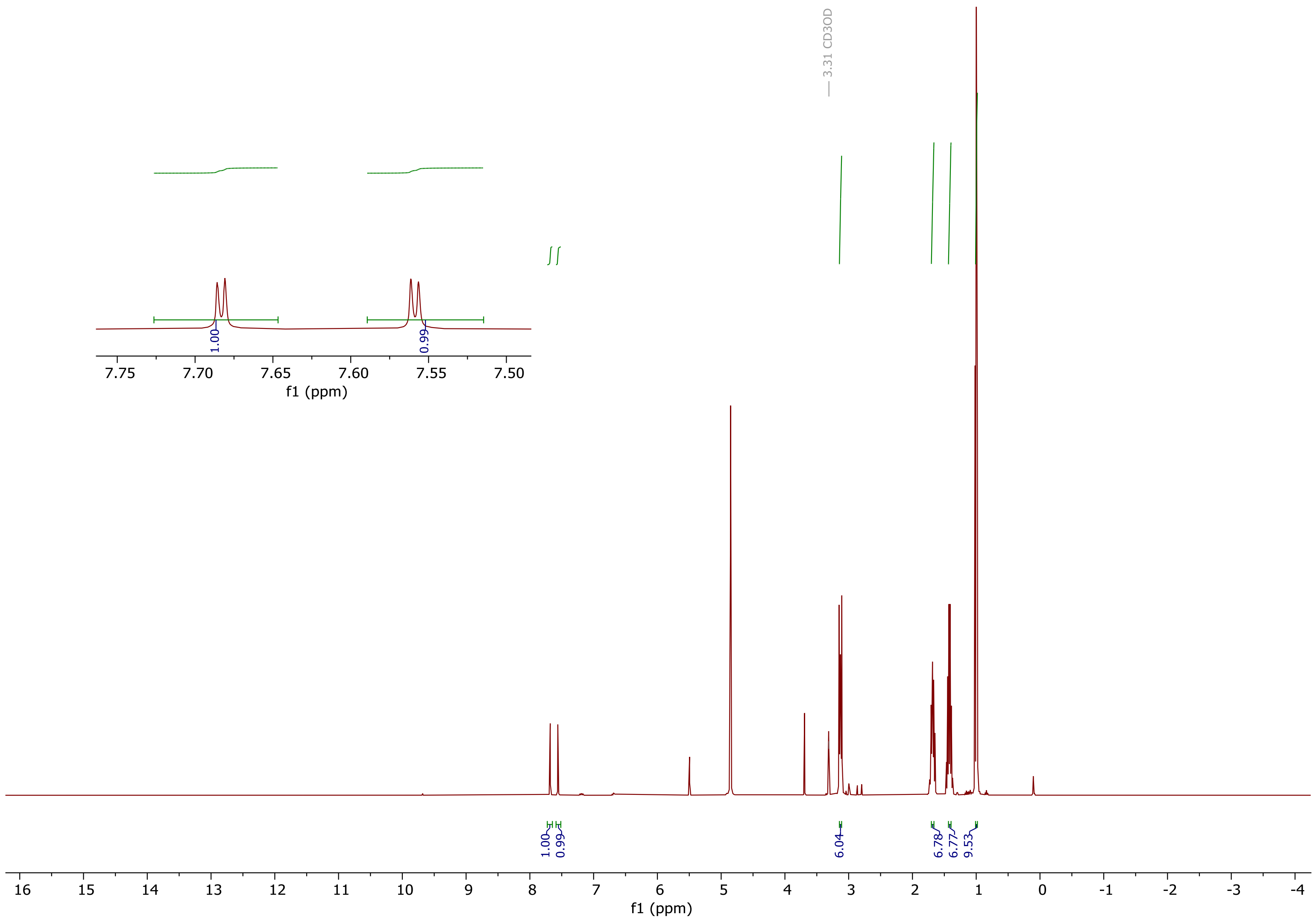

^{13}C NMR spectrum of **11** (101 MHz, MeOD)

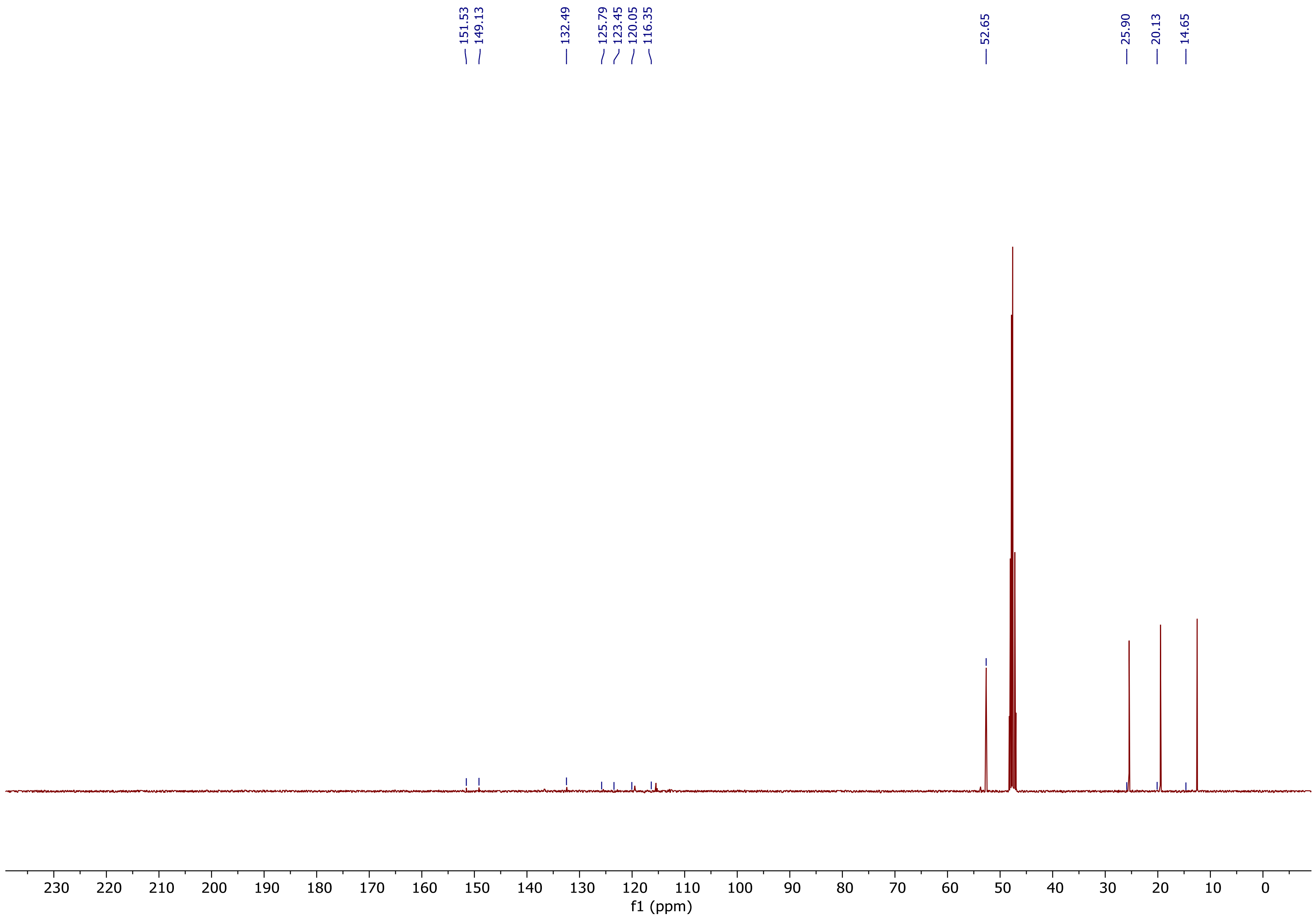

^{19}F NMR spectrum of **11** (377 MHz, MeOD)

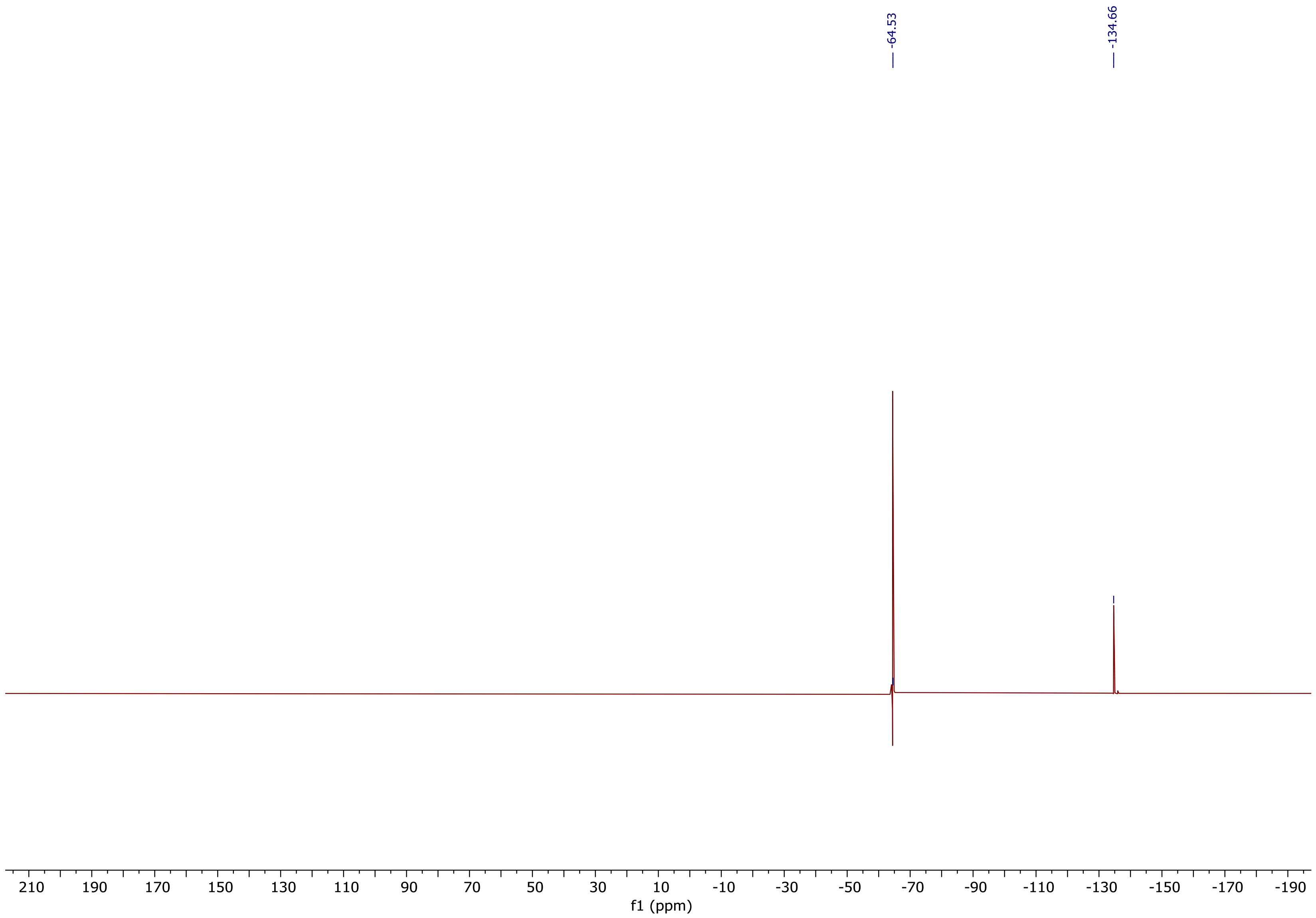

Deuteration Mechanism Experiment

Preparation of Aniline-d_2 (**d_2-1a**)

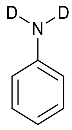

Aniline (5.1 g, 54.7 mmol) and deuterium oxide (10 g, 0.5 mol) were stirred vigorously under reflux at 120 °C for 2 h. The reaction mixture was then washed with D_2O (4 × 5 mL). The organic layer was distilled under vacuum to give the aniline-d_2 (4.9 g, 95 %). The aniline was >99 % double deuterated, as confirmed by ^{1}H-NMR.

^{1}H NMR (400 MHz, MeOD) δ_H 7.12 – 7.07 (m, 2H), 6.73 – 6.67 (m, 3H).

^{13}C NMR (101 MHz, MeOD) δ_C 147.1, 128.8, 118.1, 115.4

LRMS m/z (EI+) 95.07 ([M]$^+$, 100%)

^{1}H NMR Spectrum of **d_2-1a** (400 MHz, MeOD)

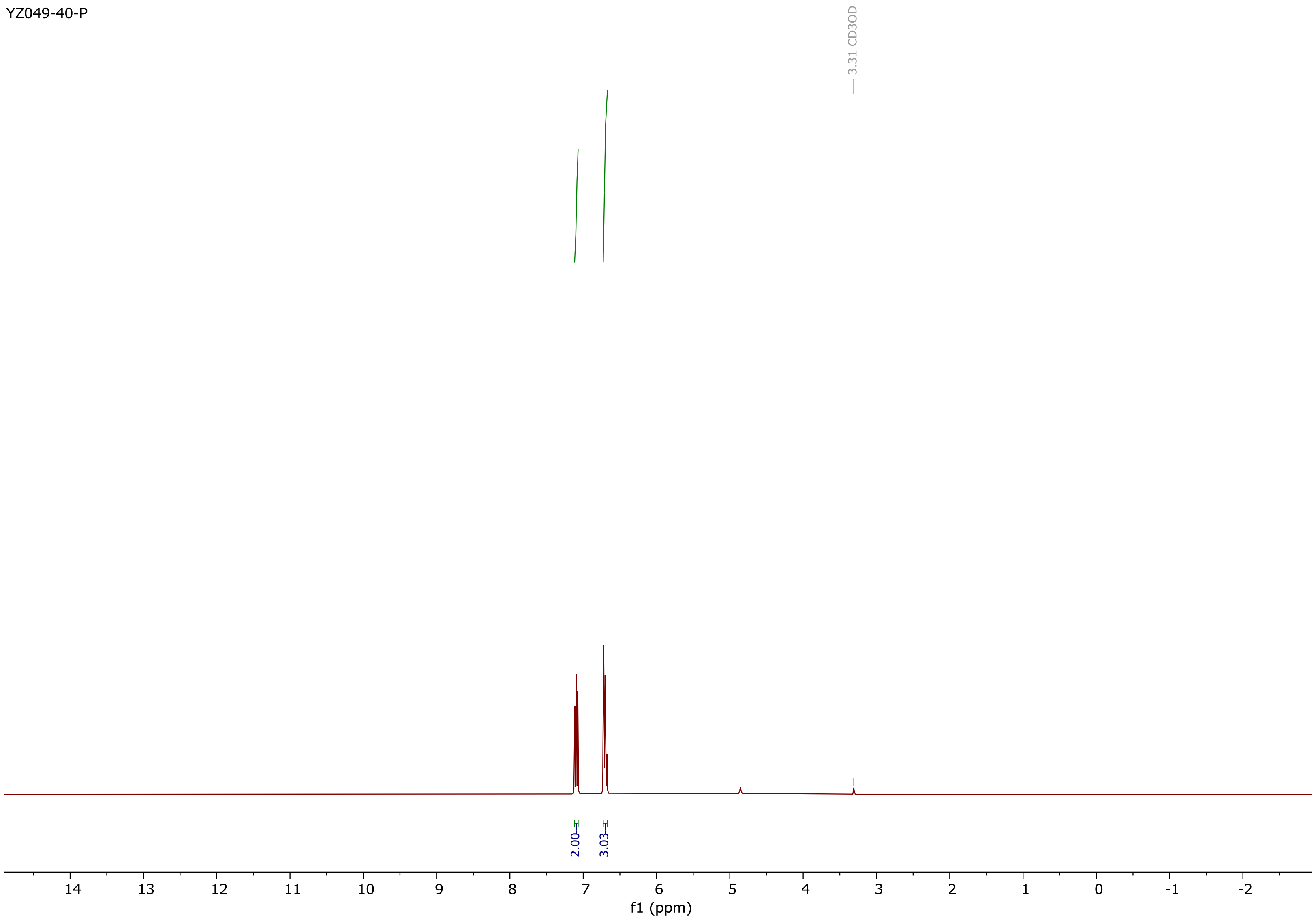

^{13}C NMR Spectrum of **d_2-1a** (101 MHz, MeOD)

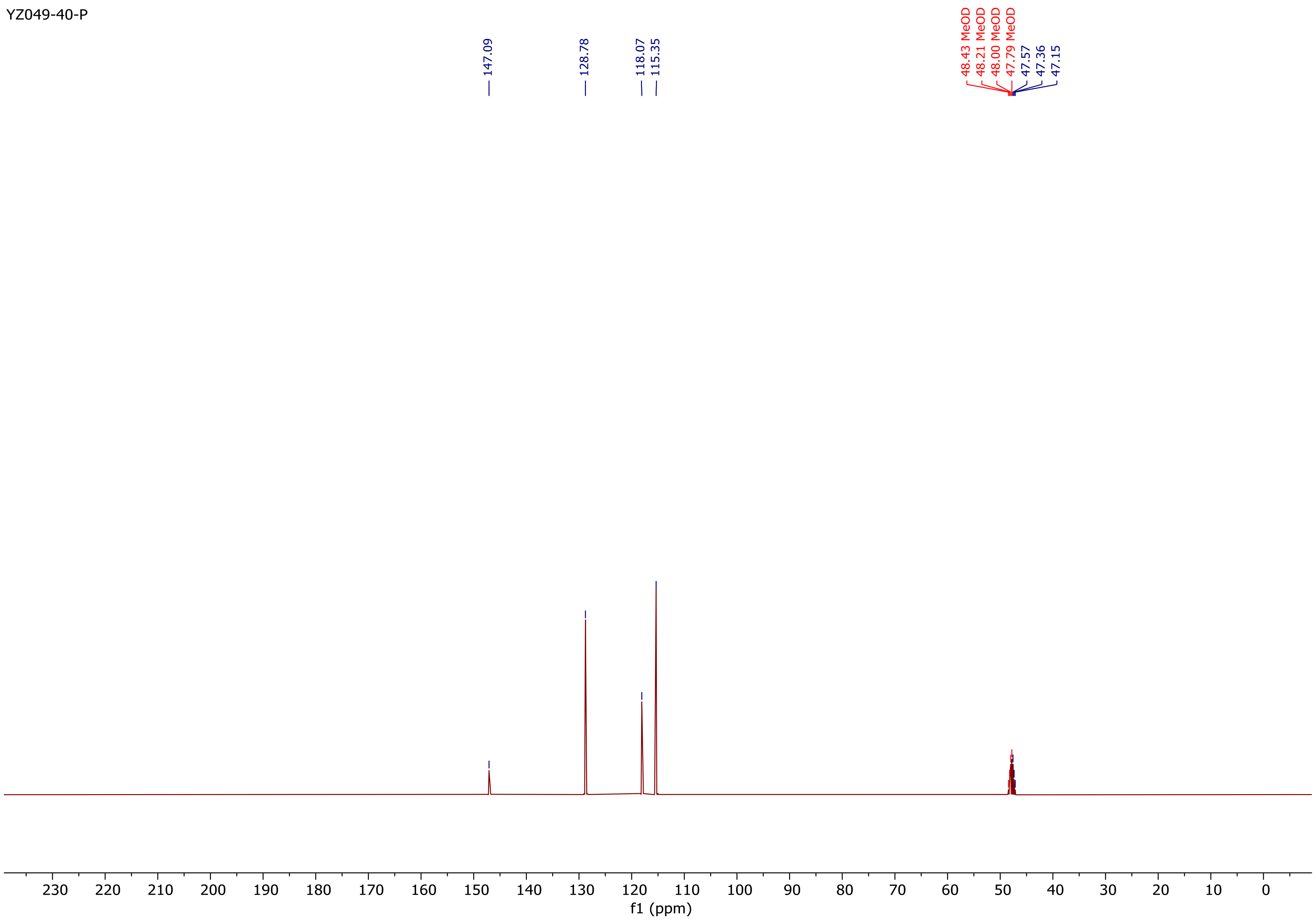

Tributylammonium-d deuterium(phenyl)sulfamate **(d_2-2a)**

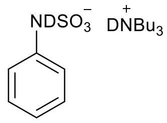

Following **general procedure 1**: Aniline-d_2 (0.09 mL, 1.0 mmol) and TBSAB (530 mg, 2.0 mmol) were dissolved in anhydrous MeCN (2.0 mL) and heated at 30 °C for 1 h. The title compound was extracted from cold water (10 mL) with EtOAc (4 × 50 mL) and washed with brine (10 mL). The organic layer was dried ($MgSO_4$), filtered, and evaporated *in vacuo* to afford the title compound as a white solid (326 mg, 91%).

M.P. 90-95 °C

^{1}H NMR (400 MHz, MeOD) δ_H 7.24-7.13 (m, 4H), 6.91-6.83 (m, 1H), 3.14-3.06 (m, 6H), 1.67 (ddt, *J* = 11.1, 7.1, 4.0 Hz, 6H), 1.41 (h, *J* = 7.3 Hz, 6H), 0.99 (t, *J* = 7.3 Hz, 9H)

^{13}C NMR (101 MHz, MeOD) δ_C 141.8, 128.3, 120.5, 117.4, 52.6, 25.4, 19.5, 12.5

LRMS m/z (ESI-) 173.04 ($[M-Bu_3ND]^-$, 100%)

HRMS m/z (ESI-) $C_6H_6NO_3S$ requires 173.0398, found 173.0401 ($[M-Bu_3ND]^-$)

^{1}H NMR spectrum of **d_2-2a** (400 MHz, MeOD)

deuterium sulfamate

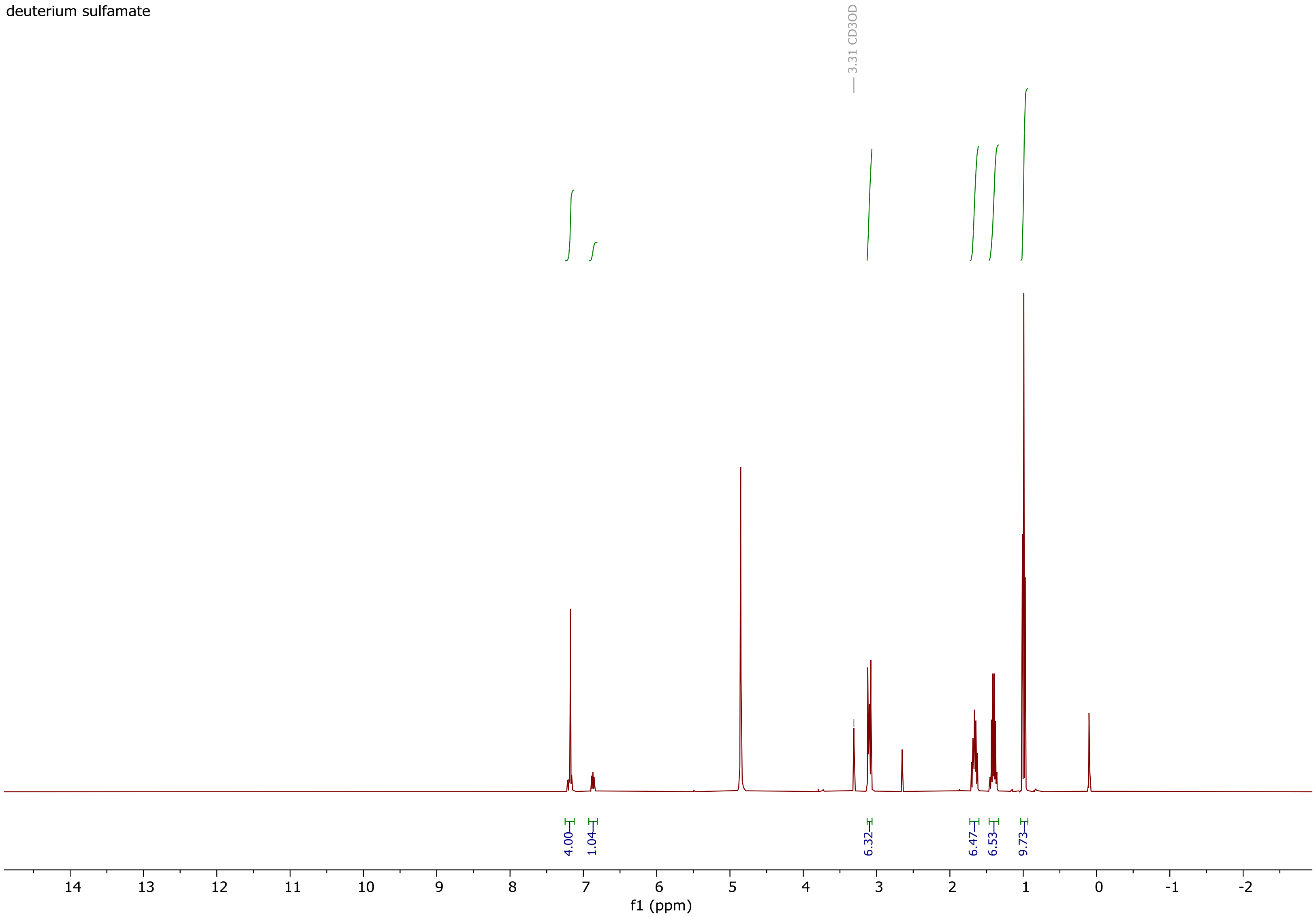

^{13}C NMR spectrum of **d₂-2a** (101 MHz, MeOD)

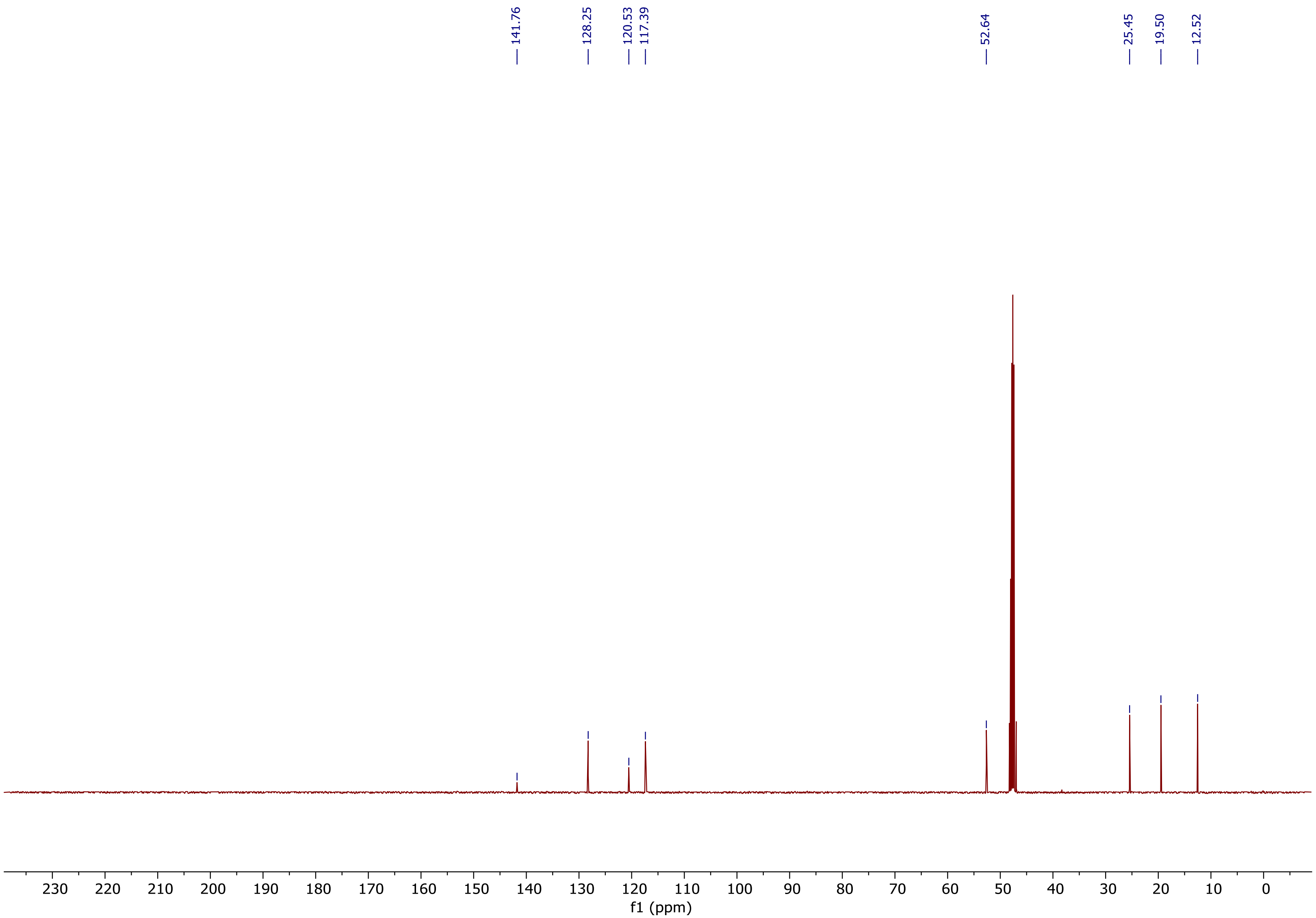

Tributylammonium 4-(amino-d_2)benzenesulfonate **(d_2-4a)**

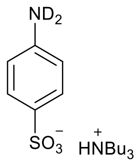

Following **general procedure 3**: Aniline-d_2 (0.18 mL, 2 mmol) and TBSAB (1.062 g, 4 mmol) were dissolved in DMF (2 mL) and heated at 120 °C under reflux for 24 h. The product was purified with (SiO_2; CH_2Cl_2/MeOH, 9.8:0.2, Rf = 0.2) to yield the title compound as dark brown oil (60.2 mg, 8%).

^{1}H NMR (400 MHz, MeOD) δ_H 7.62 (d, *J* = 8.4 Hz, 2H), 6.2 (d, *J* = 8.4 Hz, 2H), 3.17 - 3.08 (m, 6H), 1.69 (m, 6H), 1.42 (h, *J* = 7.4 Hz, 6H), 1.00 (t, *J* = 7.4 Hz, 9H)
^{13}C NMR (126 MHz, MeOD) δ_C 150.0, 133.3, 126.9, 113.3, 52.6, 25.4, 19.5, 12.5.
LRMS m/z (ESI-) 174.13 ($[M\text{-}Bu_3NH]^-$, 100%)
HRMS m/z (ESI-) $C_6H_6NO_3S$ requires 174.1220, found 174.1331 ($[M\text{-}Bu_3NH]^-$)

^{1}H NMR spectrum of **d_2-4a** (400 MHz,MeOD)

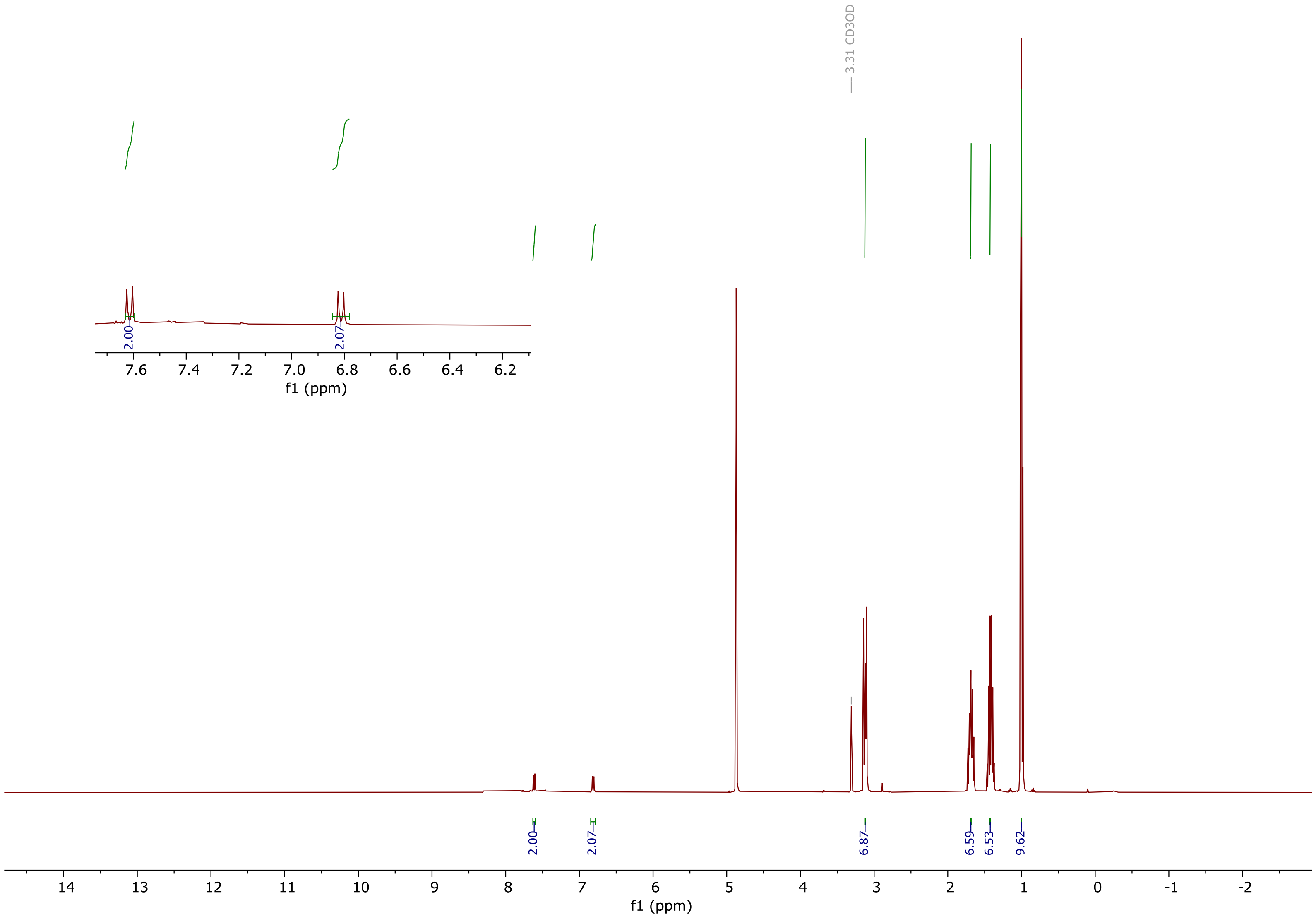

^{13}C NMR spectrum of **d_2-4a** (126 MHz,MeOD)

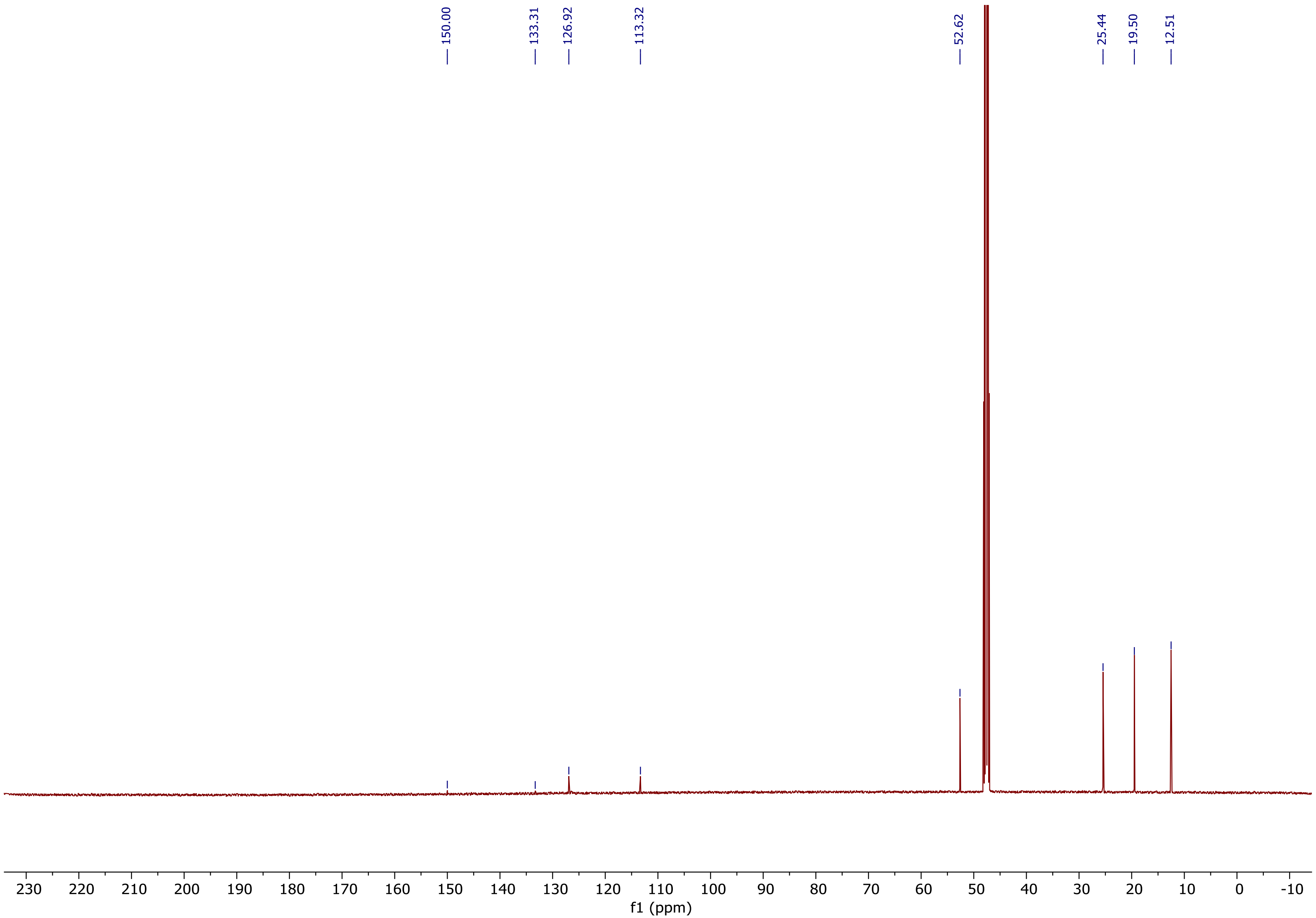

Sulfonation of Aniline-d$_2$

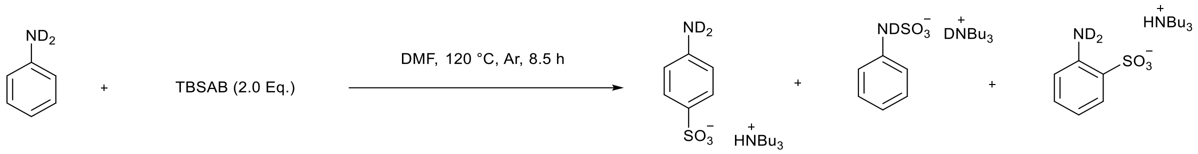

Following **general procedure 3**: Aniline or Aniline-d$_2$ (0.36 mL, 4 mmol) and TBSAB (2.124 g, 8.0 mmol) were dissolved in DMF (2 mL) and heated at 120 °C under reflux for 8.5 h. The crude product was purified with (SiO_2; CH_2Cl_2/MeOH, 9.4:0.6, R_f = 0.2) to yield the title compound as a clear yellow oil.

Table S1: % conversions

**Substrate**	**Conv. para-product**	**Conv. ortho-product**	**Unreacted starting material**	**Conv. sulfamate**
Aniline (**1a**)	58%	6%	14%	22%
Aniline-d$_2$ (**d$_2$-1a)**	24%	<1%	33%	42%

A

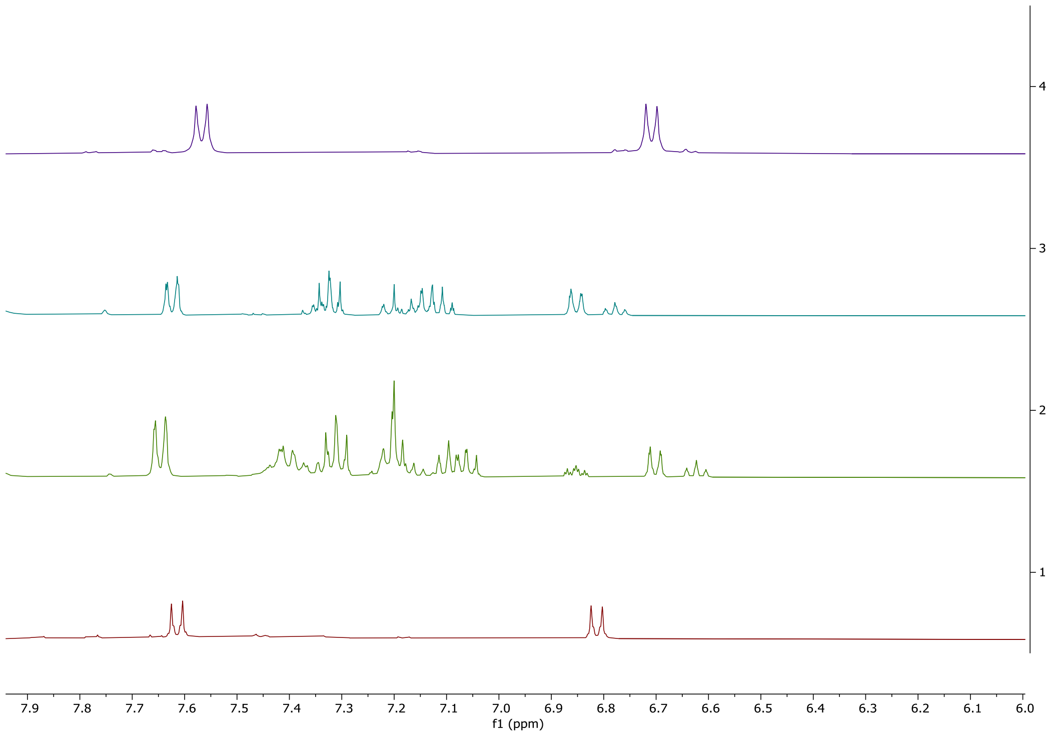

B

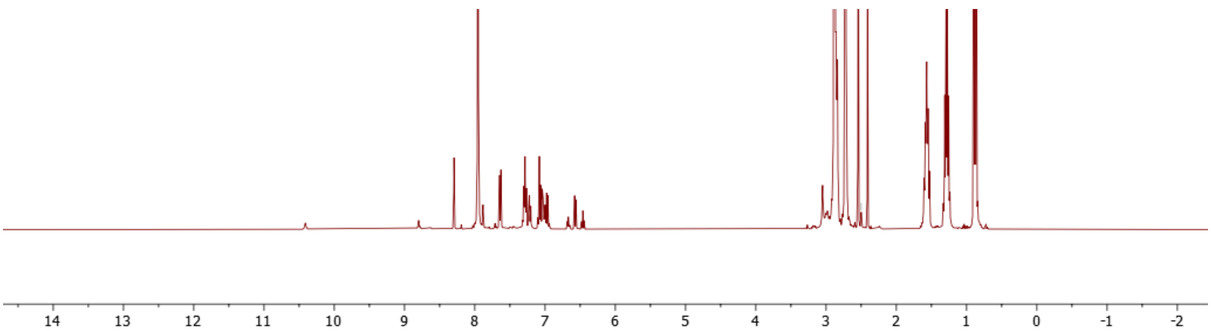

Figure S1. Stacked ^{1}H NMR spectroscopic analysis. **A**) Percentage conversion comparison of non-deuterated and deuterated para-products (**4a** and **d$_2$-4a**) in ^{1}H NMR spectra; 1) **d$_2$-4a**; 2) deuterated crude; 3) non-deuterated crude; 4) **4a**; and **B**) Deuterated crude ^{1}H NMR in d$_6$-DMSO (24 h)
